# Multiparameter Kinetic Analysis for Covalent Fragment Optimization by Using Quantitative Irreversible Tethering (qIT)

**DOI:** 10.1002/cbic.202000457

**Published:** 2020-08-07

**Authors:** Gregory B. Craven, Dominic P. Affron, Teresa Kösel, Tsz Lam M. Wong, Zoë H. Jukes, Chun‐Ting Liu, Rhodri M. L. Morgan, Alan Armstrong, David J. Mann

**Affiliations:** ^1^ Department of Life Sciences Imperial College London South Kensington Campus London SW7 2AZ UK; ^2^ Department of Chemistry Imperial College London Molecular Sciences Research Hub White City Campus Wood Lane London W12 0BZ UK

**Keywords:** Cdk2, covalent fragments, covalent inhibition kinetics, electrophile-sensitive inhibition, fragment-based drug discovery

## Abstract

Chemical probes that covalently modify cysteine residues in a protein‐specific manner are valuable tools for biological investigations. Covalent fragments are increasingly implemented as probe starting points, but the complex relationship between fragment structure and binding kinetics makes covalent fragment optimization uniquely challenging. We describe a new technique in covalent probe discovery that enables data‐driven optimization of covalent fragment potency and selectivity. This platform extends beyond the existing repertoire of methods for identifying covalent fragment hits by facilitating rapid multiparameter kinetic analysis of covalent structure–activity relationships through the simultaneous determination of *K*_i_, *k*_inact_ and intrinsic reactivity. By applying this approach to develop novel probes against electrophile‐sensitive kinases, we showcase the utility of the platform in hit identification and highlight how multiparameter kinetic analysis enabled a successful fragment‐merging strategy.

## Graphical abstract

**Breaking into fragments**: A biophysical platform for determining the complex structure–activity relationships of covalent fragments. By using this approach, a novel series of covalent fragments has been identified for targeting electrophile‐sensitive kinases; optimization is facilitated by covalent fragment merging.

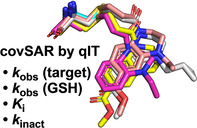

Covalent fragments are increasingly used as starting points for the development of chemical probes and therapeutics.[^1^, ^2^, ^3^] This approach offers the potential to target challenging protein interfaces and to achieve a high degree of target engagement more rapidly.^[4]^ Moreover, covalent binding of the target expedites chemical biology experiments, such as activity‐based protein profiling and fluorescence microscopy, that are invaluable in their ability to complement genetic studies.^[5]^ Covalent fragment libraries are typically comprised of low‐molecular‐weight bifunctional molecules, carrying amino acid‐reactive warheads, such as Michael acceptors and electrophilic heterocycles, and an unreactive specificity element, which directs the warhead selectively to the target of interest.[^6^, ^7^] Libraries are designed to maximize the chemical diversity of the specificity elements, using one or more class of warhead, and may include a flexible linker to target distal amino acids.[^6^, ^8^, ^9^] Target‐directed screening against recombinant proteins is generally achieved by intact protein mass spectrometry or fluorescence‐based tethering techniques to detect hit chemical starting points.[^10^, ^11^, ^12^] An alternative strategy is to use cell‐based screening of covalent fragments to identify new chemical starting points and/or protein targets through activity‐based protein profiling strategies.[^13^, ^14^, ^15^]

In general, targeted covalent protein inhibition occurs via a two‐step mechanism (Figure 1).^[16]^ First, the ligand binds to the target reversibly; this is mediated by noncovalent interactions between the specificity element and protein binding site, and is characterized by the thermodynamic binding constant *K*_i_. In the second step, the binding conformation of the ligand directs the warhead to react covalently with a proximal amino acid which is described by the kinetic inactivation constant *k*_inact_. Both during covalent fragment hit identification and subsequent optimization, the goal of maximizing 1/*K*_i_ and *k*_inact_ ensures that the observed rate of covalent target engagement (*k*_obs_) is rapid even at low ligand concentrations. However, interpreting covalent structure‐activity relationships (covSAR) is complicated by the variability of warhead intrinsic reactivity, often observed even within the same chemical series.^[17]^ Increasing intrinsic warhead reactivity translates into a faster *k*_inact_ and an apparent improvement in potency, but also gives rise to increased off‐target reactivity including increased glutathione (GSH) reactivity, which depletes the pool of active compound.^[18]^ With this in mind, a more nuanced approach to covSAR should aim to maximize *k*_inact_/*K*_i_ while simultaneously minimizing intrinsic warhead reactivity. However, the exhaustive kinetic experimentation required has proved a major obstacle in preventing community‐wide implementation of this kind of covSAR analysis during covalent inhibitor development. This is especially true for the initial screening stage where covalent fragment libraries often contain >1000 molecules.

**Figure 1:**
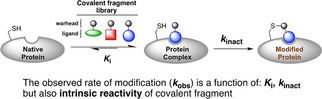
Two‐step mechanism for covalent protein inhibition.

Previously we described a fluorescence‐based platform for screening cysteine‐targeting covalent fragments called quantitative irreversible tethering (qIT).^[19]^ The method measures and compares the rate of reactions of individual fragments with the protein‐of‐interest (POI) and GSH (Figure 2a and b). Here reactivity with GSH, which is a cysteine‐containing tripeptide that lacks significant secondary structure, represents a measure of intrinsic reactivity and hit fragments are those that react significantly faster with the POI than with GSH, as quantified by the rate enhancement factor (REF; Figure 2c). The key advantage of screening by REF analysis, is that qIT can be carried out in high throughput and does not suffer from high false‐positive/‐negative hit rates because it accounts for intrinsic fragment reactivity.

**Figure 2:**
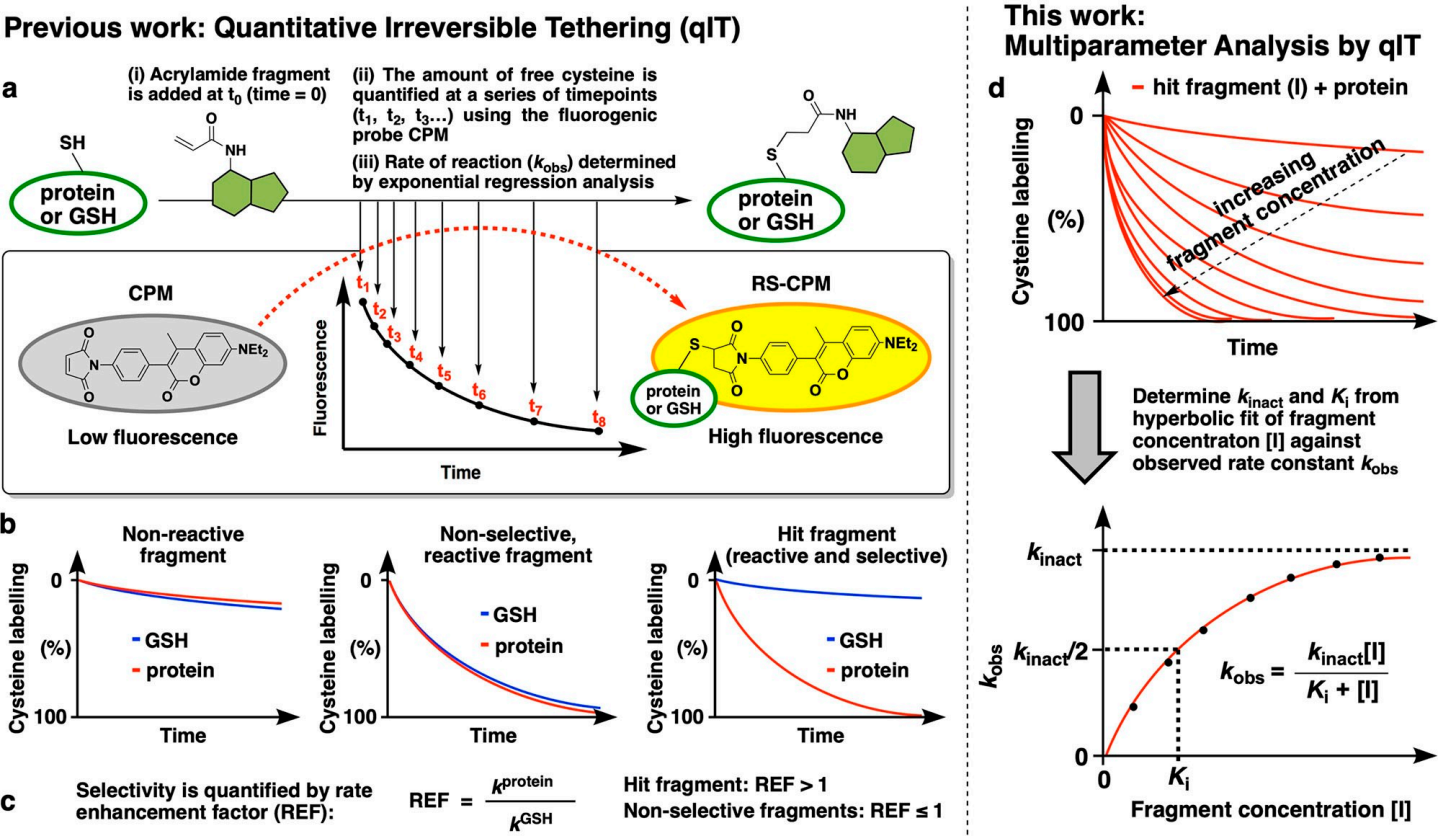
Comparison of previous and current applications of Quantitative irreversible tethering (qIT). a) Overview of rate determination. A cysteine‐containing biomolecule is treated with covalent fragments under pseudo‐first‐order conditions. Reaction progress is followed by discrete measurement of free cysteine concentration by using the fluorogenic probe CPM, and rate constants (*k*_obs_) are derived from exponential regression analysis. b) Comparison of the reactivity profile of each fragment with the POI and GSH typically classes them as unreactive, reactive but non‐selective or reactive and selective. c) Quantification of kinetic POI/GSH selectivity facilitates hit identification. d) The observed rate of reaction between the POI and hit fragment is determined at a range of ligand concentrations by using qIT. Subsequent hyperbolic regression analysis is used to derive *K*_i_ and *k*_inact_ to facilitate comprehensive covSAR analysis.

Herein, we report further application of the qIT platform to undertake comprehensive kinetic analysis of covalent inhibitor binding to simultaneously derive REF, *K*_i_ and *k*_inact_ for covSAR profiling (Figure 2d). We demonstrate that by determining the concentration dependency of the rate of cysteine modification by using qIT, both *K*_i_ and *k*_inact_ can be derived by using hyperbolic‐regression analysis. Moreover, using electrophile‐sensitive kinases as a model system, we use REF analysis to identify hit covalent fragments and then apply a structure‐guided fragment‐merging approach in combination with our comprehensive kinetic analysis to optimize compound potency.

As a method for conducting allele‐specific chemical genetic investigations, non‐native cysteine residues can be introduced onto the surface of a protein, against which electrophilic probes are developed, such that chemical inhibition can be achieved with a level of selectivity that is typically impossible to achieve in a native system (Figure 3a).^[20]^ This electrophile‐sensitive (ES) strategy has previously been successfully applied to c‐Src, Aurora A and EphB1 kinases by using established adenosine mimetics as starting scaffolds.[^21^, ^22^] Here, we looked to apply the ES strategy to study cyclin‐dependent kinase 2 (Cdk2), which is a clinically important serine/threonine kinase in oncology, its activity is important for driving cell replication and is dependent upon association with a cyclin protein.^[23]^ The high degree of active‐site homology within the Cdk family, has meant that developing Cdk2‐specific inhibitors has proved elusive and, as such, selective inhibitors of electrophile sensitive Cdk2 would be highly valuable tools.^[24]^

**Figure 3:**
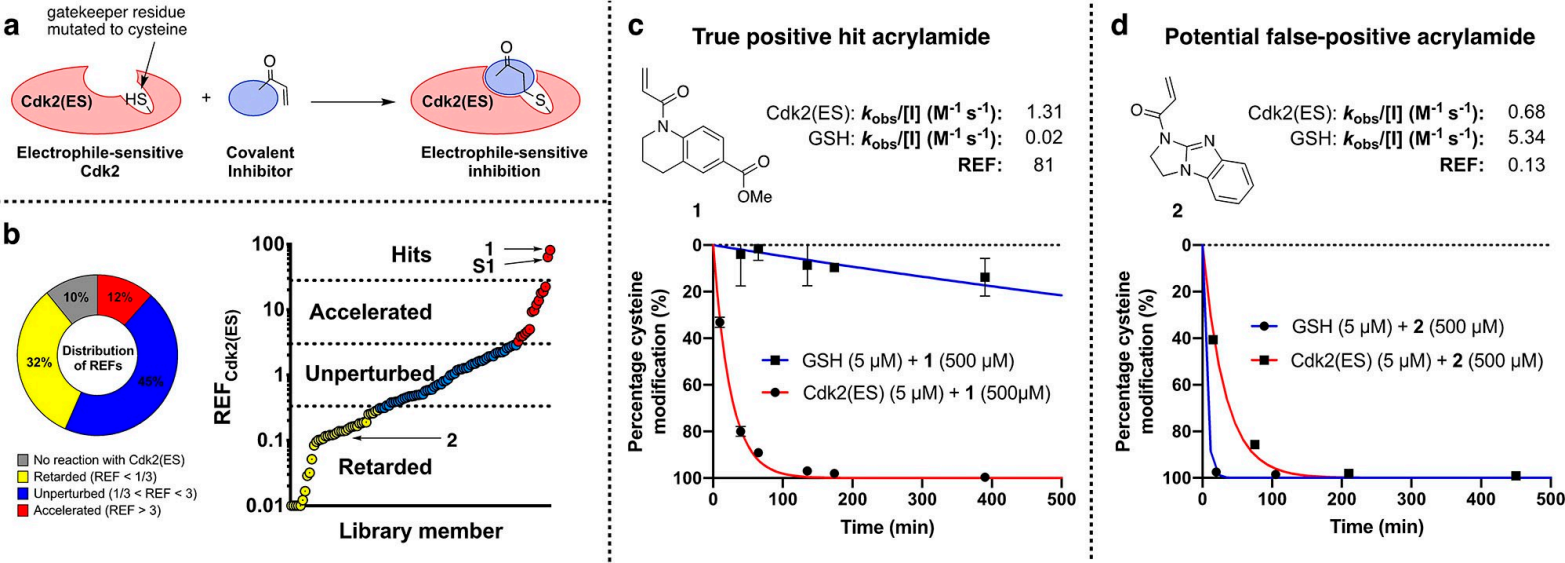
Screening cascade to identify covalent fragments targeting Cdk2(ES). a) Gatekeeper cysteine (F80C) ES strategy for allele‐specific inhibition of Cdk2. b) Distribution of rate‐enhancement factors for the covalent fragment library screened against Cdk2(ES) and GSH, highlighting hits **1** and **S1** and potential false positive **2**. c), d) qIT data for acrylamides **1** and **2** (0.5 mM) in reaction with Cdk2(ES) or glutathione (5 μM). Fluorescence intensity is converted into percentage cysteine modification by normalizing to DMSO control=0 %, no thiol=100 %.

To generate an ES Cdk2 construct (Cdk2(ES)), we mutated the gatekeeper phenylalanine residue to cysteine (F80 C) to enable covalent binding in the active site. Additionally, Cdk2 carries a surface‐exposed cysteine (C177) in an allosteric pocket, and this was mutated to alanine to facilitate compatibility with qIT, which relies on the presence of a single surface‐exposed cysteine residue. Crystallization of this construct confirmed that the engineered cysteine was in a reactive conformation, being surface exposed and pointing towards the kinase hinge region (Figure S1a in the Supporting Information). Comparison of this structure with the Cdk2(WT) (PDB ID: 4EK3) structure confirmed that no significant active site distortion had taken place (RMSD=0.501 Å, Figure S1b). Next, we investigated whether the class of covalent inhibitors that were previously developed against c‐Src(ES) tyrosine kinase, also containing a gatekeeper cysteine mutation, could be used as starting points for inhibitor development.^[14]^ However, they showed very low activity against Cdk2(ES) (data not shown).

Therefore, we set out to develop novel classes of electrophile sensitive kinase inhibitors using a covalent fragment approach. Accordingly, we screened a 138‐member library of diverse cysteine‐reactive fragments at 500 μM against Cdk2(ES) and GSH by using the qIT platform and monitored the kinetics of cysteine‐modification over 24 hours. The fragments showed a range of reactivity profiles, with only 10 % displaying no reactivity towards Cdk2(ES) (Figure 3b). Using three standard deviations over the geometric mean REF as the statistical cut‐off for defining hit fragments (REF >27.9) yielded two acrylamide fragments, tetrahydroquinoline **1** (REF=81) and pyrimidine **S1** (REF=64) for further investigation (Figures 3c and S2). Interestingly, several of the acrylamide fragments, such as aminoimidazole **2** (REF=0.13), reacted with Cdk2(ES) at similar rates to hits **1** and **S1** but could be readily discarded because of their high intrinsic reactivity with GSH, thus highlighting the importance of REF analysis in distinguishing true positives from potential false positives (Figure 3d).

To orthogonally validate the results from the qIT assay, we undertook a mass spectrometric study to obtain a direct measurement of both the extent and site of covalent fragment modification. First, intact protein mass spectrometry confirmed that acrylamide **1** fully modifies Cdk2(ES), but not Cdk2(WT), within 2 hours at 500 μM (Figure 4a). The absence of higher‐molecular‐weight signals demonstrates that the protein is mono‐modified, showing that this modification is specific in nature. To confirm C80 as the site of modification, the **1**‐Cdk2(ES) complex was digested with trypsin and the resulting peptides analysed by MALDI mass spectrometry. Compared to unmodified Cdk2(ES), **1**‐Cdk2(ES) showed a new tryptic peptide with *m/z* 1866.01, which corresponds to that expected for the C80 containing peptide modified with acrylamide **1** (Figure S3a). Tandem mass spectrometric analysis of this ion enabled confirmatory sequencing of the peptide and unambiguous assignment of C80 as the site of modification (Figure 4b). The same intact‐protein mass spectrometry and tryptic digest MALDI analysis was then performed on the **S1**‐Cdk2(ES) complex, which also showed complete mono‐modification of Cdk2(ES) and similarly defined C80 as the sole site modification (Figures S2b and S3b‐d).

**Figure 4:**
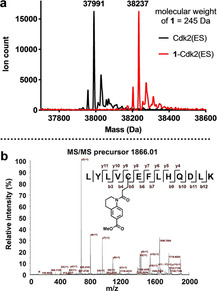
a) Intact‐protein mass spectrum of Cdk2(ES) before and after incubation with acrylamide **1** (0.5 mM) for 2 h showing complete mono‐modification (ΔMW=246 Da). b) MALDI‐TOF/TOF spectrum of the C80‐containing tryptic peptide (precursor ion *m/z* 1866) of **1**–Cdk2(ES).

Next we undertook biochemical validation of the electrophile sensitive approach using a spectrophotometric kinase assay. In this experiment the activity of Cdk2 is activated both by phosphorylation and interaction with cyclin A. The catalytic turnover of ATP is coupled to the the oxidation of NADH by lactate dehydrogenase (LDH) and pyruvate kinase (PK), which is monitored by absorbance at 340 nm. Encouragingly, we found that active pCdk2(ES)/cyclin A holoenzyme has equivalent kinase activity to pCdk2(WT)/cyclin A (Figure 5). Moreover, we showed that acrylamides **1** and **S1** confer complete irreversible inhbition of Cdk2(ES), which is not recovered after dialysis of the ligand (Figure S4).

**Figure 5:**
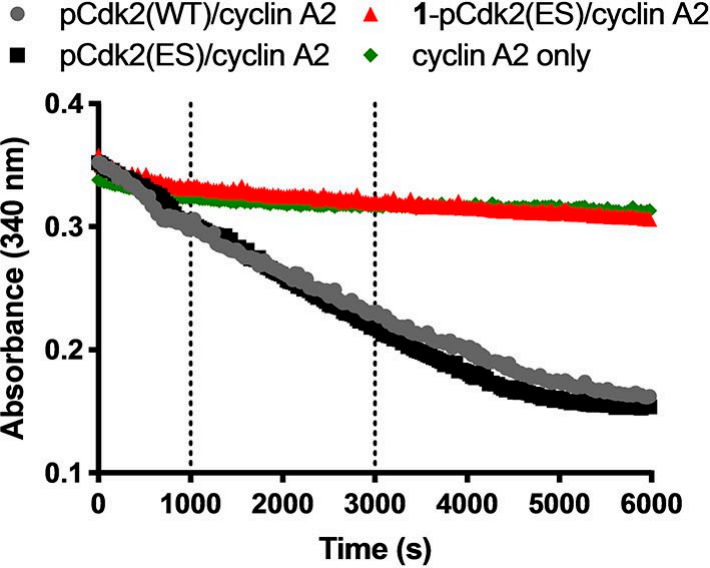
*In vitro* kinase activity for pCdk2(WT/ES)/cyclin A2 holoenzme, demonstrating complete inhibition of pCdk2(ES) by treatment with 0.5 mM acrylamide **1** for 2 h and then subsequent dialysis to remove excess ligand: The holoenzymes were then incubated with peptide substrate, ATP, NADH, PEP, LD and PK at 37 °C, and the absorbance was measured over time in clear 384‐well plates.

To enable structure‐guided ligand optimization, we crystallized the **1**‐Cdk2(ES) and **S1**‐Cdk2(ES) complexes which diffracted to high resolution and further confirmed that the acrylamides had labelled Cys80 (Figures 6a and S5). The ligand electron density was more clearly defined for acrylamide **1** than **S1** and, as such, we focussed on acrylamide **1** for further investigation. The tetrahydroquinoline scaffold of acrylamide **1** is orientated into the adenine binding site, which is consistent with the observed inhibition of kinase activity, and the methyl ester forms a hydrogen bond with the hinge via the backbone amide of Leu83. Interestingly, the acrylamide carbonyl itself is hydrogen bonded to the ϵ‐amine of Lys33, and this interaction likely plays a role in activating the acrylamide towards 1,4‐addition. This mechanism of warhead activation by an adjacent lysine has recently by reported for KRas(G12 C) inhibitors and is postulated to work both by orientating the warhead for reaction and stabilizing the reaction transition state by neutralizing the transient negative charge on the acrylamide.^[25]^

**Figure 6:**
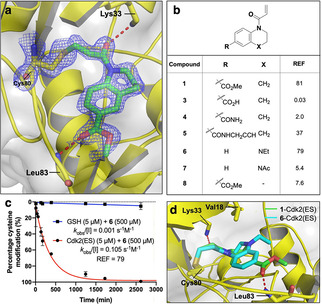
Structure‐guided ligand optimization. a) Crystal structure of the **1**‐Cdk2(ES) conjugate (resolution: 1.77 Å, PDB ID: 5OSM). The 2*F*_o_−*F*_c_ electron‐density map (blue) is contoured at 1*σ* around Cys80 (yellow) and the ligand (green), with the hydrogen bonds to L83 and K33 shown in red. b) Covalent SAR for fragment analogues by REF analysis. c) qIT data for acrylamide **6** (0.5 mM) in reaction with Cdk2(ES) or glutathione (5 μM). Fluorescence intensity is converted into percentage cysteine modification by normalizing to DMSO control=0 %, no thiol=100 %. d) Crystal structure of the **6**‐Cdk2(ES) complex (cyan; resolution 1.72 Å, PDB ID: 5OO3) aligned against **1**–Cdk2(ES) (green).

Next, we synthesized a small collection of fragment analogues, which were screened by REF‐based qIT analysis (Figure 6b). First, the effect of substitution of the methyl ester was investigated. Whereas carboxylic acid **3** and primary amide **4** led to complete loss of activity, the propargyl amide **5** was well tolerated. Indoline **8** gave a tenfold loss in REF, thus suggesting a preference for 6/6‐ over 5/6‐fused ring systems for proper orientation of the warhead. Remarkably, the related tetrahydroquinoxaline **6** maintained a similar REF despite lacking any potential for hinge binding through the H‐bonding capability of the aryl methyl ester. The importance of the new alkyl quinoxaline nitrogen was further supported by the loss of activity for *N*‐acetyl analogue **7**. Interestingly, although acrylamides **1** and **6** have similar REF values, they have quite distinct kinetic profiles, with acrylamide **6** displaying both a reduction in Cdk2(ES) reactivity and intrinsic reactivity, of which the latter is attributable to the more electron rich nature of anilines compared with aryl esters (Figure 6c).

Intriguingly, crystallization of the **6**‐Cdk2(ES) complex revealed that the 6/6 ring system was bound in the same orientation as that of tetrahydroquinoline **1**, despite the lack of any hinge binding interactions. The *N*‐ethyl group makes a new hydrophobic interaction with Val18 which might serve to further orientate the fragment for covalent modification of Cys80 (Figures 6d and S6). Therefore, we proposed that merging acrylamides **1** and **6**, to combine the Leu83‐directed H‐bonding ability of the ester and the Val18‐directed hydrophobic vector accessed by the tetrahydroquinoxaline scaffold, would generate an even more potent analogue.

Accordingly, we synthesized a collection of acrylamides based around the merged fragment scaffolds using an established aryl‐1,2‐diamine cyclization strategy (Figure S7).^[26]^ Pleasingly, we found that the merged acrylamide **9** did indeed have an improved selectivity profile, reacting with Cdk2(ES) six times faster than acrylamide **1** (Figure 7a). The resulting REF=182 is more than the sum of the parent compounds’, and this synergy is indicative of a successful fragment merge, of which few examples have been described in the context of covalent fragments.[^1^, ^27^] The methyl ester appears to largely dictate the intrinsic reactivity, with acrylamides **1** and **9** reacting with GSH at similar rates and significantly faster than acrylamide **6**. Crystallization of the **9**‐Cdk2(ES) complex confirmed that both the Leu83 H‐bonding and the Val18 hydrophobic interactions were simultaneously engaged by quinoxaline **9** (Figure S8).

**Figure 7:**
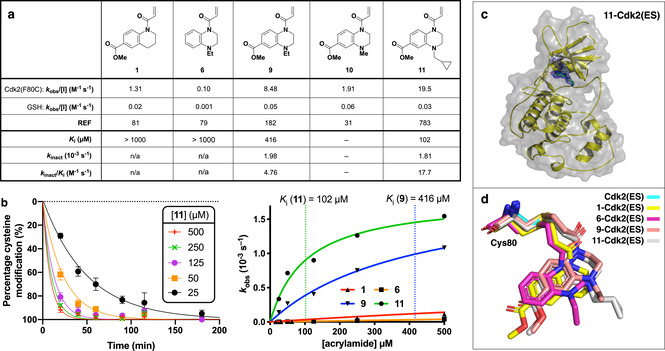
Kinetic analysis of merged fragments. a) Kinetic analysis of acrylamides **1**, **6**, **9**, **10** and **11** determined by qIT. Concnetration‐dependent analysis was used to derive *K*_i_ and *k*_inact_. b) Crystal structure of the **11**‐Cdk2(ES) conjugate (resolution: 1.66 Å, PDB ID: 6YL1). The 2*F*_o_−*F*_c_ electron density map (blue) is contoured at 1*σ* around Cys80 (yellow) and the ligand (green), with the hydrogen bonds to L83 shown in red. c) Concentration‐dependent qIT analysis. Left: Representative qIT data: acrylamide **11** (25–500 μM) reacting with Cdk(ES) (5 μM; *n*=2, error bars denote SD). Fluorescence intensity is converted into percentage cysteine modification by normalizing to DMSO control=0 %, no thiol=100 %. Right: Hyberbolic fitting of *k*_obs_ against concentration of acrylamides yields *K*_i_ and *k*_inact_. d) Overlay of crystal binding poses of Cys80 conjugated to acrylamides **1**, **6**, **9** and **11**, after alignment of the global protein structre.

Altering the quinoxaline nitrogen substituent proved to be key to improving kinetic selectivity, with the shorter *N*‐methyl analogue **10** being less Cdk2(ES) selective, whereas the bulkier *N*‐methylcyclopropyl analogue **11** showed a significant improvement in REF. The crystal structure of **11**‐Cdk2(ES) revealed that the methyl cyclopropane functionality extends beyond Val18 into the ribose binding site where it picks up additional hydrophobic interactions, which presumably account for the increase in potency (Figures 7b and S9).

Next we sought to carry out comprehensive kinetic analysis of the covalent binding to better understand the covSAR trends. Accordingly, the concentration dependency for the rate of reaction between Cdk2(ES) and key acrylamides **1**, **6**, **9** and **11** were determined using qIT (Figure 7c). To maintain pseudo‐first‐order reaction kinetics and stay within the solubility limits of the fragments, ligands were titrated from 25 to 500 μM. Although all four ligands showed a concentration‐dependent increase in reactivity, acrylamides **1** and **6** had a roughly linear dependency up to a concentration of 500 μM, thus indicating that their *K*_i_ is likely greater than 1 mM and preventing calculation of *k*_inact_. Pleasingly, however, merged acrylamides **9** and **11** showed a clear hyperbolic relationship between concentration and Cdk2(ES) reactivity that facilitated the calculation of both *K*_i_ and *k*_inact_. Interestingly, both inhibitors have similar inactivation kinetics (*k*_inact_ (10^−3^ s^−1^): **9**=1.98; **11**=1.81) but the reversible binding of acrylamide **11** (*K*_i_=102 μM) is four times stronger than that of acrylamide **9** (*K*_i_=416 μM). This similarity in *k*_inact_ is consistent both with electronic arguments that the additional cyclopropane ring should have minimal effect on the electrophilicity of the warhead and with our structural studies, which show that the two acrylamides bind Cdk2(ES) in nearly identical conformations (Figure 7d). Indeed, comparing the binding conformations of the merged (**9** and **11**) and parent (**1** and **6**) fragments, we observe a subtle shift in the position of the core scaffolds and the orientation of the acrylamide link that likely explains the jump in potency that resulted from fragment merging. Overall, comparing acrylamides **9** and **11**, we observe in a 3.7‐fold increase in *k*_inact_/*K*_i_ and a 4.3‐fold increase in REF, and the consistency between these two orthogonal metrics of covalent fragment potency is encouraging for future applications of qIT to covalent fragment optimization.

In our initial report of qIT, we demonstrated how REF can be used as a screening metric to identify covalent fragment hits, even against cysteine residues in challenging pockets. Here, we have extended the utility of the qIT platform, demonstrating how its high‐throughput kinetic output can be re‐engineered from a primary screening assay into a tool for conducting comprehensive covSAR characterization during fragment optimization.

In applying qIT to discover covalent fragments for targeting electrophile sensitive kinases, we found that screening by REF analysis enabled potential false positives to be easily distinguished from true hits. Two hit compounds were identified from the initial screen (hit rate=1.5 %) that were extensively validated by mass spectrometry, X‐ray crystallography and biochemical kinase inhibition. Moreover, by accounting for its reduced intrinsic reactivity using REF analysis, we then identified acrylamide **6** as a hit fragment, despite reacting with Cdk2(ES) ten times more slowly than acrylamide **1**. We speculate that without quantitative incorporation of GSH reactivity into our analysis, this key compound that led to a successful fragment merging approach, would have been overlooked. Using this series of merged fragments, we demonstrate how combining multiparameter kinetic analysis by qIT with high‐resolution X‐ray crystallography yields insights into the complex relationship between covalent fragment structure, intrinsic reactivity, thermodynamic protein pre‐coordination and kinetic protein modification. We anticipate that these fragments may serve as starting points from which to develop more potent chemical probes for exploring Cdk2 biology using the electrophile sensitive approach. Indeed, the human kinome contains two members (MOK and SgK494) that have endogenous gatekeeper cysteine residues and the fragments reported here may aid in the development of inhibitors against these largely uncharacterized kinases.

## Supporting information

As a service to our authors and readers, this journal provides supporting information supplied by the authors. Such materials are peer reviewed and may be re‐organized for online delivery, but are not copy‐edited or typeset. Technical support issues arising from supporting information (other than missing files) should be addressed to the authors.

Supplementary
