# Supplementary material for: Multiparameter Kinetic Analysis for Covalent Fragment Optimization by Using Quantitative Irreversible Tethering (qIT)
Source: Chembiochem. 2020 Aug 7;21(23):3417–22. doi: 10.1002/cbic.202000457 (PMC7754465; doi:10.1002/cbic.202000457)
Supplement: Supplementary file 1 — Supplementary [file CBIC-21-3417-s001.pdf]

ChemBioChem

Supporting Information

Multiparameter Kinetic Analysis for Covalent Fragment Optimization by Using Quantitative Irreversible Tethering (qIT)

Gregory B. Craven, Dominic P. Affron, Teresa Kösel, Tsz Lam M. Wong, Zoë H. Jukes, Chun-Ting Liu, Rhodri M. L. Morgan, Alan Armstrong,* and David J. Mann*

Author Contributions

G.C. Conceptualization:Lead; Formal analysis:Lead; Investigation:Equal; Writing - Original Draft:Lead
D.A. Investigation:Equal
T.K. Investigation:Equal
T.W. Investigation:Supporting
Z.J. Investigation:Supporting
C.-T.L. Investigation:Supporting
R.M. Investigation:Supporting
A.A. Conceptualization:Equal; Supervision:Equal; Writing - Review & Editing:Equal
D.M. Conceptualization:Equal; Supervision:Equal; Writing - Review & Editing:Equal

Table of Contents

Supplementary Results 2
  Supplementary Figures 2
  Supplementary Tables 2

Supplementary Methods 7
  A. Chemical synthesis 7
  B. Synthesis and characterization of compounds 8
  C. Library design 54
  D. Rate determination by qIT 54
  E. Protein expression 54
  F. Protein purification 54
  G. Acrylamide labelling of Cdk2 for crystallography, kinase assays and intact-protein mass spectrometry 54
  H. Intact-protein mass spectrometry 55
  I. Crystallography 55
  J. Data collection and structure determination 55
  K. Cdk2 phosphorylation 55
  L. Kinase assay 55
  Q. Concentration dependent qIT 55

Supplementary Reference 56

Supplementary Results

Supplementary Figures

Figure S1: Crystal structure of Cdk2(ES)
Figure S2: Hit identification and validation for acrylamide **S1**
Figure S3: Mass spectrometric analysis of tryptic digests of labelled Cdk2(ES)
Figure S4: *In vitro* inhibition of Cdk2(ES) kinase activity
Figure S5: Crystal structure of **S1**-Cdk2(ES)
Figure S6: Crystal structure of **6**-Cdk2(ES)
Figure S7: Synthesis of merged acrylamide fragments **9**, **10** and **11**
Figure S8: Crystal structure of **9**-Cdk2(ES)
Figure S9: Crystal structure of **11**-Cdk2(ES)

Supplementary Tables

Table S1: Data collection and refinement statistics for novel crystal structures

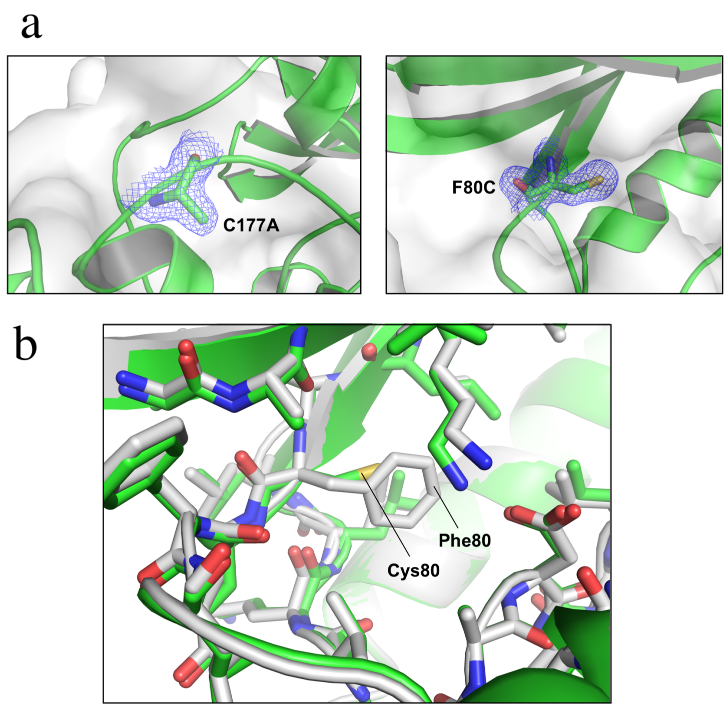

Supplementary Figure S1 | Crystal structure of Cdk2(ES) (PDB: 6YL6, resolution: 1.69 Å). (a) The $2F_o–F_c$ electron density map (blue) is contoured at 1σ around C177A and F80C. (b) Structure of the active site of Cdk2(F80C,C177A) (green) overlaid against Cdk2(WT) (grey).

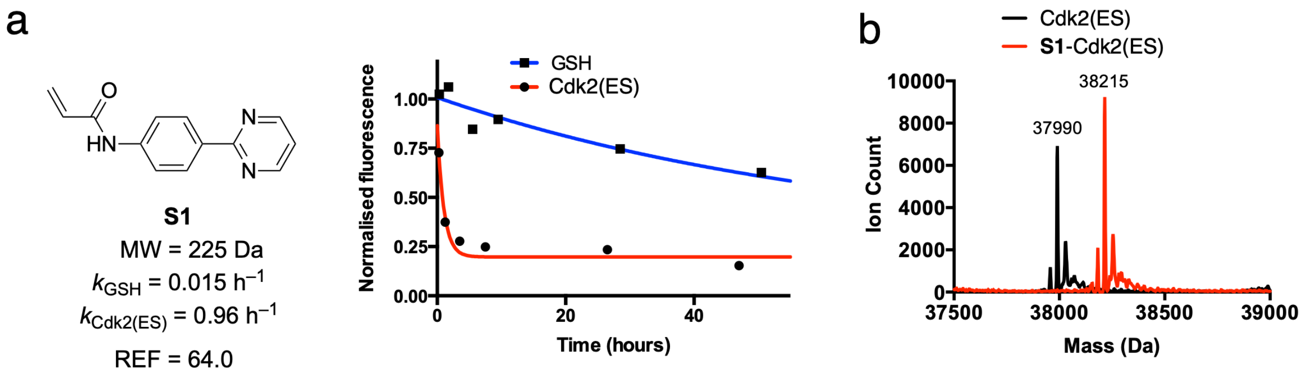

Supplementary Figure S2 | Hit identification and validation for acrylamide **S1**. (a) Fluorescence data (normalised against DMSO control) from qIT assay for hit-acrylamide **S1** (0.5 mM) in reaction with Cdk2(ES) and glutathione (5 µM). (b) Validation by LCMS. Spectra were recorded prior to and 5 hours after incubation of Cdk2(ES) (5 µM) with acrylamide **S1** (0.5 mM).

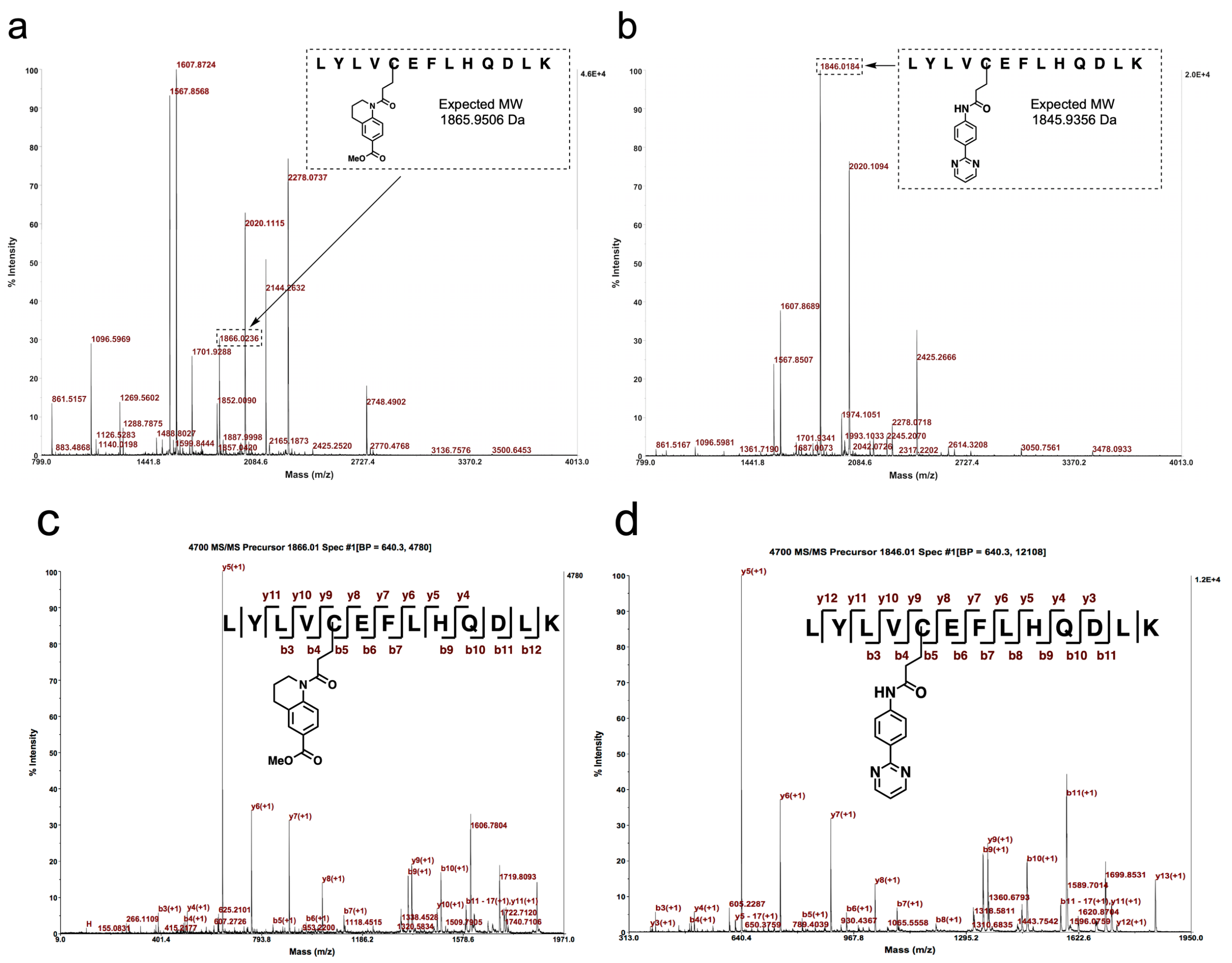

Supplementary Figure S3 | Mass spectrometric analysis of tryptic digests of labelled Cdk2(ES). MALDI-TOF spectra of the tryptic digests of (a) **1**-Cdk2(ES) conjugate and (b) **S1**-His-Cdk2(ES) conjugate. MALDI-TOF/TOF spectra of the labelled peptides. Precursor ions with (a) *m/z* = 1866 and (b) *m/z* = 1846.

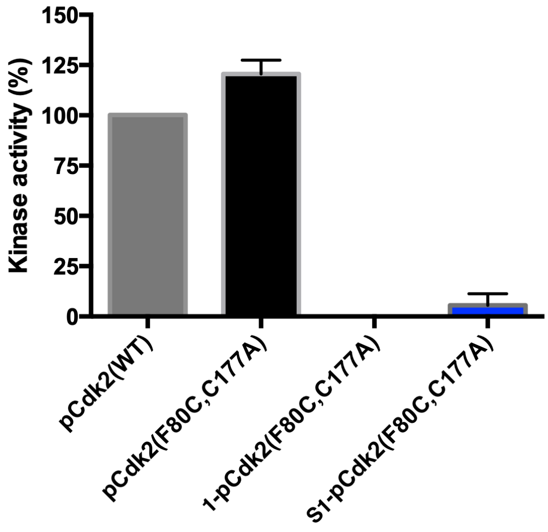

Supplementary Figure S4 | *In vitro* inhibition of Cdk2(ES) kinase activity. WT, ES or modified pCdk2 was incubated with cyclin A2 to form active holoenzymes. The holoenzymes were incubated with peptide substrate, ATP, NADH, PEP, LD and PK at 37 ˚C and the absorbance measured over time in clear 384-well plates. Kinase activity is determined from the gradient of the absorbance over time (between 1000–3000 s) and normalised relative to pCdk2(WT) = 100% and cyclin A2 only = 0% (n = 4; error bars denote s.e.m).

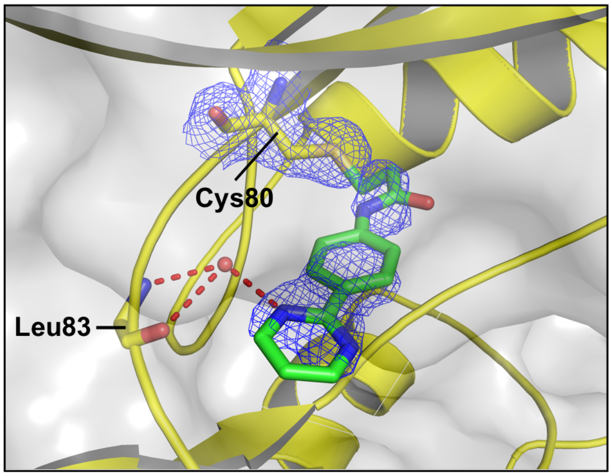

Supplementary Figure S5 | Crystal structure of **S1**-Cdk2(ES) (resolution: 2.0 Å, PDB ID: 5OO1). The 2F$_o$–F$_c$ electron density map (blue) is contoured at 1σ around Cys80 (cyan) and the ligand (green).

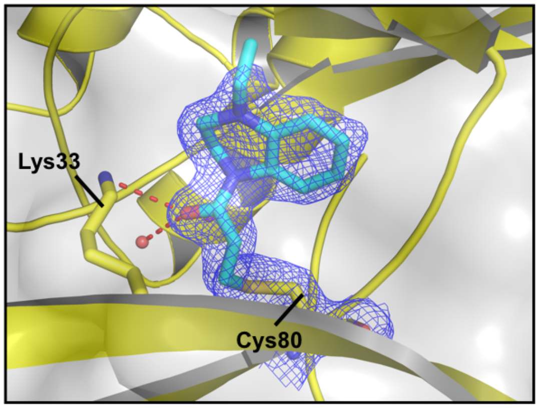

Supplementary Figure S6 | Crystal structure of **6**-Cdk2(ES) (resolution 1.72 Å, PDB: 5OO3). The 2F$_o$–F$_c$ electron density map (blue) is contoured at 1σ around Cys80 (yellow) and the ligand (cyan), with the hydrogen bonds to K33 and water molecules shown in red.

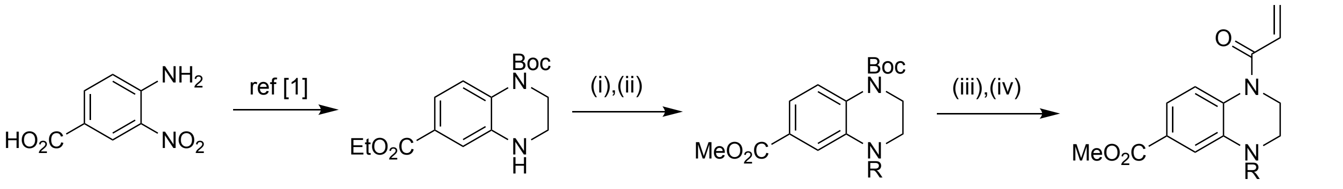

S-G

S-I (R = Et)
S-K (R = Me)
S-M (R = $CH_2CHCH_2CH_2$)

9 (R = Et)
10 (R = Me)
11 (R = $CH_2CHCH_2CH_2$)

Supplementary Figure S7 | Synthesis of merged acrylamide fragments **9**, **10** and **11**. Intermediate **S-G** was synthesized according to Ackermann's protocol.[1] (i) RCHO, NaBH(OAc)$_3$, AcOH, CH_2Cl_2, rt. (ii) K_2CO_3, MeOH, rt. (iii) HCl/dioxane, rt. (iv) acryloyl chloride, NEt_3, $CHCl_3$, rt.

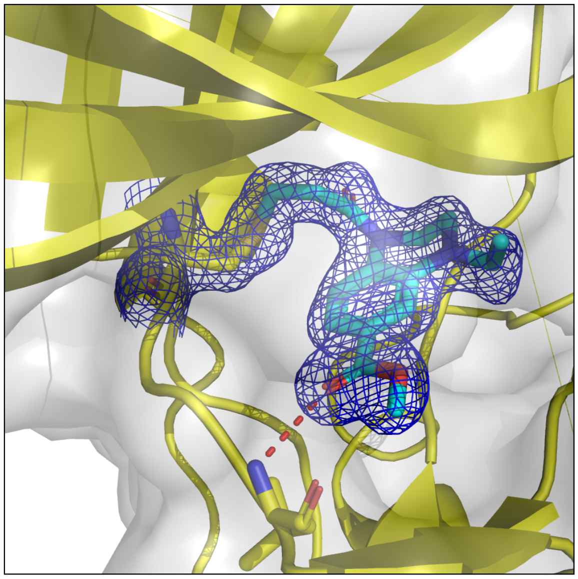

Supplementary Figure S8 | Crystal structure of **9**-Cdk2(ES) (resolution: 1.65 Å, PDB ID: 6YLK). The $2F_o–F_c$ electron density map (blue) is contoured at 1σ around Cys80 (yellow) and the ligand (cyan), with the hydrogen bond to L83 shown in red.

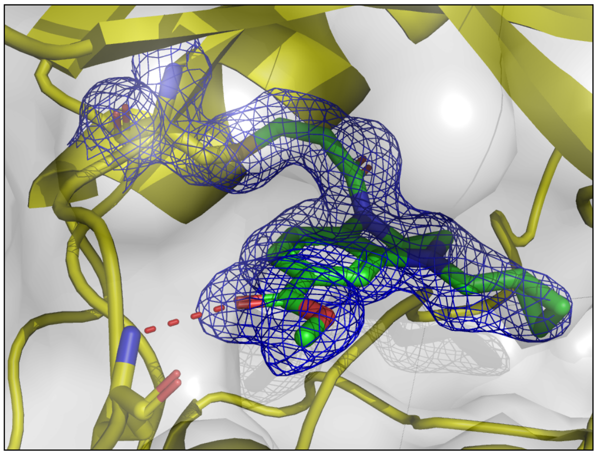

Supplementary Figure S9 | Crystal structure of **11**-Cdk2(ES) (resolution: 1.66 Å, PDB ID: 6YL1). The $2F_o–F_c$ electron density map (blue) is contoured at 1σ around Cys80 (yellow) and the ligand (green), with the hydrogen bond to L83 shown in red.

Table S1. Data collection and refinement statistics for novel crystal structures

	Cdk2(F80C,C177A)	**1**-Cdk2(F80C,C177A)	**6**-Cdk2(F80C,C177A)	**9**-Cdk2(F80C,C177A)	**11**-Cdk2(F80C,C177A)	**S1**-Cdk2(F80C,C177A)
PDB Code	6YL6	5OSM	5OO3	6YLK	6YL1	5OO1
Wavelength	0.9763	0.9763	0.9763	0.9763	0.9763	0.9763
Resolution range	42.41 - 1.7 (1.761 - 1.7)	42.37 - 1.77 (1.833 - 1.77)	36.67 - 1.73 (1.792 - 1.73)	42.85 - 1.65 (1.709 - 1.65)	36.71 - 1.66 (1.719 - 1.66)	48.22 - 2.0 (2.071 - 2.0)
Space group	P 21 21 21	P 21 21 21	P 21 21 21	P 21 21 21	P 21 21 21	P 21 21 21
Unit cell	52.65 71.36 71.56 90 90 90	52.71 71.22 72.32 90 90 90	53.11 70.97 72.43 90 90 90	53.38 71.10 71.85 90 90 90	53.34 71.24 71.87 90 90 90	49.58 66.49 70.05 90 90 90
Total reflections	59882 (5289)	54367 (5297)	58218 (5666)	66260 (6557)	65258 (6457)	31762 (2998)
Unique reflections	29979 (2672)	27186 (2650)	29150 (2837)	33479 (3308)	32900 (3244)	15979 (1519)
Multiplicity	2.0 (2.0)	2.0 (2.0)	2.0 (2.0)	2.0 (2.0)	2.0 (2.0)	2.0 (2.0)
Completeness (%)	98.88 (90.88)	99.96 (99.89)	99.69 (99.54)	99.63 (99.67)	99.57 (99.72)	98.54 (95.83)
Mean I/sigma(I)	16.92 (2.22)	32.76 (13.20)	15.73 (1.28)	6.36 (0.93)	7.81 (1.23)	17.97 (5.53)
Wilson B-factor	24.24	17.23	26.42	28.1	24.6	34.25
R-meas	0.02338 (0.3965)	0.01776 (0.6058)	0.04147 (0.8468)	0.07614 (0.8495)	0.08795 (0.9638)	0.02173 (0.1455)
R-pim	0.01653 (0.2804)	0.01256 (0.4284)	0.02933 (0.5988)	0.05384 (0.6007)	0.06219 (0.6815)	0.01536 (0.1029)
CC1/2	1 (0.782)	1 (0.994)	0.999 (0.618)	0.996 (0.49)	0.995 (0.488)	1 (0.985)
CC*	1 (0.937)	1 (0.998)	1 (0.874)	0.999 (0.811)	0.999 (0.81)	1 (0.996)
Reflections used in refinement	29977 (2671)	27183 (2650)	29148 (2837)	33476 (3307)	32893 (3244)	15968 (1517)
Reflections used for R-free	1539 (124)	1381 (140)	1404 (142)	1741 (173)	1720 (193)	746 (56)
R-work	0.1815 (0.2417)	0.1607 (0.1683)	0.1860 (0.3530)	0.2160 (0.3868)	0.2212 (0.3756)	0.1817 (0.2262)
R-free	0.2117 (0.3200)	0.2057 (0.2490)	0.2499 (0.4201)	0.2650 (0.4062)	0.2685 (0.4327)	0.2368 (0.2746)
CC(work)	0.965 (0.841)	0.963 (0.914)	0.953 (0.457)	0.954 (0.777)	0.949 (0.787)	0.939 (0.500)
CC(free)	0.961 (0.623)	0.968 (0.938)	0.915 (0.400)	0.943 (0.788)	0.937 (0.767)	0.918 (0.826)
Number of non-hydrogen atoms	2642	2671	2585	2461	2409	2261
macromolecules	2425	2321	2293	2323	2268	2172
ligands		18	16	20	22	17
solvent	217	332	276	118	119	72
Protein residues	303	292	286	295	287	278
RMS(bonds)	0.006	0.023	0.019	0.007	0.007	0.018
RMS(angles)	0.87	2.2	1.87	0.95	0.97	1.9
Ramachandran favored (%)	98.67	97.57	98.23	98.28	97.86	97.81
Ramachandran allowed (%)	1.33	2.43	1.77	1.72	2.14	2.19
Ramachandran outliers (%)	0	0	0	0	0	0
Rotamer outliers (%)	0.76	0.8	0.8	0	0.41	1.74
Clashscore	3.06	2.56	6.03	5.58	3.94	3.68
Average B-factor	29.7	22.33	33.39	39.28	35.84	40.92
macromolecules	29.26	20.89	32.53	39.13	35.82	40.59
ligands		26.33	30.87	32.16	34.07	69.41
solvent	34.62	32.21	40.72	43.39	36.65	44.29

Statistics for the highest-resolution shell are shown in parentheses.

Supplementary Methods

A. Chemical synthesis

All non-aqueous reactions were carried out under an inert atmosphere (argon) with flame-dried glassware, using standard techniques. Anhydrous solvents were obtained by filtration through drying columns (DMF, CH_2Cl_2, THF).

Flash column chromatography was performed using 230-400 mesh silica, with the indicated solvent system according to standard techniques. Analytical thin-layer chromatography (TLC) was performed on precoated aluminium-backed silica gel plates. Visualisation of the developed chromatogram was performed by UV absorbance (254 nm) and/or stained with aqueous potassium permanganate solution, aqueous ceric ammonium molybdate, or a ninhydrin solution in ethanol.

Nuclear magnetic resonance spectra were recorded on 400 MHz or 500 MHz spectrometers. Chemical shifts for ^{1}H NMR spectra are recorded in parts per million from tetramethylsilane with the residual protic solvent resonance as the internal standard (chloroform: δ 7.27 ppm, methanol: δ 3.31 ppm). Data are reported as follows: chemical shift (multiplicity [s = singlet, d = doublet, t = triplet, m = multiplet and br = broad], coupling constant (in Hz), integration). ^{13}C NMR spectra are recorded with complete proton decoupling. Chemical shifts are reported in parts per million from tetramethylsilane with the solvent resonance as the internal standard ($^{13}CDCl_3$: δ 77.0 ppm, $^{13}CD_3OD$: δ 49.0 ppm). Assignments of ^{1}H and ^{13}C spectra were based upon the analysis of δ and J values, as well as DEPT, COSY, HMBC, HSQC and nOe experiments where appropriate.

Commercial reagents were used as supplied or purified by standard techniques where necessary. Compounds **1** and **2** were used as commercially supplied.

B. Synthesis and characterization of compounds

N-(4-(Pyrimidin-2-yl)phenyl)acrylamide (S1)

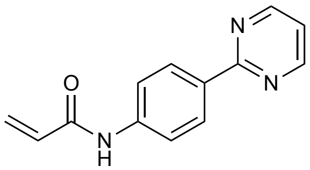

Acrylic acid (68 μL, 1.00 mmol) and 2-(4'-aminophenyl)pyrimidine hydrochloride (25 mg, 0.12 mmol) were added to a solution of polystyrene-supported 2-isobutoxy-1-isobutoxycarbonyl-1,2-dihydroquinoline (133 mg, 0.20 mmol) in MeCN (3.0 mL), and the reaction was shaken for 24 h at rt. The mixture was then filtered through Celite, washed with CH_2Cl_2:MeOH (1:1, 10 mL), and the filtrate was concentrated under reduced pressure. CH_2Cl_2 (5 mL) and sat. aq. $NaHCO_3$ (5 mL) were added and the phases were separated. The aqueous layer was extracted with CH_2Cl_2 (3 × 5 mL), and the combined organic layers were dried over $MgSO_4$. The solvent was removed under reduced pressure and the crude residue was purified by flash column chromatography (75% EtOAc/hexane), which afforded acrylamide **S1** (8 mg, 36%) as a white solid.

The compound was also made through a modification of our previously reported procedure.[2] Acryloyl chloride (9.8 μL, 0.12 mmol) in CH_2Cl_2 (80 μL) was added to a solution of 2-(4'-aminophenyl)pyrimidine hydrochloride (21 mg, 0.10 mmol) in $CHCl_3$ (0.60 mL) at rt. After 10 min, Amberlyst A26(OH) resin (27 mg) was added, and the resulting suspension was stirred at rt for 1 h. The reaction mixture was then filtered through Celite washing with MeOH (10 mL) and concentrated under reduced pressure. The crude residue was purified by flash column chromatography (25% grading to 40% EtOAc/toluene), which afforded acrylamide **S1** (8 mg, 37%) as a white solid.

m.p. = 203–206 ºC (MeOH)

R_f 0.38 (50% EtOAc/toluene);

ν_{max} (film)/cm^{-1} 3309, 2925, 2427, 2389, 1662, 1606, 1566, 1537, 1430, 1408, 1176, 794;

^{1}H NMR (500 MHz, CD_3OD) δ 8.81 (d, *J* = 4.9 Hz, 2 H), 8.40–8.35 (m, 2 H), 7.81–7.77 (m, 2 H), 7.32 (t, *J* = 4.9 Hz, 1 H), 6.47 (dd, *J* = 17.0, 9.8 Hz, 1 H), 6.40 (dd, *J* = 17.0, 2.0 Hz, 1 H), 5.80 (dd, *J* = 9.8, 2.0 Hz, 1 H);

^{13}C NMR (126 MHz, CD_3OD) δ 166.2, 165.3, 158.7 (2 × C_{Ar}), 142.4, 134.4, 132.4, 129.9 (2 × C_{Ar}), 128.2, 120.9 (2 × C_{Ar}), 120.4;

HRMS (CI$^+$) *m/z* Calculated for $C_{13}H_{12}N_3O^+$ [M+H]$^+$ 226.0980; Found 226.0977 (D –1.3 ppm).

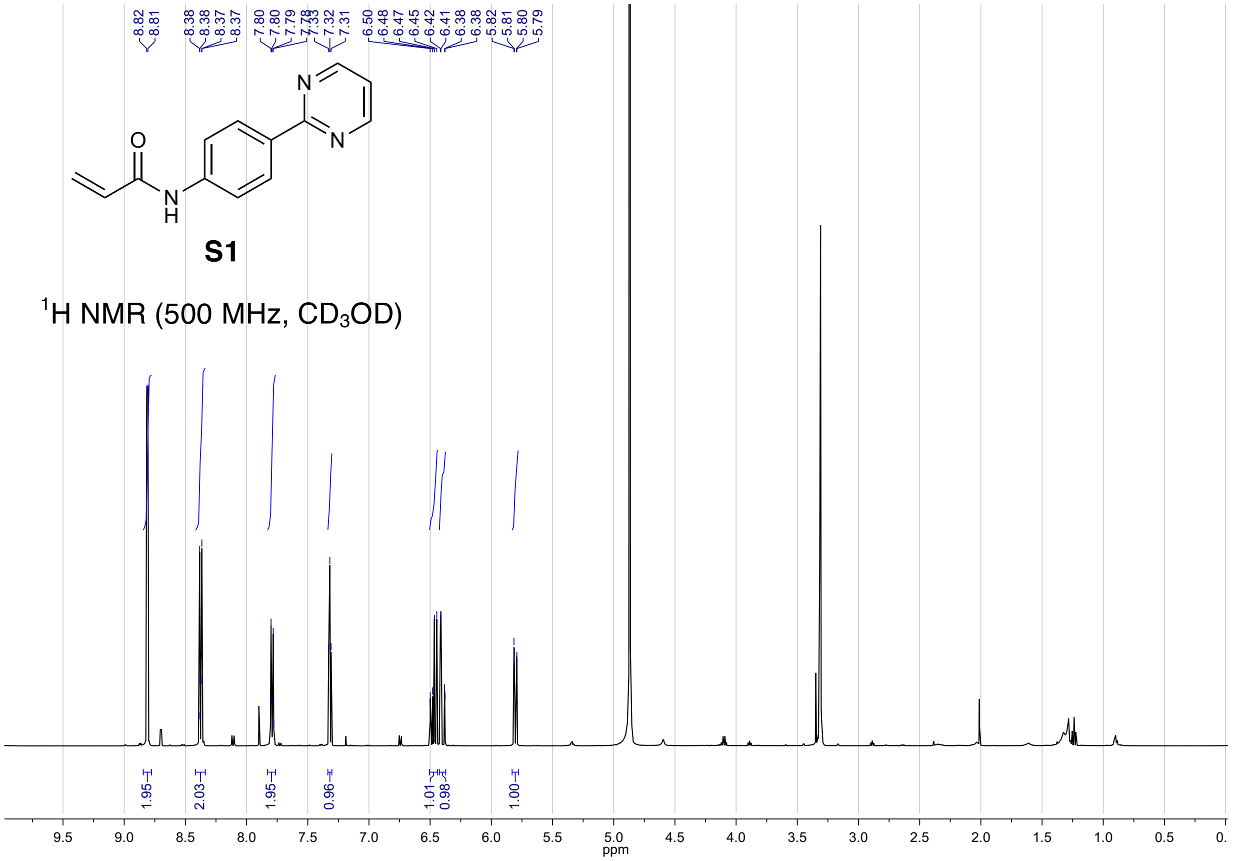
8.82
8.81
8.38
8.38
8.37
8.37
7.80
7.80
7.79
7.78
7.33
7.32
7.31
6.50
6.48
6.47
6.45
6.42
6.41
6.38
6.38
5.82
5.81
5.80
5.79
O
N
H
N
N
S1
^{1}H NMR (500 MHz, CD_3OD)
1.95
2.03
1.95
0.96
1.01
0.98
1.00
9.5
9.0
8.5
8.0
7.5
7.0
6.5
6.0
5.5
5.0
ppm
4.5
4.0
3.5
3.0
2.5
2.0
1.5
1.0
0.5

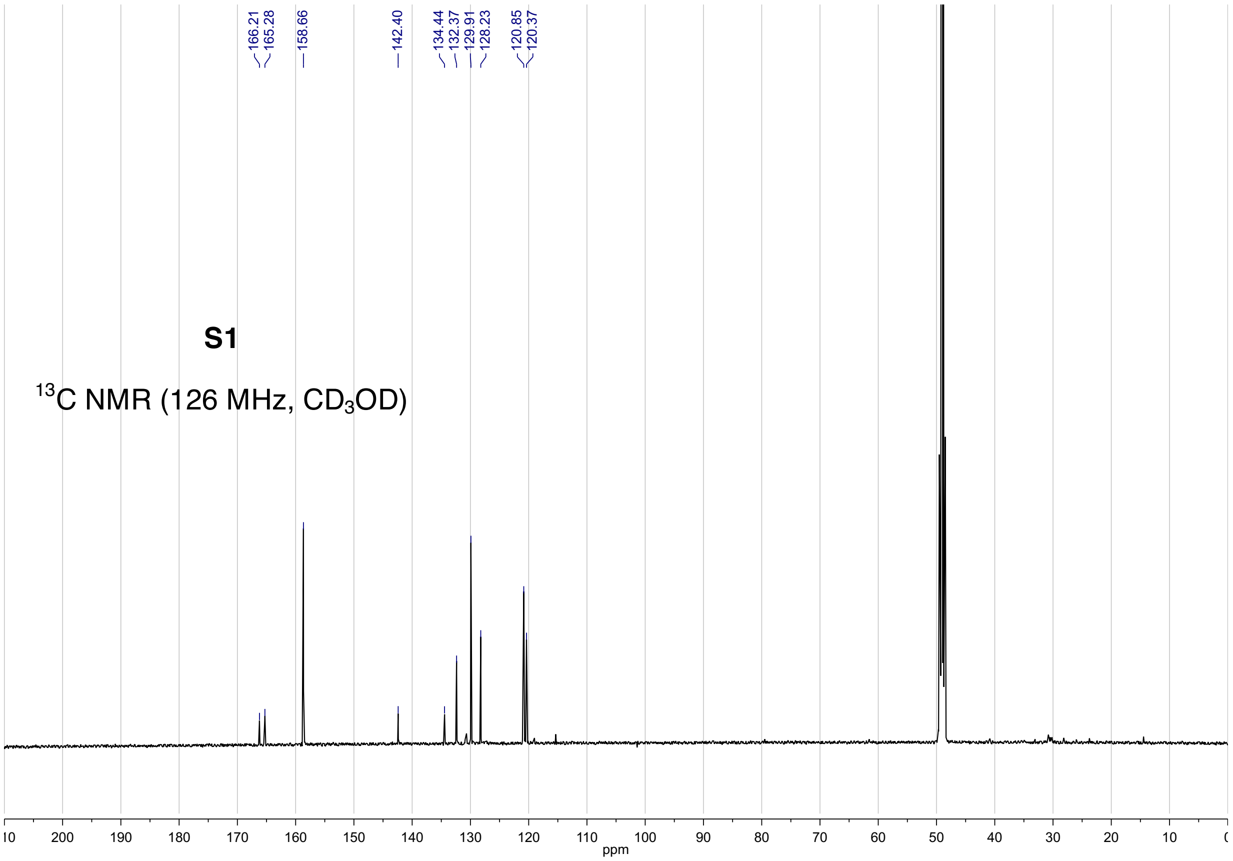
166.21
165.28
158.66
142.40
134.44
132.37
129.91
128.23
120.85
120.37
S1
^{13}C NMR (126 MHz, CD_3OD)
200
190
180
170
160
150
140
130
120
110
ppm
100
90
80
70
60
50
40
30
20
10

1-(3,4-Dihydroquinoxalin-1(2*H*)-yl)-2-(phenylselanyl)propan-1-one (S-A)

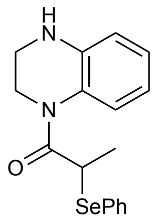

Oxalyl chloride (254 µL, 3.00 mmol) was added dropwise to a solution of 2-(phenylselanyl)propanoic acid (687 mg, 3.00 mmol) in THF (10 mL) at 0 ºC. DMF (2 drops) was added and the reaction mixture was allowed to warm to rt for 2 h. This solution was then added dropwise to a solution of NEt_3 (627 µL, 4.50 mmol) and 1,2,3,4-tetrahydroquinoxaline (403 mg, 3.00 mmol) in THF (10 mL). The reaction mixture was allowed to stir at rt for 18 h. Saturated aqueous $NaHCO_3$ (50 mL) was added, and the resulting mixture was extracted with CH_2Cl_2 (3 × 50 mL). The organic layers were combined, dried over Na_2SO_4, filtered, and the solvent was removed under reduced pressure. The resulting crude material was purified by flash column chromatography (10% grading to 30% EtOAc/pentane), which afforded 1-(3,4-dihydroquinoxalin-1(2*H*)-yl)-2-(phenylselanyl)propan-1-one **S-A** (788 mg, 76%) as a white solid.

m.p = 127–129 ºC ($CHCl_3$)

R_f 0.33 (25% EtOAc/pentane);

ν_{max} (film)/cm^{-1} 3356, 3056, 2939, 2863, 1633, 1603, 1503, 1391, 1323, 738;

^{1}H NMR (400 MHz, $CDCl_3$) δ 7.41–7.32 (m, 2 H), 7.30–7.24 (m, 1 H), 7.23–7.15 (m, 2 H), 7.07–6.97 (m, 2 H), 6.65–6.56 (m, 2 H), 4.71–4.58 (m, 1 H), 4.32–4.19 (m, 1 H), 4.15–4.01 (m, 1 H), 3.55–3.25 (m, 3 H), 1.69 (d, *J* = 6.9 Hz, 3 H);

^{13}C NMR (101 MHz, $CDCl_3$) δ 172.6, 138.3, 134.9 (2 × C_{Ar}), 128.9 (2 × C_{Ar}), 128.4, 127.9, 126.8, 124.4, 124.1, 116.1, 114.6, 42.1, 39.2, 36.7, 19.0;

HRMS (ESI$^+$) *m/z* Calculated for $C_{17}H_{19}N_2O^{80}Se^+$ $[M+H]^+$ 347.0663; Found 347.0679 (Δ +4.6 ppm).

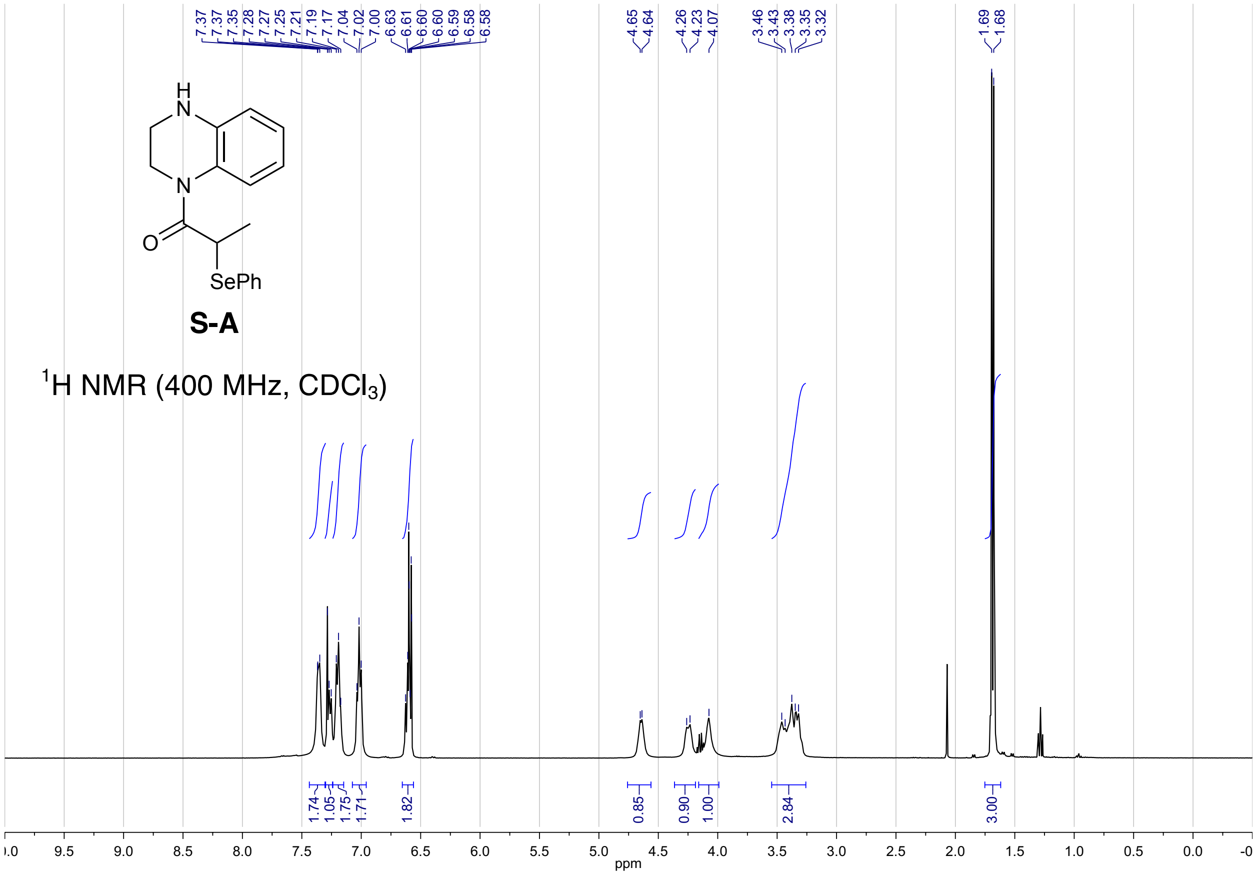

S-A
^{1}H NMR (400 MHz, CDCl$_3$)
7.37 7.37 7.35 7.28 7.27 7.25 7.21 7.19 7.17 7.04 7.02 7.00 6.63 6.61 6.60 6.60 6.59 6.58 6.58
4.65 4.64 4.26 4.23 4.07 3.46 3.43 3.38 3.35 3.32 1.69 1.68
1.74 1.05 1.75 1.71 1.82 0.85 0.90 1.00 2.84 3.00
ppm

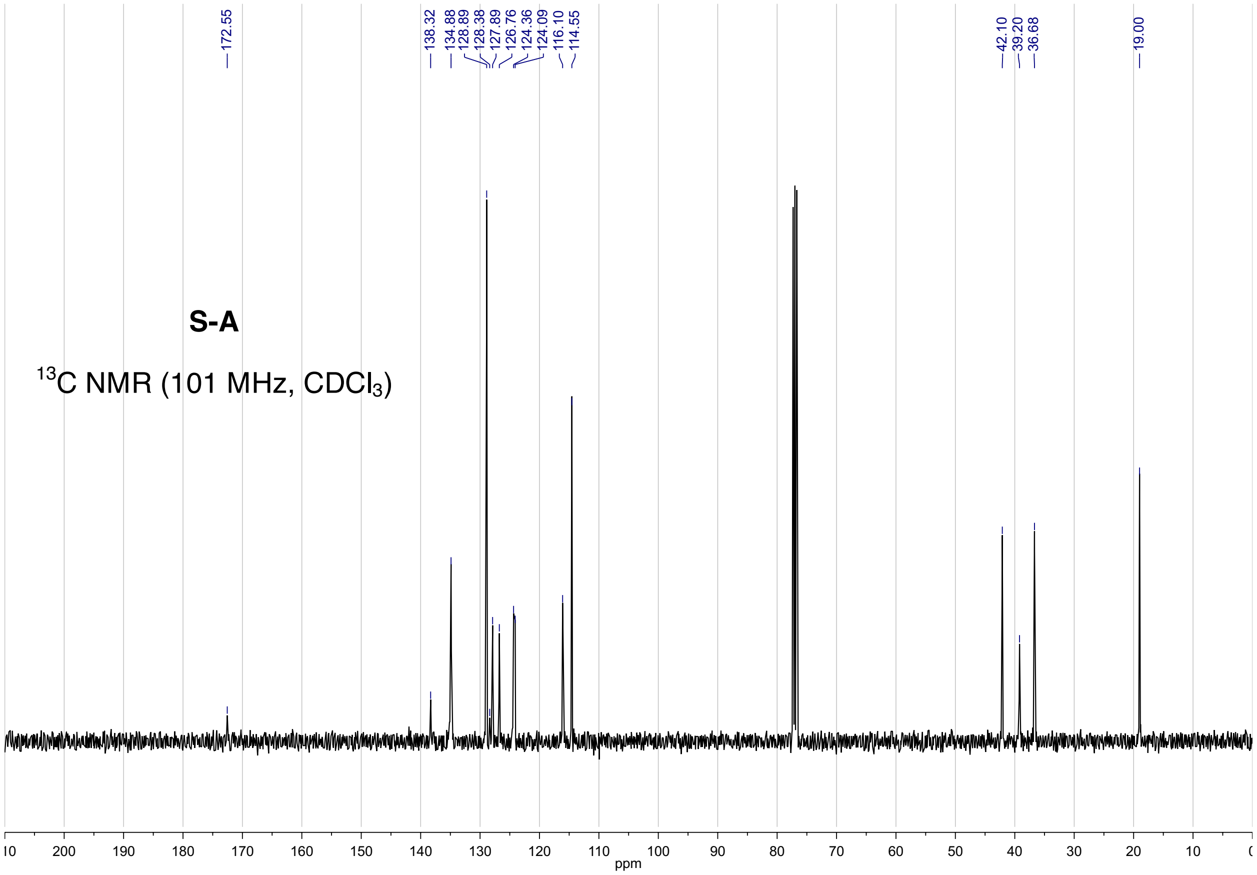

S-A
^{13}C NMR (101 MHz, CDCl$_3$)
172.55
138.32 134.88 128.89 128.38 127.89 126.76 124.36 124.09 116.10 114.55
42.10 39.20 36.68
19.00
ppm

Methyl 1-(2-(phenylselanyl)propanoyl)-1,2,3,4-tetrahydroquinoline-6-carboxylate (S-B)

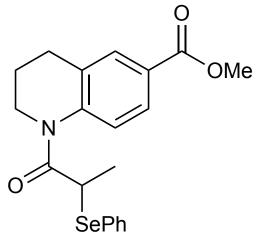

Oxalyl chloride (254 μL, 3.00 mmol) was added dropwise to a solution of 2-(phenylselanyl)propanoic acid (687 mg, 3.00 mmol) in THF (10 mL) at 0 ºC. DMF (2 drops) was added and the reaction mixture was allowed to warm to rt for 2 h. This solution was then added dropwise to a solution of NEt_3 (627 μL, 4.50 mmol) and methyl 1,2,3,4-tetrahydroquinoline-6-carboxylate (574 mg, 3.00 mmol) in THF (10 mL). The reaction mixture was allowed to stir at rt for 18 h. Sat. aq. $NaHCO_3$ (50 mL) was added, and the resulting mixture was extracted with CH_2Cl_2 (3 × 50 mL). The organic layers were combined, dried over Na_2SO_4, filtered, and the solvent was removed under reduced pressure. The resulting crude material was purified by flash column chromatography (5% grading to 15% EtOAc/pentane), which afforded methyl 1-(2-(phenylselanyl)propanoyl)-1,2,3,4-tetrahydroquinoline-6-carboxylate **S-B** (812 mg, 67%) as a colourless oil.

R_f 0.45 (25% EtOAc/pentane);

ν_{max} (film)/cm^{-1} 2949, 1716, 1654, 1608, 1578, 1497, 1437, 1373, 1276, 1228, 1192, 771, 740, 692;

^{1}H NMR (400 MHz, $CDCl_3$) δ 7.84–7.61 (m, 2 H), 7.43–7.14 (m, 6 H), 4.50–4.38 (m, 1 H), 4.09–3.95 (m, 1 H), 3.92 (s, 3 H), 3.58–3.46 (m, 1 H), 2.75–2.64 (m, 1 H), 2.61–2.46 (m, 1 H), 2.02–1.90 (m, 1 H), 1.89–1.76 (m, 1 H), 1.73–1.54 (m, 3 H);

^{13}C NMR (101 MHz, $CDCl_3$) δ 173.1, 166.6, 143.1, 135.2 (2 × C_{Ar}), 130.1, 129.1, 129.0 (2 × C_{Ar}), 128.3, 127.6, 126.7, 124.0, 110.0, 52.2, 44.0, 37.1, 26.7, 23.6, 19.0;

HRMS (ESI$^+$) *m/z* Calculated for $C_{20}H_{22}NO_3{}^{80}Se^+$ $[M+H]^+$ 404.0765; Found 404.0780 (Δ +3.7 ppm).

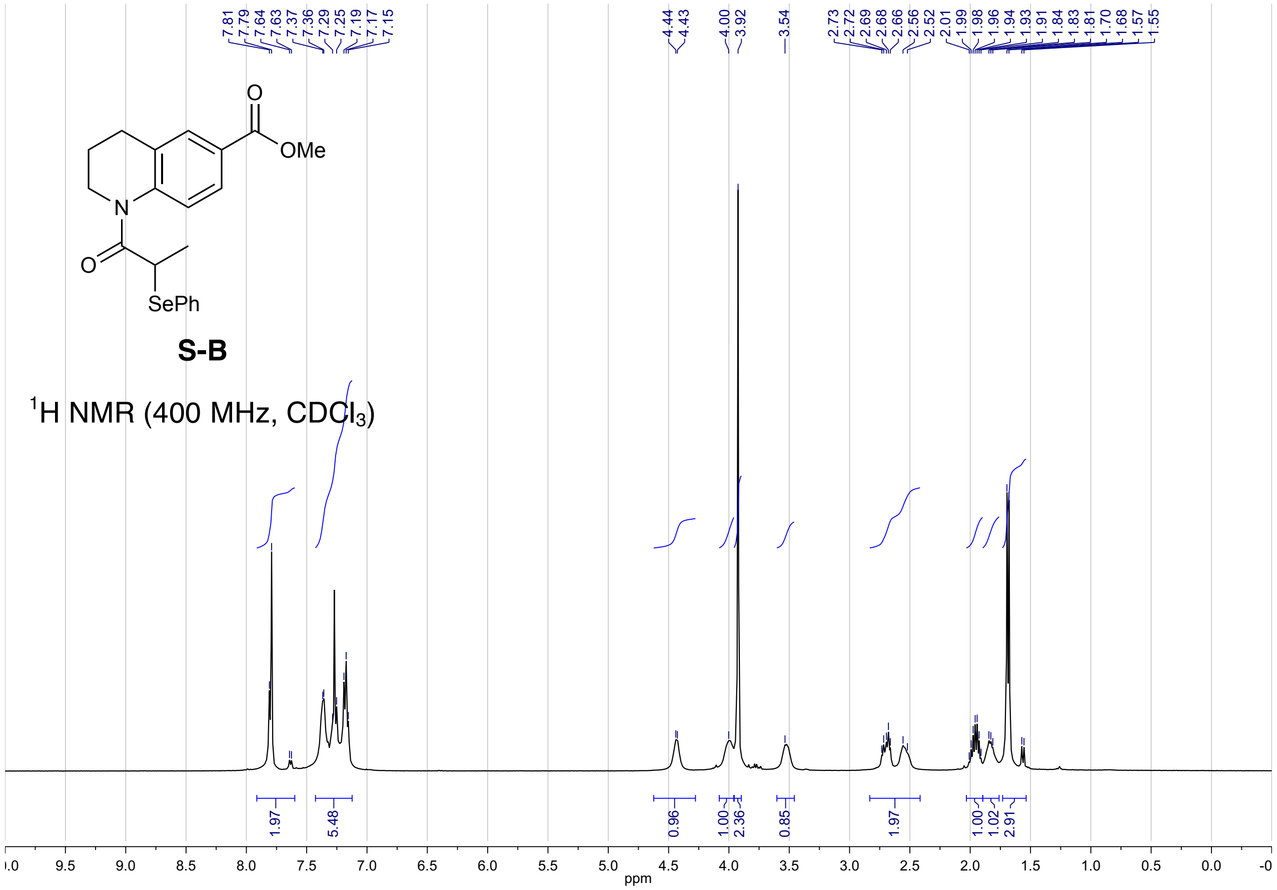

7.81 7.79 7.64 7.63 7.37 7.36 7.29 7.25 7.19 7.17 7.15
4.44 4.43
4.00 3.92
3.54
2.73 2.72 2.69 2.68 2.66 2.56 2.52 2.01 1.99 1.98 1.96 1.94 1.93 1.91 1.84 1.83 1.81 1.70 1.68 1.57 1.55
O
OMe
N
O
SePh
S-B
1H NMR (400 MHz, CDCl3)
1.97
5.48
0.96
1.00
2.36
0.85
1.97
1.00
1.02
2.91
ppm

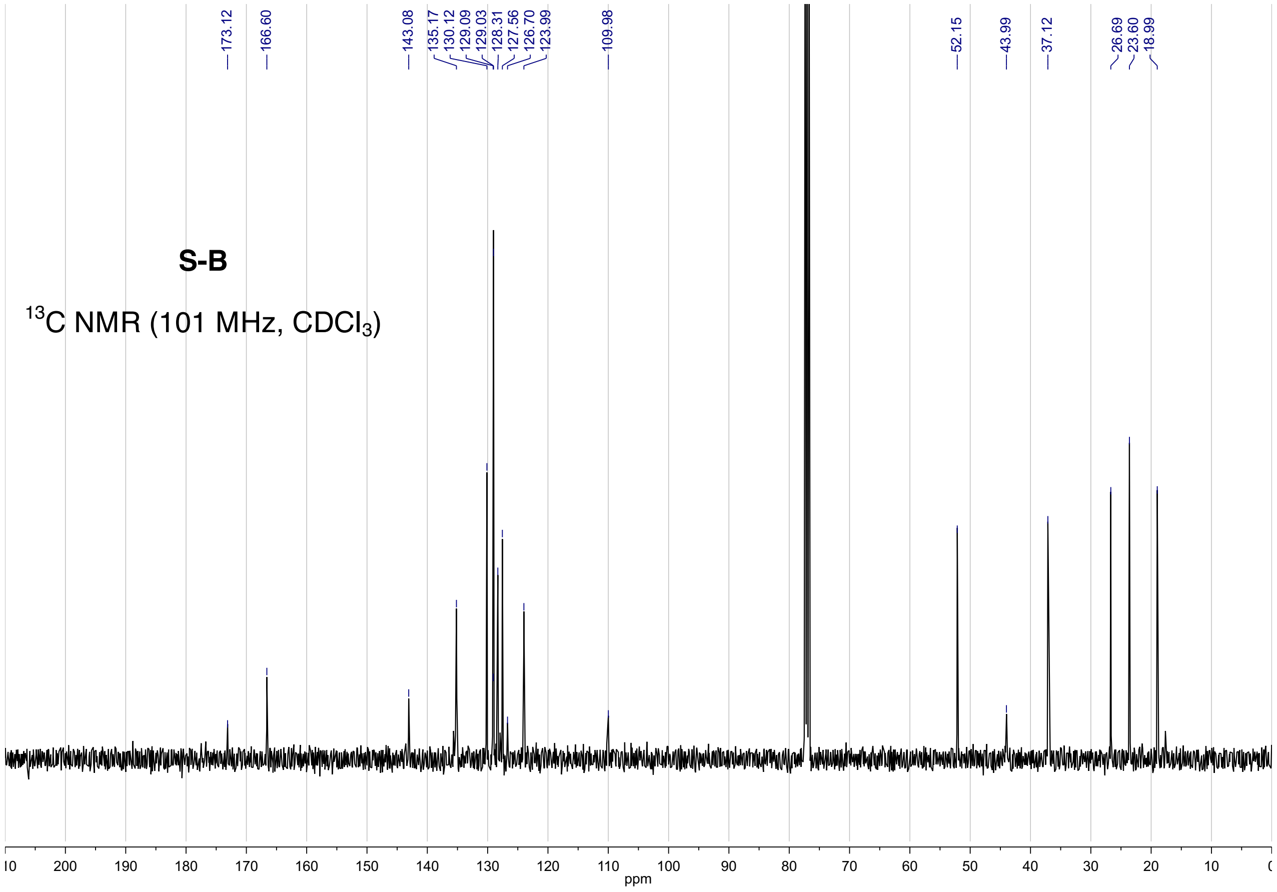

173.12
166.60
143.08
135.17
130.12
129.09
129.03
128.31
127.56
126.70
123.99
109.98
52.15
43.99
37.12
26.69
23.60
18.99
S-B
13C NMR (101 MHz, CDCl3)
ppm

1-(2-(Phenylselanyl)propanoyl)-1,2,3,4-tetrahydroquinoline-6-carboxylic acid (S-C)

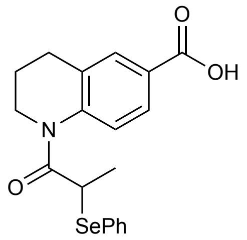

To a solution of methyl 1-(2-(phenylselanyl)propanoyl)-1,2,3,4-tetrahydroquinoline-6-carboxylate **S-B** (344 mg, 0.85 mmol) in H_2O (5 mL) and MeOH (11 mL) was added 1.0 M aq. NaOH (2.56 mL, 2.56 mmol). After 18 h, the MeOH was removed under reduced pressure and the resulting aqueous solution was washed with CH_2Cl_2 (2 × 10 mL). The aqueous solution was then acidified with 1.0 M aq. HCl (10 mL) and extracted with EtOAc (2 × 10 mL). The combined EtOAc fractions were washed with brine (2 × 10 mL), dried over Na_2SO_4, filtered and concentrated under reduced pressure to give 1-(2-(phenylselanyl)propanoyl)-1,2,3,4-tetrahydroquinoline-6-carboxylic acid **S-C** (299 mg, 90%) as a white crystalline solid (>90% purity by ^{1}H NMR spectroscopy) and was used in the next step without further purification.

m.p. = 91–94 ºC (CH_2Cl_2)

R_f 0.20 (3% MeOH/CH_2Cl_2)

ν_{max} (thin film)/cm^{-1} 2954, 2551, 1685, 1652, 1604, 1437, 1374, 1279, 1167, 733;

^{1}H NMR (400 MHz, $CDCl_3$) δ 7.91–7.85 (m, 2 H), 7.42–7.35 (m, 2 H), 7.32–7.27 (m, 2 H), 7.23–7.16 (m, 2 H), 4.50–4.40 (m, 1 H), 4.09–3.97 (m, 1 H), 3.61–3.50 (m, 1 H), 2.73 (dt, *J* = 15.4, 6.4 Hz, 1 H), 2.63–2.51 (m, 1 H), 2.03–1.93 (m, 1 H), 1.91–1.81 (m, 1 H), 1.74–1.68 (m, 3 H);

^{13}C NMR (101 MHz, $CDCl_3$) δ 173.2, 171.3, 143.8, 135.2 (2 × C_{Ar}), 130.7, 129.1, 129.0 (2 × C_{Ar}), 128.4, 128.2, 127.9, 125.7, 124.1, 44.1, 37.1, 26.7, 23.5, 18.9.

HRMS (ESI$^+$) *m/z* Calculated for $C_{19}H_{20}NO_3{}^{80}Se^+$ $[M+H]^+$ 390.0608, Found 390.0611 (Δ +0.8 ppm).

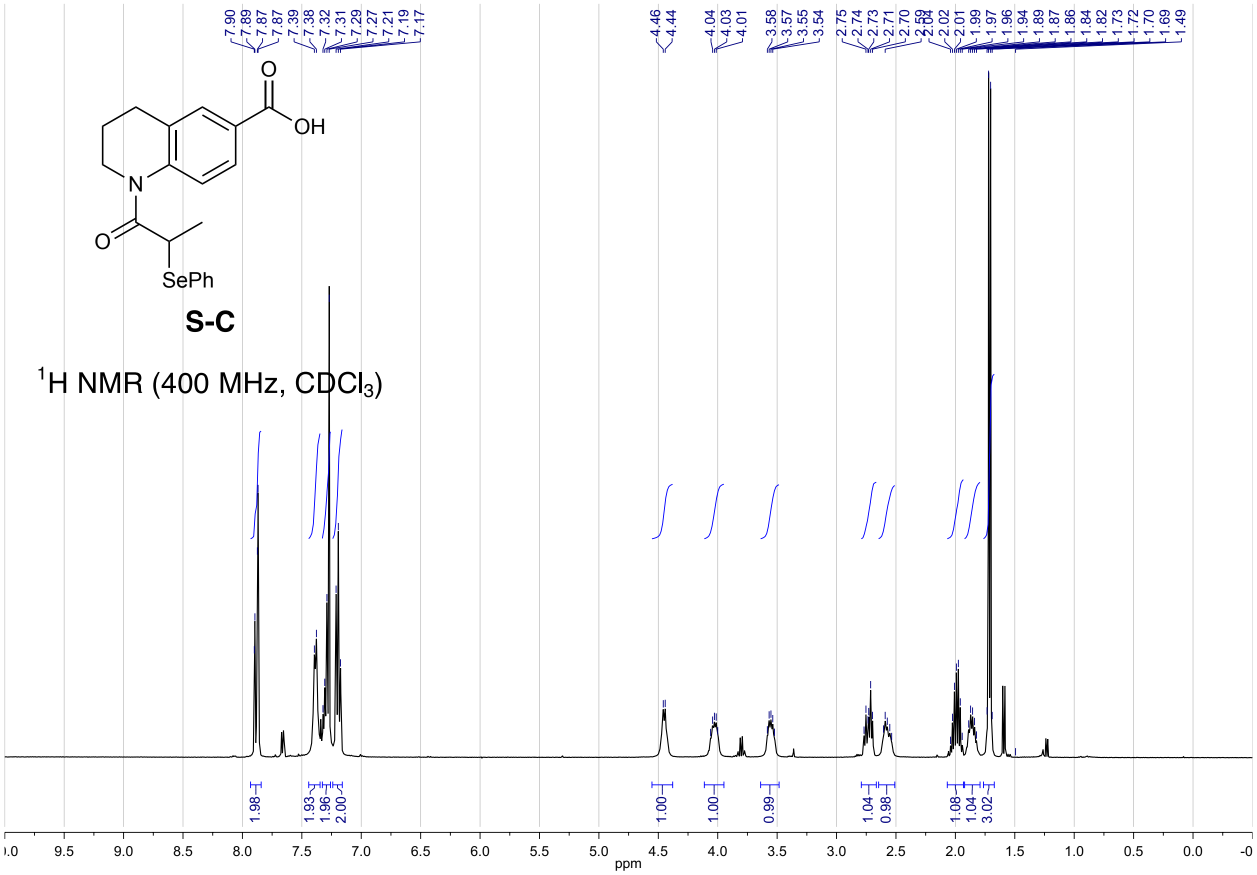
S-C
^{1}H NMR (400 MHz, CDCl$_3$)
ppm

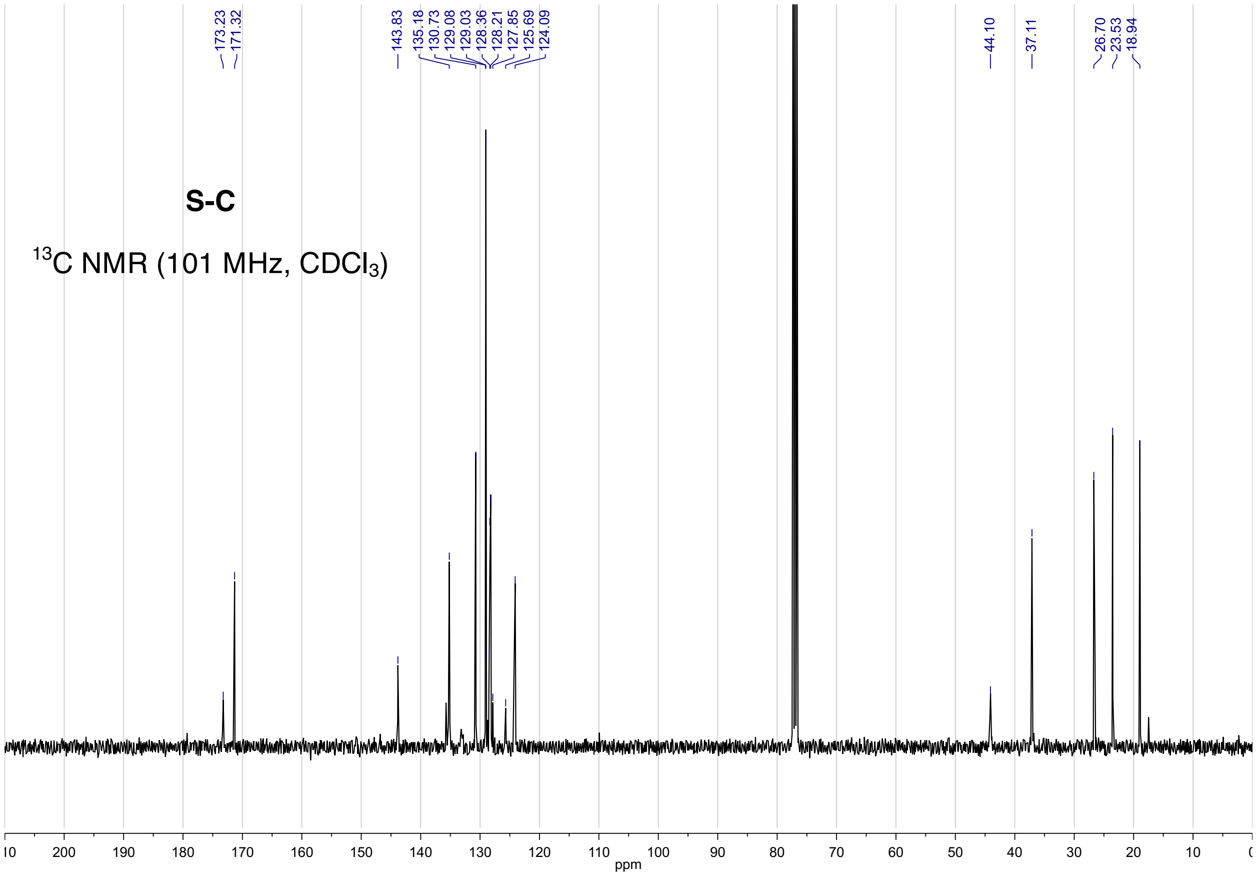
S-C
^{13}C NMR (101 MHz, CDCl$_3$)
173.23
171.32
143.83
135.18
130.73
129.08
129.03
128.36
128.21
127.85
125.69
124.09
44.10
37.11
26.70
23.53
18.94
ppm

1-Acryloyl-1,2,3,4-tetrahydroquinoline-6-carboxylic acid (3)

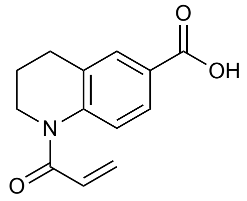

30% (w/w) aq. KOH (1.2 mL) was added to a solution of methyl 1-(2-(phenylselanyl)propanoyl)-1,2,3,4-tetrahydroquinoline-6-carboxylate **S-B** (50 mg, 0.12 mmol) in EtOH (1.2 mL) at rt. After 1 h sat. aq. $NaHCO_3$ (10 mL) was added. The resulting mixture was extracted with EtOAc (3 × 15 mL). The organic layers were combined, dried over Na_2SO_4, filtered, and concentrated under reduced pressure.

EtOH (1.4 mL) was added to the crude material, followed by $NaIO_4$ (60 mg, 0.28 mmol), and the resulting mixture was stirred at 30 ºC for 18 h. The mixture was then filtered through Celite, washing with EtOAc (5 mL). The solvent was removed under reduced pressure and the crude residue was purified by flash column chromatography (30% grading to 50% EtOAc/pentane, with additional 1 mL TFA/500 mL eluent), which afforded acrylamide **3** (26 mg, 80%) as a colourless oil.

R_f 0.44 (50% EtOAc/pentane + 1 drop TFA/25 mL eluent);

ν_{max} (film)/cm^{-1} 2952, 2551, 1685, 1604, 1573, 1405, 1169, 914, 774, 731;

^{1}H NMR (400 MHz, $CDCl_3$) δ 9.60 (br s, 1 H), 7.99–7.89 (m, 2 H), 7.21 (d, *J* = 8.6 Hz, 1 H), 6.59–6.45 (m, 2 H), 5.76 (dd, *J* = 8.5, 3.6 Hz, 1 H), 3.91 (t, *J* = 6.6 Hz, 2 H), 2.82 (t, *J* = 6.4 Hz, 2 H), 2.03 (p, *J* = 6.5 Hz, 2 H);

^{13}C NMR (101 MHz, $CDCl_3$) δ 171.2, 166.3, 143.0, 132.7, 130.5, 129.6, 128.8, 128.3, 125.7, 124.5, 43.9, 27.1, 23.6;

HRMS (ESI$^+$) *m/z* Calculated for $C_{13}H_{14}NO_3^+$ $[M+H]^+$ 232.0974; Found 232.0971 (Δ –1.3 ppm).

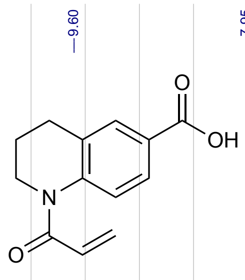

3

^{1}H NMR (400 MHz, $CDCl_3$)

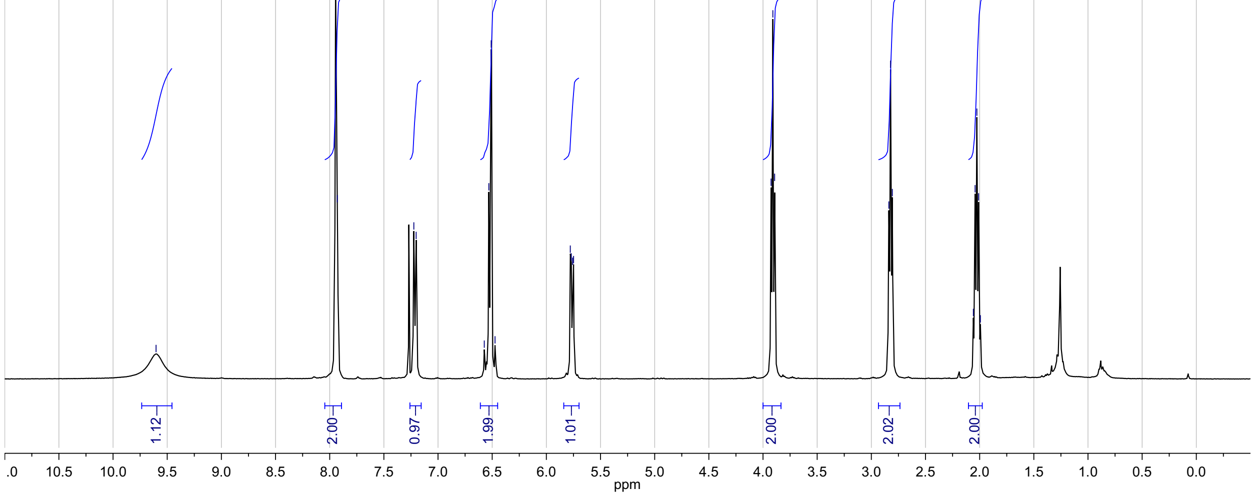

3

^{13}C NMR (101 MHz, $CDCl_3$)

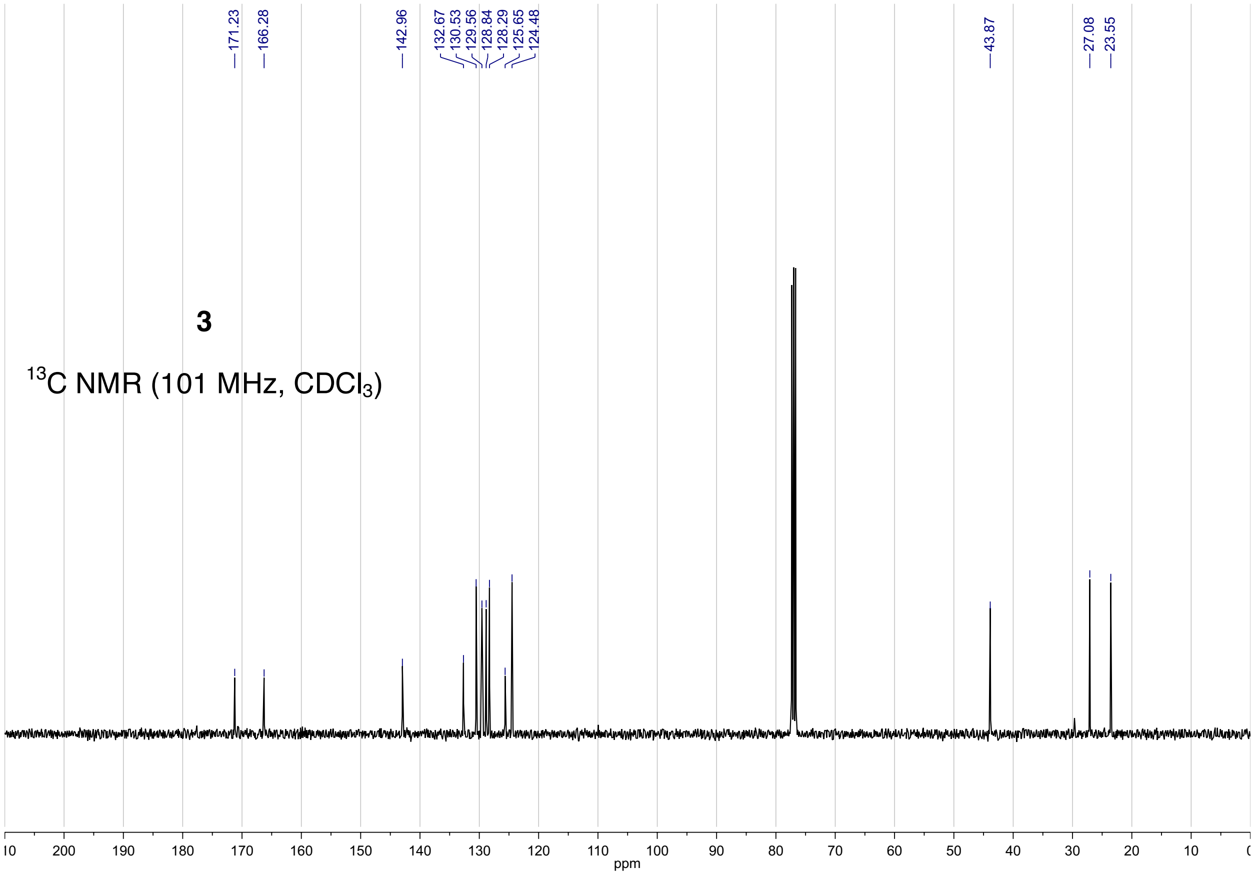

1-Acryloyl-1,2,3,4-tetrahydroquinoline-6-carboxamide (4)

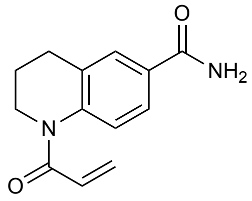

Oxalyl chloride (18 µL, 0.22 mmol) was added dropwise to a solution of 1-(2-(phenylselanyl)propanoyl)-1,2,3,4-tetrahydroquinoline-6-carboxylic acid **S-C** (21 mg, 54 µmol) and DMF (1 drop) in CH_2Cl_2 (3.0 mL) at 0 ˚C. The solution was allowed to warm to rt for 14 h. CH_2Cl_2, DMF and excess oxalyl chloride were removed under reduced pressure. The resulting residue was re-dissolved in CH_2Cl_2 (3.0 mL), then a 7.0 M solution of ammonia in methanol (220 µL, 1.54 mmol) was added to the solution and stirred for 1 h. The reaction was concentrated under reduced pressure and partially-purified by flash column chromatography (EtOAc). The desired primary amide 1-(2-(phenylselanyl)propanoyl)-1,2,3,4-tetrahydroquinoline-6-carboxamide was obtained in >90% purity as a mixture with an inseparable and unknown impurity, and was used in the next step without further purification.

$NaIO_4$ (33 mg, 0.16 mmol) was added to a solution of the crude residue in EtOH (1.0 mL), and was stirred at 30 ºC for 24 h. The mixture was then filtered through Celite, washing with EtOAc (5 mL). The solvent was removed under reduced pressure and the crude residue was purified by flash column chromatography (20% grading to 40% acetone/pentane), which afforded acrylamide **4** (11 mg, 88%) as a white solid.

m.p. = 129–131 ºC (CH_2Cl_2)

R_f 0.20 (30% acetone/pentane)

ν_{max} (film)/cm^{-1} 3355, 3202, 2963, 1644, 1604, 1571, 1501, 1379, 1247, 730;

^{1}H NMR (400 MHz, $CDCl_3$) δ 7.70–7.67 (m, 1 H), 7.61 (dd, *J* = 8.3, 2.1 Hz, 1 H), 7.19 (d, *J* = 8.3 Hz, 1 H), 6.57–6.42 (m, 2 H), 6.28–5.78 (m, 2 H), 5.72 (dd, *J* = 9.2, 2.9 Hz, 1 H), 3.88 (t, *J* = 6.6 Hz, 2 H), 2.80 (t, *J* = 6.5 Hz, 2 H), 2.00 (p, *J* = 6.5 Hz, 2 H).

^{13}C NMR (101 MHz, $CDCl_3$) δ 168.6, 165.8, 141.6, 132.8, 129.7, 129.6, 128.6, 128.0, 125.1, 124.5, 43.7, 27.2, 23.6.

HRMS (ESI$^+$) *m/z* Calculated for $C_{13}H_{15}N_2O_2^+$ $[M+H]^+$ 231.1134, Found 231.1142 (Δ +3.5 ppm).

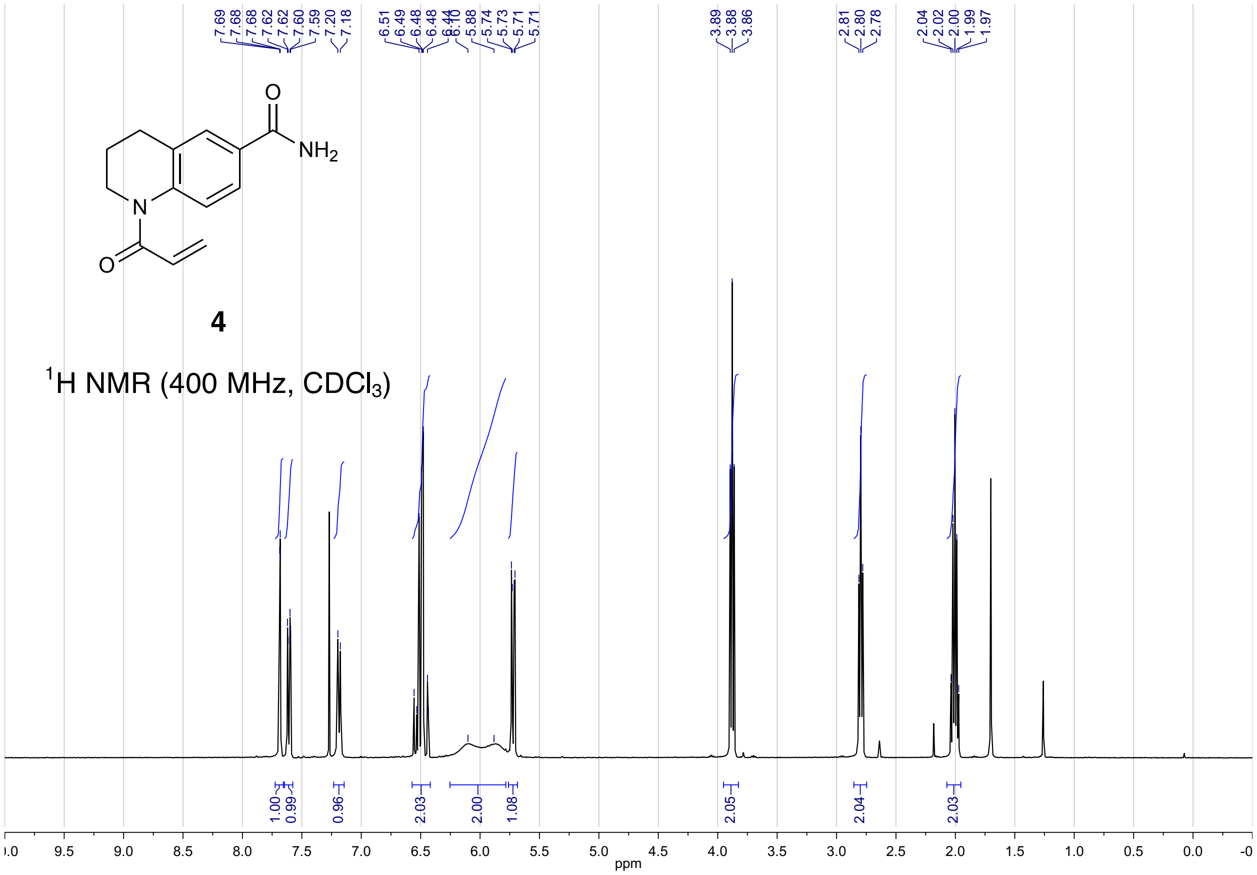

4
^{1}H NMR (400 MHz, CDCl$_3$)
7.69 7.68 7.68 7.62 7.62 7.60 7.59 7.20 7.18 6.51 6.49 6.48 6.48 6.44 6.10 5.88 5.74 5.73 5.71 5.71 3.89 3.88 3.86 2.81 2.80 2.78 2.04 2.02 2.00 1.99 1.97
1.00 0.99 0.96 2.03 2.00 1.08 2.05 2.04 2.03
ppm

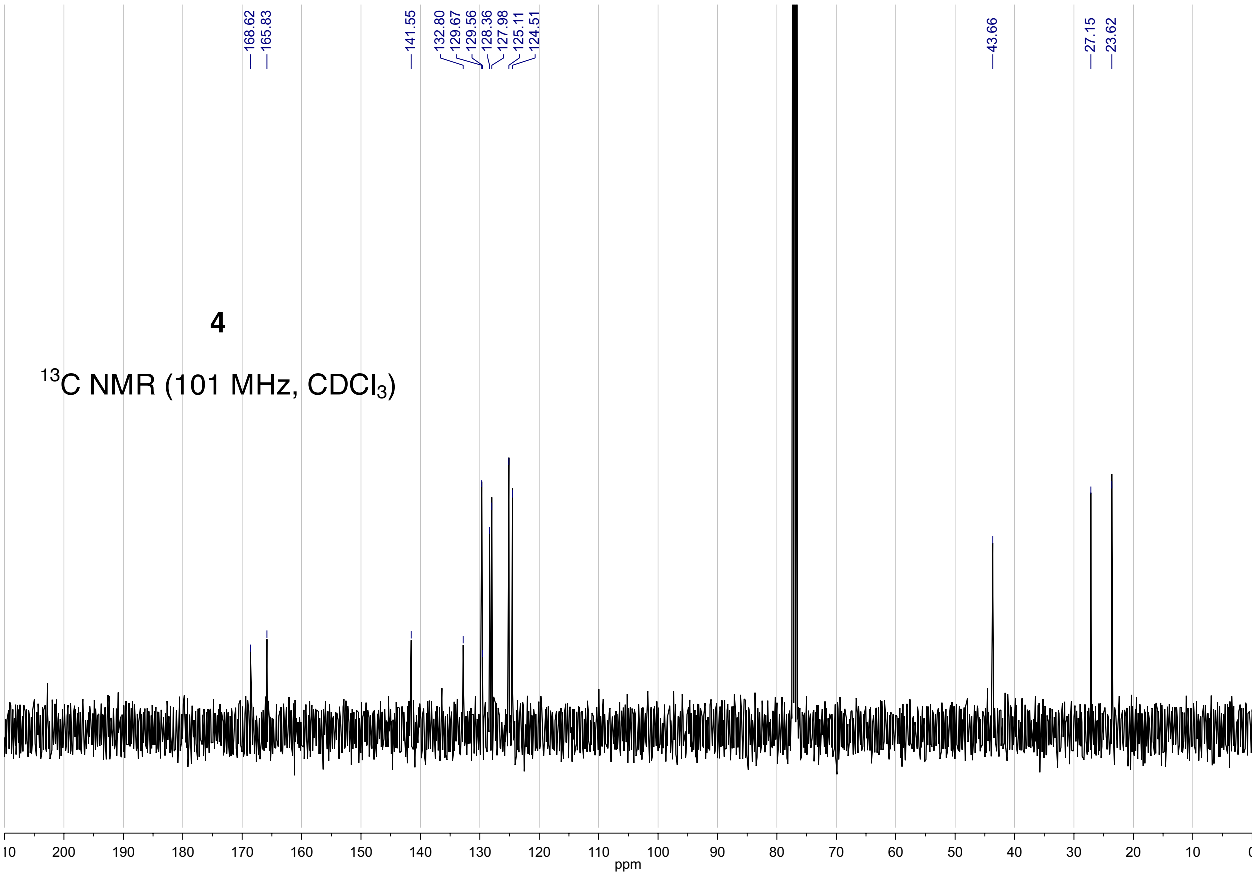

4
^{13}C NMR (101 MHz, CDCl$_3$)
168.62 165.83 141.55 132.80 129.67 129.56 128.36 127.98 125.11 124.51 43.66 27.15 23.62
ppm

1-(2-(Phenylselanyl)propanoyl)-*N*-(prop-2-yn-1-yl)-1,2,3,4-tetrahydroquinoline-6-carboxamide (S-D)

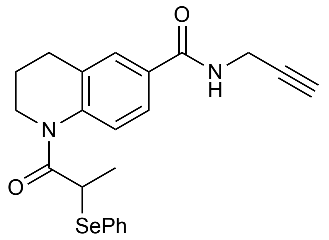

Oxalyl chloride (26 µL, 0.31 mmol) was added dropwise to a solution of 1-(2-(phenylselanyl)propanoyl)-1,2,3,4-tetrahydroquinoline-6-carboxylic acid **S-C** (27 mg, 69 µmol) and DMF (1 drop) in CH_2Cl_2 (3.0 mL) at 0 ˚C. The solution was allowed to warm to rt for 18 h. CH_2Cl_2, DMF and excess oxalyl chloride were removed under reduced pressure. The resulting residue was re-dissolved in CH_2Cl_2 (3.0 mL), then propargylamine (8 µL, 0.15 mmol) and NEt_3 (27 µL, 0.19 mmol) were added. The solution was stirred for 2 h, and was then concentrated under reduced pressure. The crude residue was purified by flash column chromatography (30% grading to 50% EtOAc/pentane) to give 1-(2-(phenylselanyl)propanoyl)-*N*-(prop-2-yn-1-yl)-1,2,3,4-tetrahydroquinoline-6-carboxamide **S-D** (21 mg, 72%) as a white crystalline solid.

m.p. = 69–73 ºC (CH_2Cl_2)

R_f 0.30 (50% EtOAc/pentane)

ν_{max} (film)/cm^{-1} 3296, 2928, 1645, 1608, 1534, 1492, 1379, 1290, 742;

^{1}H NMR (400 MHz, $CDCl_3$) δ 7.61–7.56 (m, 1 H), 7.55–7.49 (m, 1 H), 7.43 (m, 2 H), 7.31–7.24 (m, 2 H), 7.23–7.14 (m, 2 H), 6.41–6.32 (m, 1 H), 4.46–4.34 (m, 1 H), 4.30–4.22 (m, 2 H), 4.06–3.92 (m, 1 H), 3.58–3.45 (m, 1 H), 2.77–2.64 (m, 1 H), 2.64–2.50 (m, 1 H), 2.34–2.28 (m, 1 H), 2.02–1.92 (m, 1 H), 1.90–1.78 (m, 1 H),1.68 (d, *J* = 6.7 Hz, 3 H).

^{13}C NMR (101 MHz, $CDCl_3$) δ 172.9, 166.4, 142.1, 135.2 (2 × C_{Ar}), 130.3, 129.0 (2 × C_{Ar}), 128.3, 127.9, 127.8, 124.7, 124.2, 79.5, 71.9, 44.0, 37.1, 29.8, 26.7, 23.6, 19.2 (1 signal missing due to overlap).

HRMS (ESI$^+$) *m/z* Calculated for $C_{22}H_{23}N_2O_2{}^{80}Se^+$ $[M+H]^+$ 427.0925, Found 427.0935 (Δ +2.3 ppm).

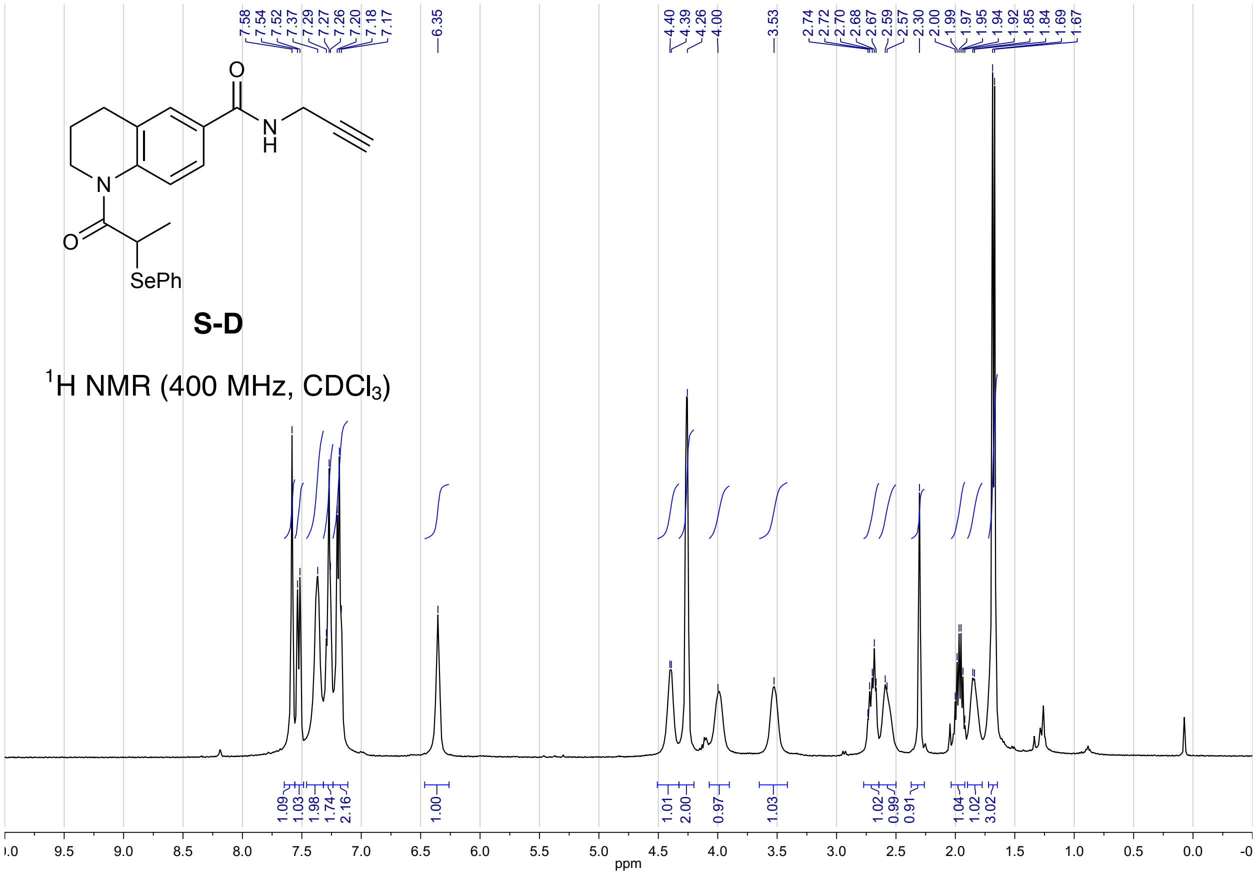
7.58 7.54 7.52 7.37 7.29 7.27 7.26 7.20 7.18 7.17
6.35
4.40 4.39 4.26 4.00
3.53
2.74 2.72 2.70 2.68 2.67 2.59 2.57 2.30 2.00 1.99 1.97 1.95 1.94 1.92 1.85 1.84 1.69 1.67
O
N
H
N
O
SePh
S-D
^{1}H NMR (400 MHz, CDCl$_3$)
1.09 1.03 1.98 1.74 2.16
1.00
1.01 2.00 0.97
1.03
1.02 0.99 0.91
1.04 1.02 3.02
ppm

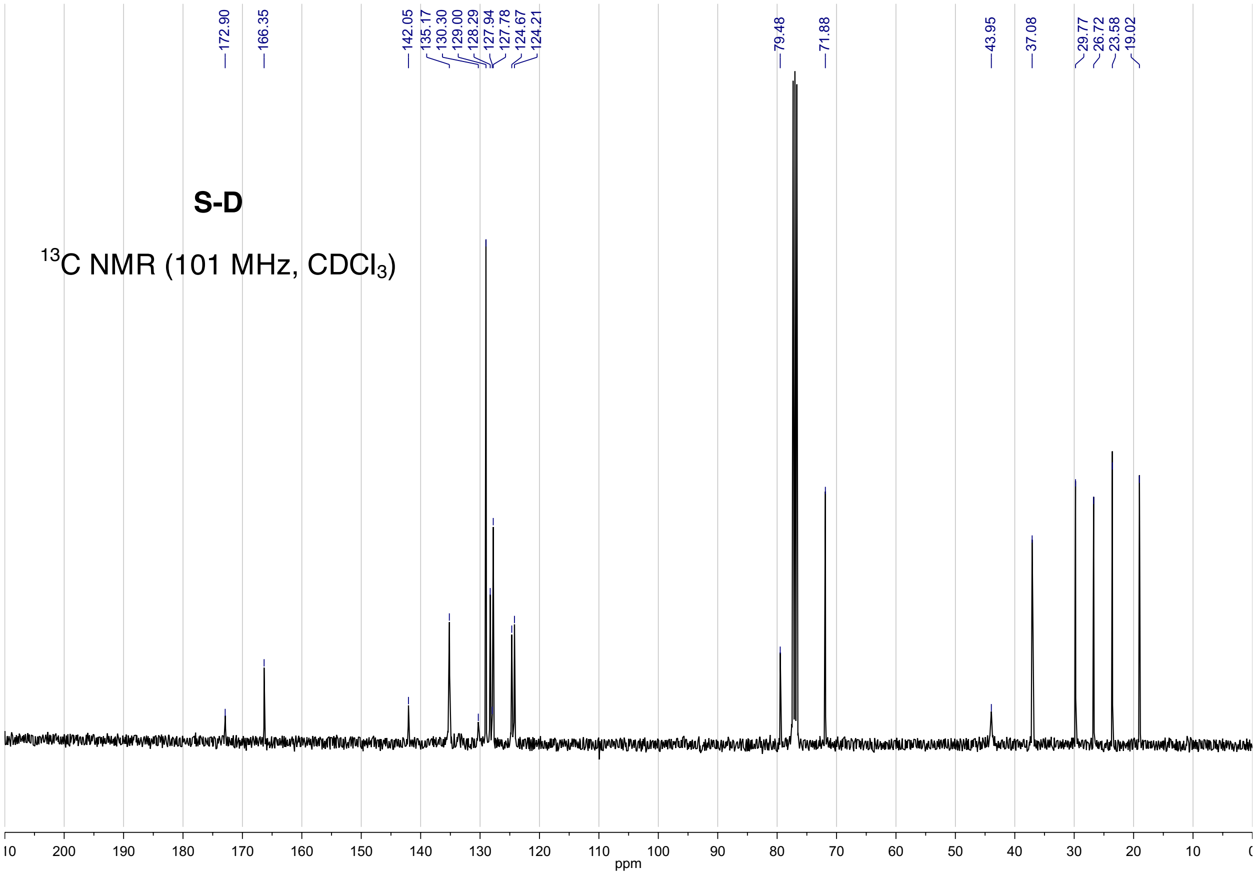
172.90
166.35
142.05 135.17 130.30 129.00 128.29 127.94 127.78 124.67 124.21
79.48
71.88
43.95
37.08
29.77 26.72 23.58 19.02
S-D
^{13}C NMR (101 MHz, CDCl$_3$)
ppm

1-Acryloyl-*N*-(prop-2-yn-1-yl)-1,2,3,4-tetrahydroquinoline-6-carboxamide (5)

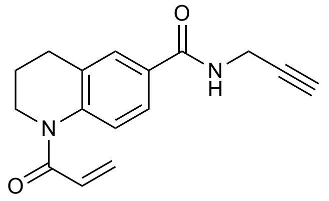

$NaIO_4$ (19 mg, 90 μmol) was added to a solution of 1-(2-(phenylselanyl)propanoyl)-*N*-(prop-2-yn-1-yl)-1,2,3,4-tetrahydroquinoline-6-carboxamide **S-D** (19 mg, 45 μmol) in EtOH (1.0 mL), and was stirred at 30 ºC for 24 h. The mixture was then filtered through Celite, washing with EtOAc (5 mL). The solvent was removed under reduced pressure and the crude residue was purified by flash column chromatography (50% EtOAc/pentane grading to EtOAc), which afforded acrylamide **5** (11 mg, 90%) as a white solid.

m.p. = 137–139 ºC (CH_2Cl_2)

R_f 0.20 (40% EtOAc/pentane)

ν_{max} (film)/cm^{-1} 3300, 2949, 1645, 1608, 1493, 1408, 1292, 1249, 962, 667;

^{1}H NMR (400 MHz, $CDCl_3$) δ 7.67–7.63 (m, 1 H), 7.59 (dd, *J* = 8.3, 2.2 Hz, 1 H), 7.18 (d, *J* = 8.3 Hz, 1 H), 6.56–6.42 (m, 2 H), 6.36–6.28 (m, 1 H), 5.71 (dd, *J* = 9.0, 3.2 Hz, 1 H), 4.26 (dd, *J* = 5.2, 2.6 Hz, 2 H), 3.87 (t, *J* = 6.6 Hz, 2 H), 2.79 (t, *J* = 6.5 Hz, 2 H), 2.29 (t, *J* = 2.6 Hz, 1 H), 2.00 (p, *J* = 6.6 Hz, 2 H).

^{13}C NMR (101 MHz, $CDCl_3$) δ 166.4, 165.8, 141.4, 132.8, 130.0, 129.7, 128.3, 127.6, 124.7, 124.6, 79.4, 71.9, 43.6, 29.8, 27.2, 23.6.

HRMS (ESI$^+$) *m/z* Calculated for $C_{16}H_{17}N_2O_2^+$ $[M+H]^+$ 269.1290, Found 269.1283 (Δ –2.6 ppm).

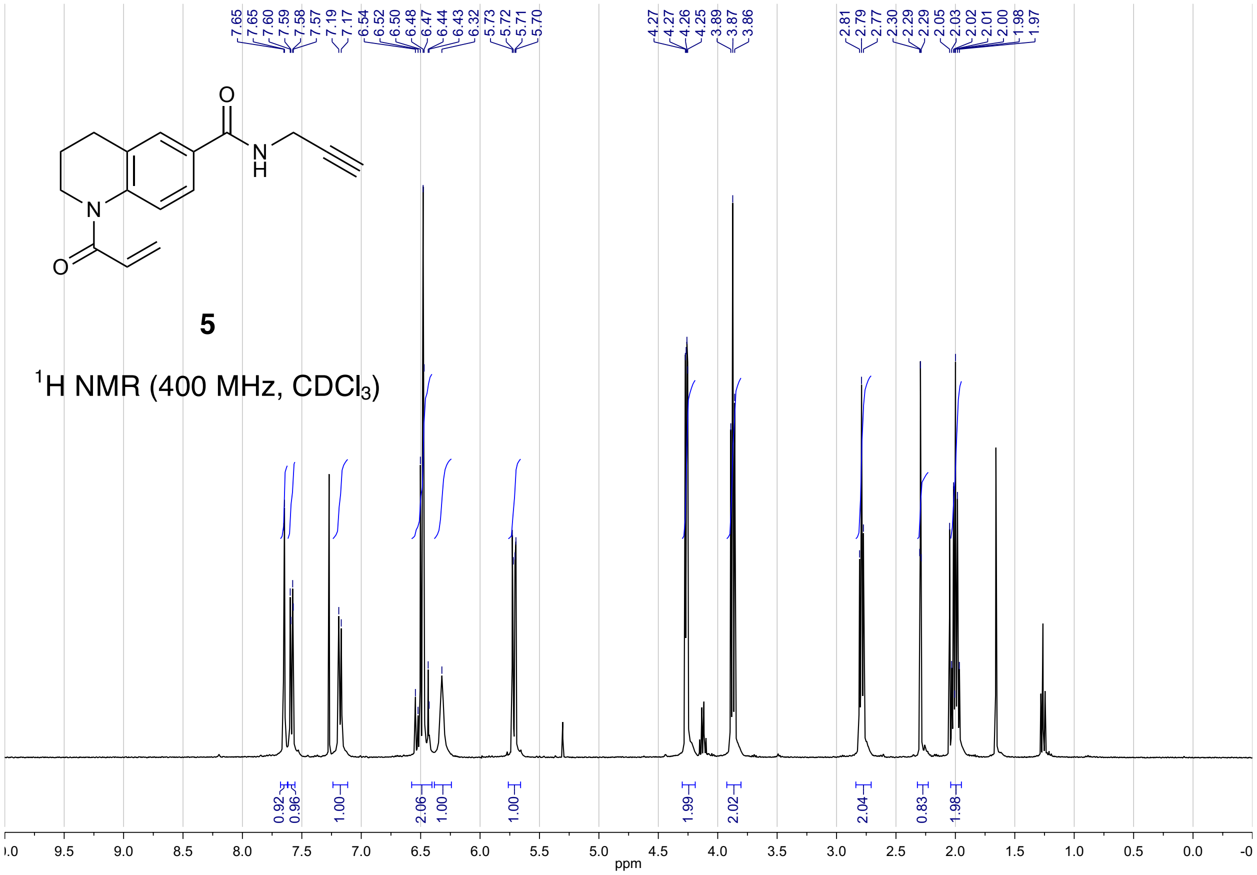
7.65 7.65 7.60 7.59 7.58 7.57 7.19 7.17 6.54 6.52 6.50 6.48 6.47 6.44 6.43 6.32 5.73 5.72 5.71 5.70 4.27 4.27 4.26 4.25 3.89 3.87 3.86 2.81 2.79 2.77 2.30 2.29 2.29 2.05 2.03 2.02 2.01 2.00 1.98 1.97
O
N
H
N
O
5
¹H NMR (400 MHz, CDCl3)
0.92 0.96 1.00 2.06 1.00 1.00 1.99 2.02 2.04 0.83 1.98
ppm

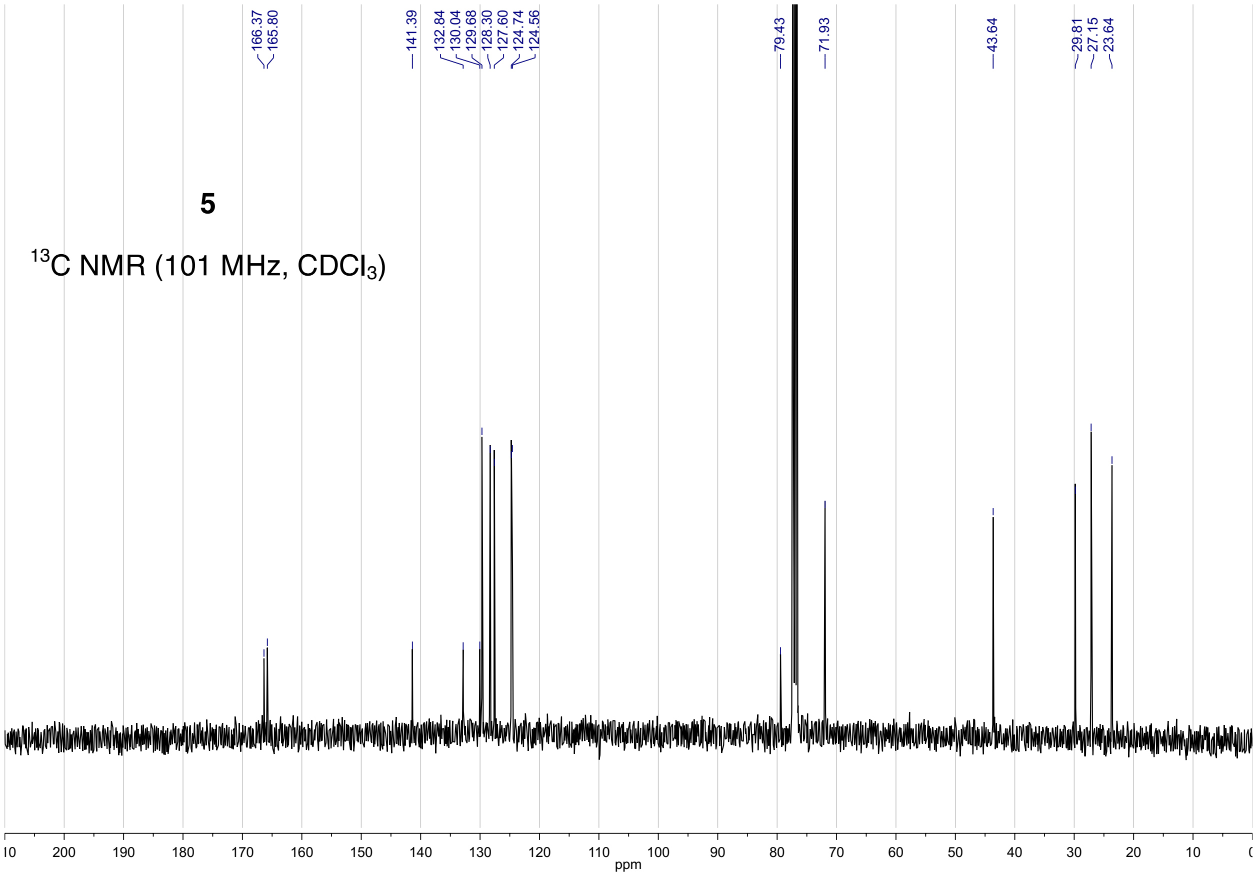
166.37 165.80 141.39 132.84 130.04 129.68 128.30 127.60 124.74 124.56 79.43 71.93 43.64 29.81 27.15 23.64
5
¹³C NMR (101 MHz, CDCl3)
ppm

1-(4-Ethyl-3,4-dihydroquinoxalin-1(2*H*)-yl)-2-(phenylselanyl)propan-1-one (S-E)

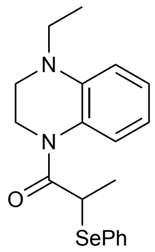

Modifying Zhang's procedure,[3] bromoethane (37 mL, 0.50 mmol) was added to a solution of 1-(3,4-dihydroquinoxalin-1(2*H*)-yl)-2-(phenylselanyl)propan-1-one **S-E** (86 mg, 0.25 mmol) and KOH (28 mg, 0.50 mmol) in DMSO (1.0 mL). The reaction mixture was heated to 50 ºC for 24 h. H_2O (20 mL) was then added and the mixture was extracted with EtOAc (3 × 30 mL). The organic layers were combined, dried over Na_2SO_4, filtered, and the solvent was removed under reduced pressure. The crude residue was purified by flash column chromatography (20% EtOAc/pentane), which afforded 1-(4-ethyl-3,4-dihydroquinoxalin-1(2*H*)-yl)-2-(phenylselanyl)propan-1-one **S-E** (33 mg, 36%) as a colourless oil.

R_f 0.68 (40% EtOAc/pentane);

ν_{max} (film)/cm^{-1} 2969, 2929, 1646, 1602, 1507, 1377, 1340, 1275, 1188, 738;

^{1}H NMR (400 MHz, $CDCl_3$) δ 7.34–7.28 (m, 2 H), 7.26–7.21 (m, 1 H), 7.19–7.12 (m, 2 H), 7.12–7.05 (m, 1 H), 6.98 (d, *J* = 7.8 Hz, 1 H), 6.69 (dd, *J* = 8.3, 1.2 Hz, 1 H), 6.56 (t, *J* = 7.4 Hz, 1 H), 4.61–4.53 (m, 1 H), 4.35–4.23 (m, 1 H), 3.53–3.41 (m, 1 H), 3.34 (qd, *J* = 7.2, 2.4 Hz, 2 H), 3.30–3.24 (m, 2 H), 1.69–1.63 (m, 3 H), 1.15 (t, *J* = 7.1 Hz, 3 H);

^{13}C NMR (101 MHz, $CDCl_3$) δ 172.4, 139.2, 134.9 (2 × C_{Ar}), 128.9 (2 × C_{Ar}), 127.8, 127.1, 124.7, 124.4, 114.9, 111.2, 47.4, 44.5, 39.8, 36.8, 19.0, 11.1 (1 signal missing due to overlap);

HRMS (ESI$^+$) *m/z* Calculated for $C_{19}H_{23}N_2O^{80}Se^+$ $[M+H]^+$ 375.0976; Found 375.0976 (Δ 0.0 ppm).

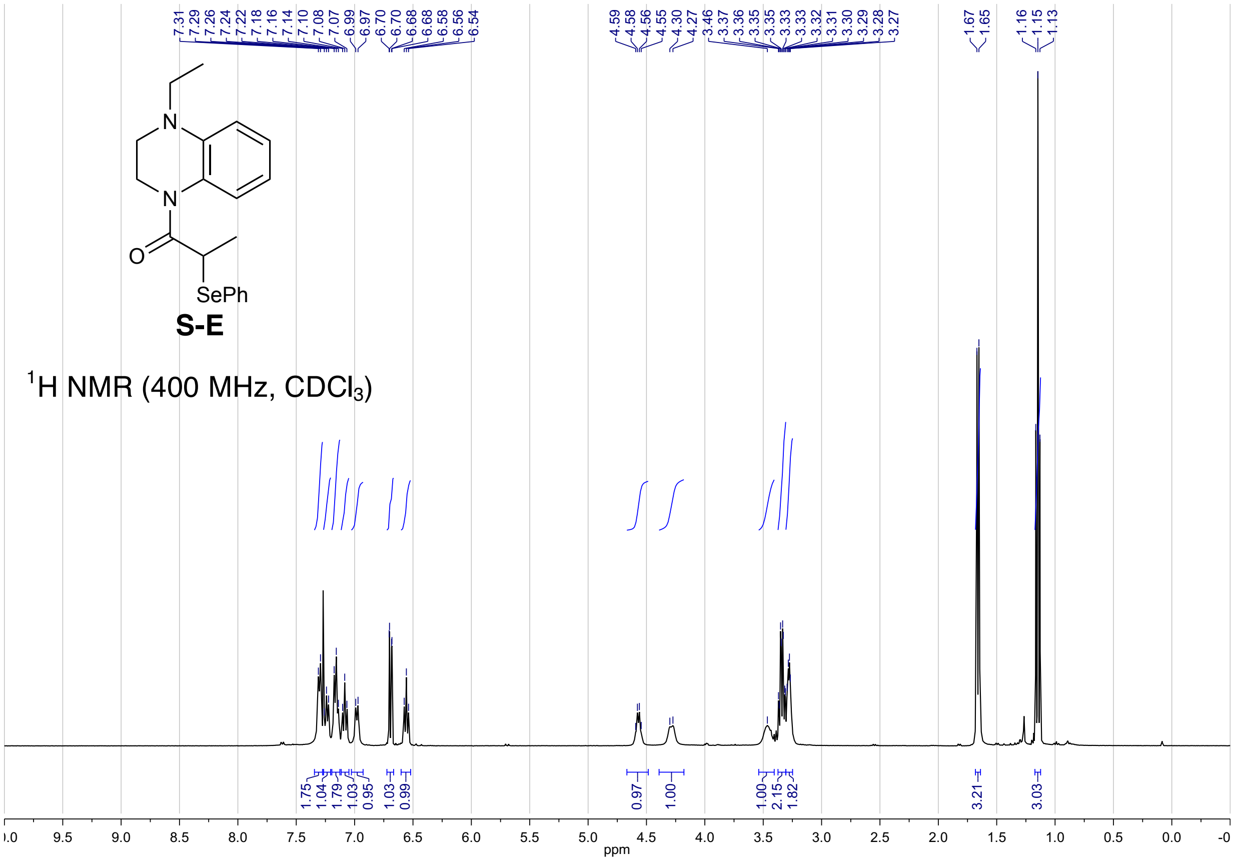

S-E

^{1}H NMR (400 MHz, $CDCl_3$)

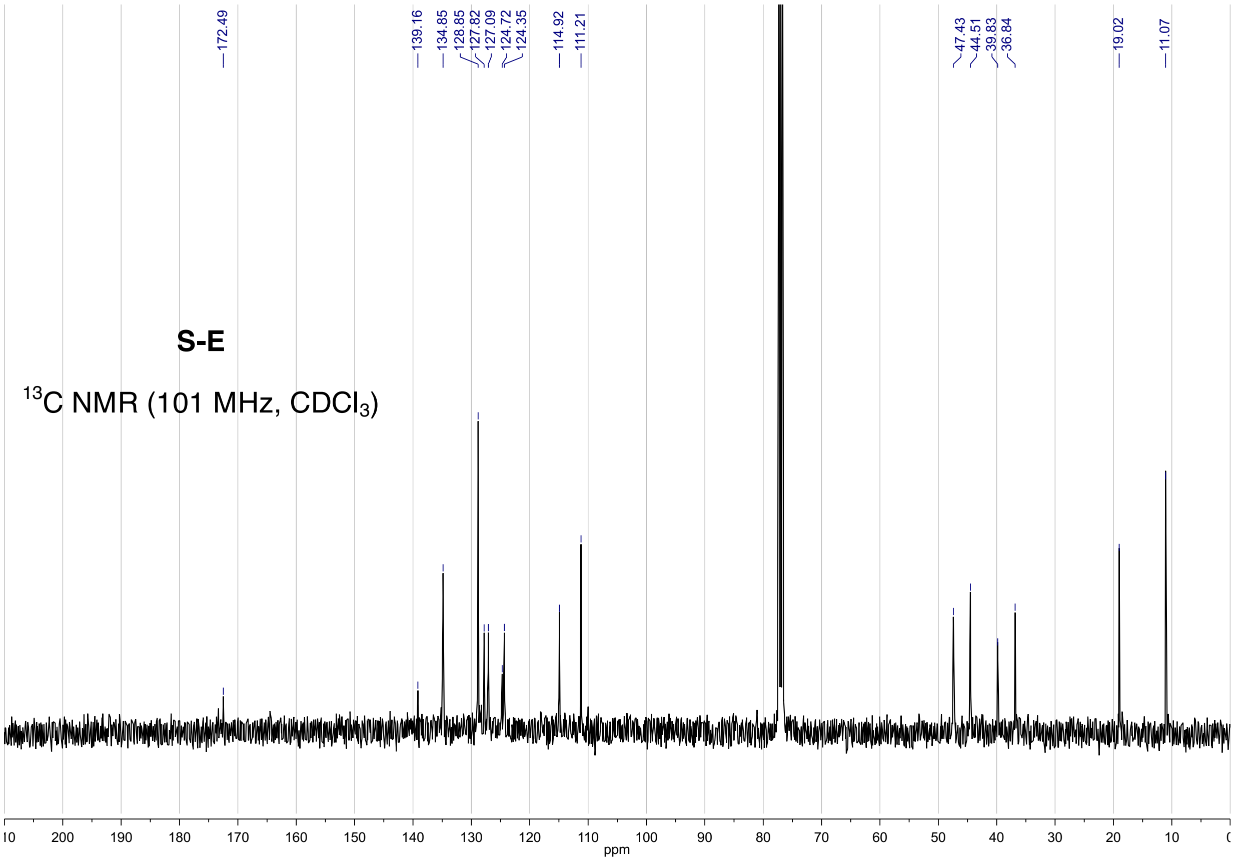

S-E

^{13}C NMR (101 MHz, $CDCl_3$)

1-(4-Ethyl-3,4-dihydroquinoxalin-1(2*H*)-yl)prop-2-en-1-one (6)

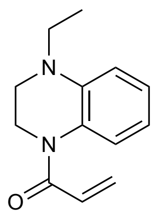

$NaIO_4$ (39 mg, 0.18 mmol) was added to a solution of 1-(4-ethyl-3,4-dihydroquinoxalin-1(2*H*)-yl)-2-(phenylselanyl)propan-1-one **S-E** (34 mg, 0.09 mmol) in EtOH (0.91 mL), and was stirred at 30 ºC for 18 h. The mixture was then filtered through Celite, washing with EtOAc (5 mL). The solvent was removed under reduced pressure and the crude residue was purified by flash column chromatography (10% EtOAc/pentane), which afforded acrylamide **6** (14 mg, 72%) as a yellow oil.

R_f 0.56 (25% EtOAc/pentane);

ν_{max} (film)/cm^{-1} 2971, 1652, 1603, 1508, 1413, 1368, 1186, 742;

^{1}H NMR (400 MHz, $CDCl_3$) δ 7.10–7.05 (m, 1 H), 6.97–6.90 (m, 1 H), 6.74–6.63 (m, 2 H), 6.63–6.57 (m, 1 H), 6.45 (dd, *J* = 16.8, 2.1 Hz, 1 H), 5.69 (dd, *J* = 10.3, 2.0 Hz, 1 H), 3.99 (t, *J* = 5.5 Hz, 2 H), 3.45–3.36 (m, 4 H), 1.18 (t, *J* = 7.1 Hz, 3 H).

^{13}C NMR (101 MHz, $CDCl_3$) δ 164.1, 138.6, 129.4, 127.7, 126.7, 124.9, 124.3, 115.0, 111.0, 48.1, 44.6, 39.4, 11.0;

HRMS (ESI$^+$) *m/z* Calculated for $C_{13}H_{17}N_2O^+$ $[M+H]^+$ 217.1341; Found 217.1345 (Δ +1.8 ppm).

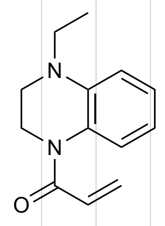

6

^{1}H NMR (400 MHz, $CDCl_3$)

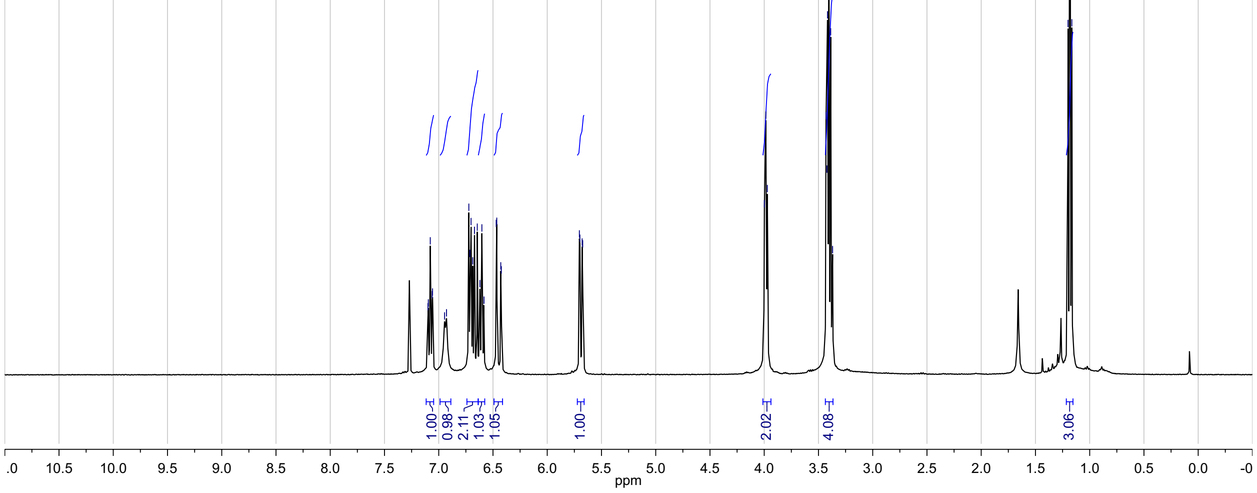

6

^{13}C NMR (101 MHz, $CDCl_3$)

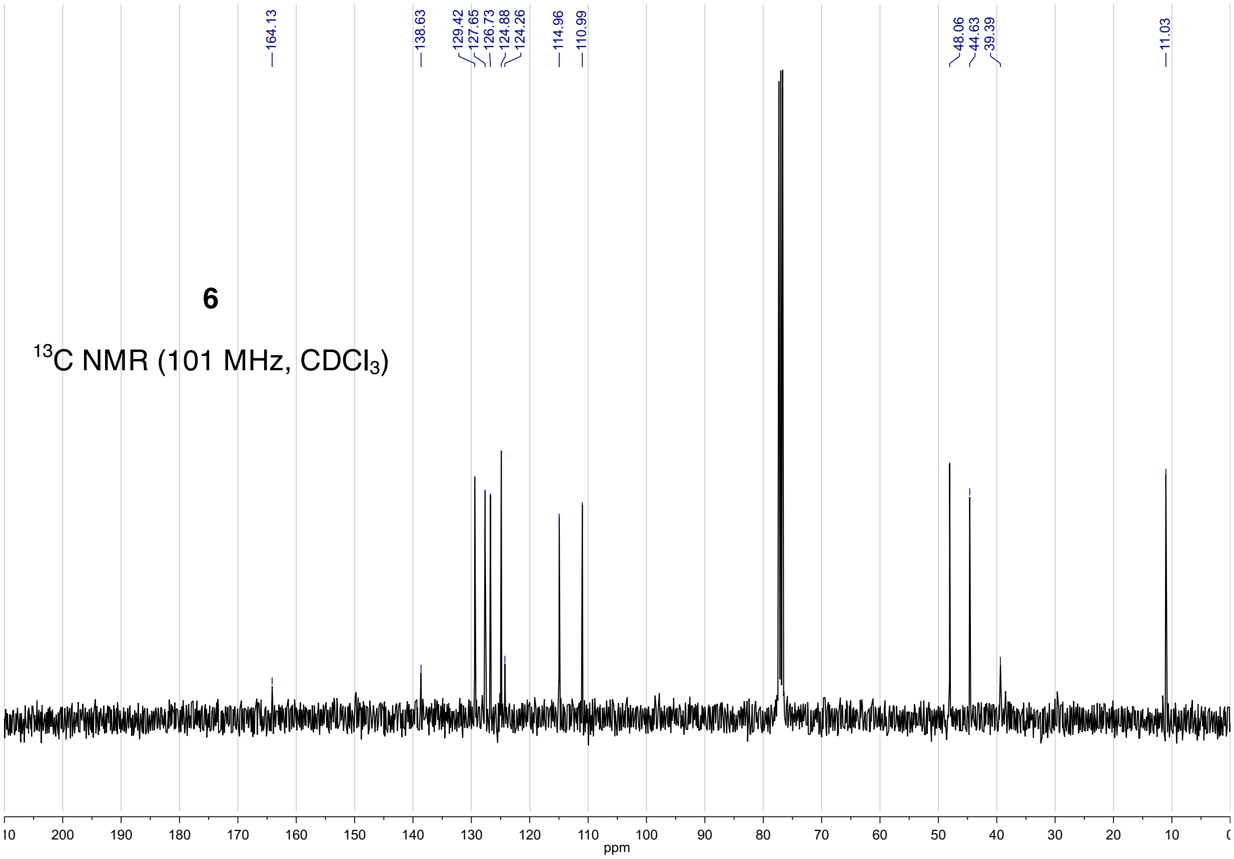

1-(4-Acetyl-3,4-dihydroquinoxalin-1(2*H*)-yl)-2-(phenylselanyl)propan-1-one (S-F)

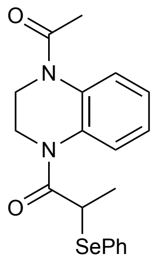

Acetic anhydride (50 μL, 0.52 mmol) was added dropwise to a solution of 1-(3,4-dihydroquinoxalin-1(2*H*)-yl)-2-(phenylselanyl)propan-1-one **S-A** (86 mg, 0.25 mmol) in CH_2Cl_2 (1.0 mL) at 0 ºC. The resulting mixture was allowed to warm to rt for 6 h. Sat. aq. $NaHCO_3$ (15 mL) and CH_2Cl_2 (15 mL) were then added and the phases were separated. The organic layer was collected, and the aqueous layer was extracted with further CH_2Cl_2 (2 × 15 mL). The combined organic layers were dried over Na_2SO_4 and filtered, then the solvent was removed under reduced pressure. The resulting crude material was then purified by flash column chromatography (30% grading to 60% EtOAc/pentane), which gave 1-(4-acetyl-3,4-dihydroquinoxalin-1(2*H*)-yl)-2-(phenylselanyl)propan-1-one **S-F** (52 mg, 54%) as a colourless oil.

R_f 0.13 (40% EtOAc/pentane);

ν_{max} (film)/cm^{-1} 2925, 1651, 1496, 1371, 1326, 1216, 758, 738, 692;

^{1}H NMR (400 MHz, $CDCl_3$) δ 7.46–7.11 (m, 9 H), 4.42–4.15 (m, 3 H), 3.76–3.50 (m, 2 H), 2.25 (s, 3 H), 1.63 (d, *J* = 6.8 Hz, 3 H);

^{13}C NMR (101 MHz, $CDCl_3$) δ 171.6, 169.6, 135.8 (2 × C_{Ar}), 129.0 (2 × C_{Ar}), 128.61, 126.60, 126.56, 126.5, 125.8, 125.2, 124.8, 36.2, 29.6, 23.0, 18.7 (2 signals missing due to overlap);

This compound appeared as a mixture of rotamers in the NMR spectra.

HRMS (ESI$^+$) *m/z* Calculated for $C_{19}H_{21}N_2O_2{}^{80}Se^+$ $[M+H]^+$ 389.0768; Found 389.0757 (Δ –2.8 ppm).

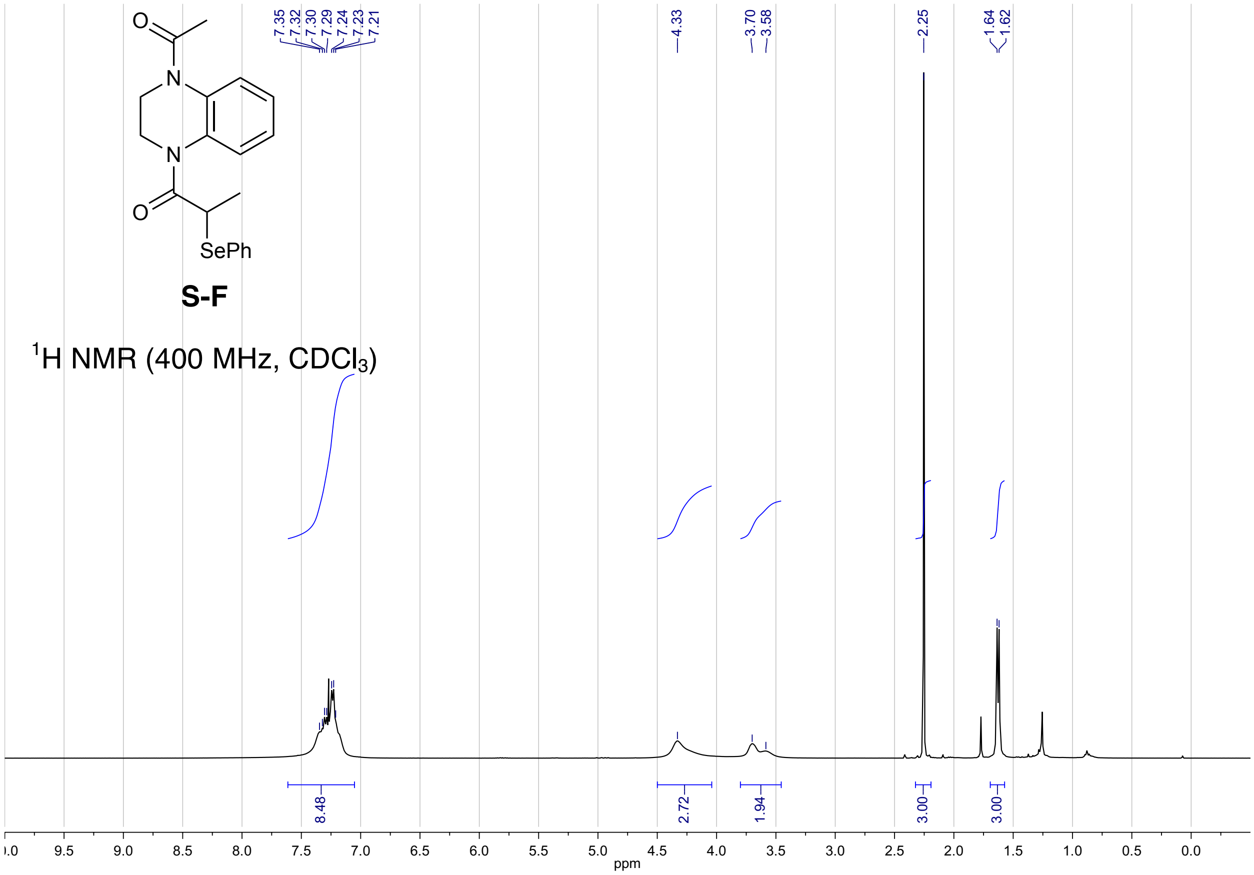

S-F

^{1}H NMR (400 MHz, $CDCl_3$)

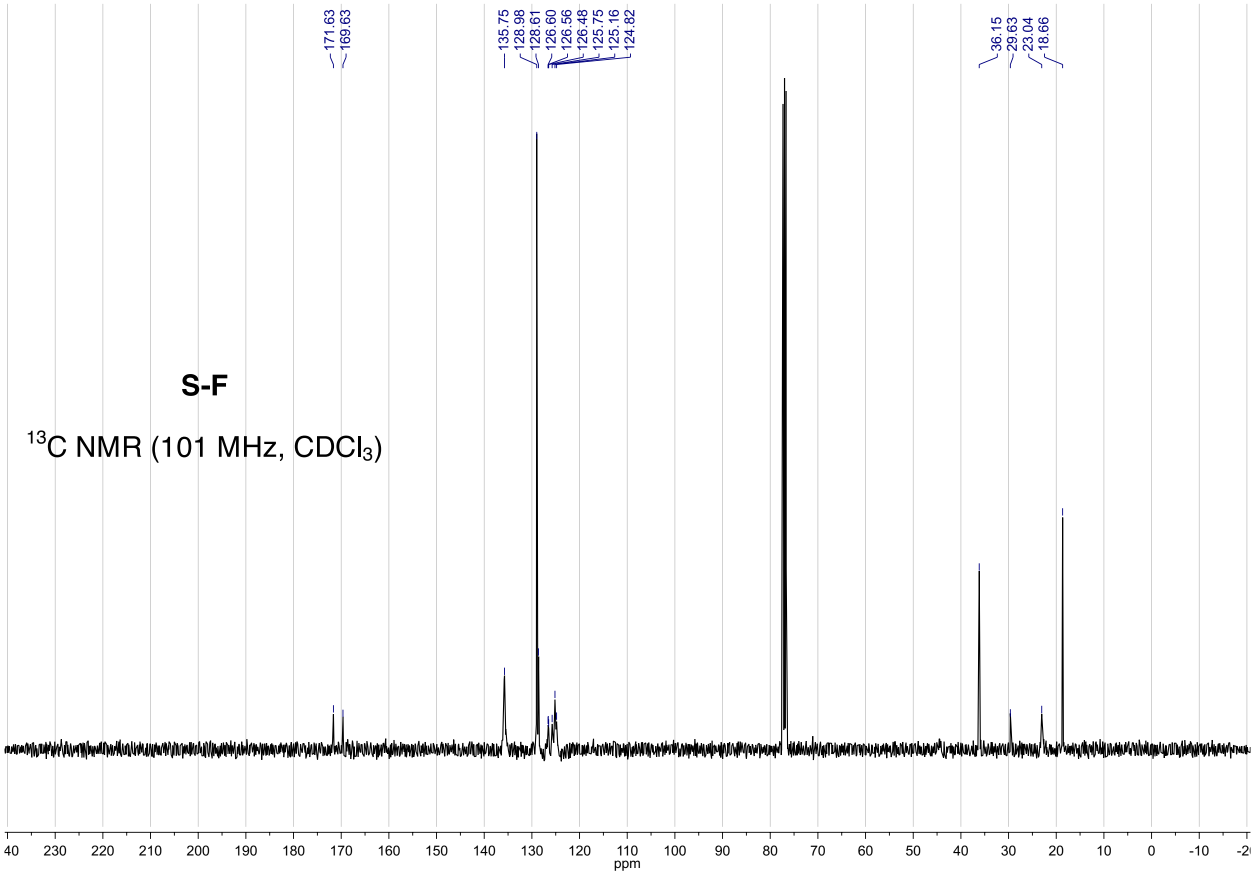

S-F

^{13}C NMR (101 MHz, $CDCl_3$)

1-(4-Acetyl-3,4-dihydroquinoxalin-1(2*H*)-yl)prop-2-en-1-one (7)

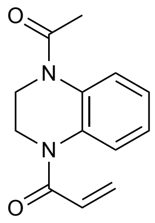

$NaIO_4$ (56 mg, 0.26 mmol) was added to a solution of 1-(4-acetyl-3,4-dihydroquinoxalin-1(2*H*)-yl)-2-(phenylselanyl)propan-1-one **S-F** (52 mg, 0.13 mmol) in EtOH (1.3 mL), and was stirred at 30 ºC for 18 h. The mixture was then filtered through Celite, washing with EtOAc (5 mL). The solvent was removed under reduced pressure and the crude residue was purified by flash column chromatography (50% EtOAc/pentane grading to EtOAc) which afforded acrylamide **7** (26 mg, 87%) as a white solid.

mp = 119–121 ºC (EtOAc/pentane)

R_f 0.34 (EtOAc);

ν_{max} (film)/cm^{-1} 1652, 1497, 1407, 1326, 1217, 760;

^{1}H NMR (400 MHz, $CDCl_3$) δ 7.26–7.14 (m, 4 H), 6.58 (dd, *J* = 16.8, 9.9 Hz, 1 H), 6.48 (dd, *J* = 16.7, 2.2 Hz, 1 H), 5.74 (dd, *J* = 10.0, 2.2 Hz, 1 H), 4.06–3.93 (m, 4 H), 2.26 (s, 3 H);

^{13}C NMR (101 MHz, $CDCl_3$) δ 169.1, 164.7, 134.0, 133.3, 128.9, 128.7, 125.8, 125.0, 124.8, 45.2, 43.9, 22.7 (1 signal missing due to overlap);

HRMS (ESI$^+$) *m/z* Calculated for $C_{13}H_{15}N_2O_2^+$ [M+H]$^+$ 231.1134; Found 231.1140 (Δ +2.6 ppm).

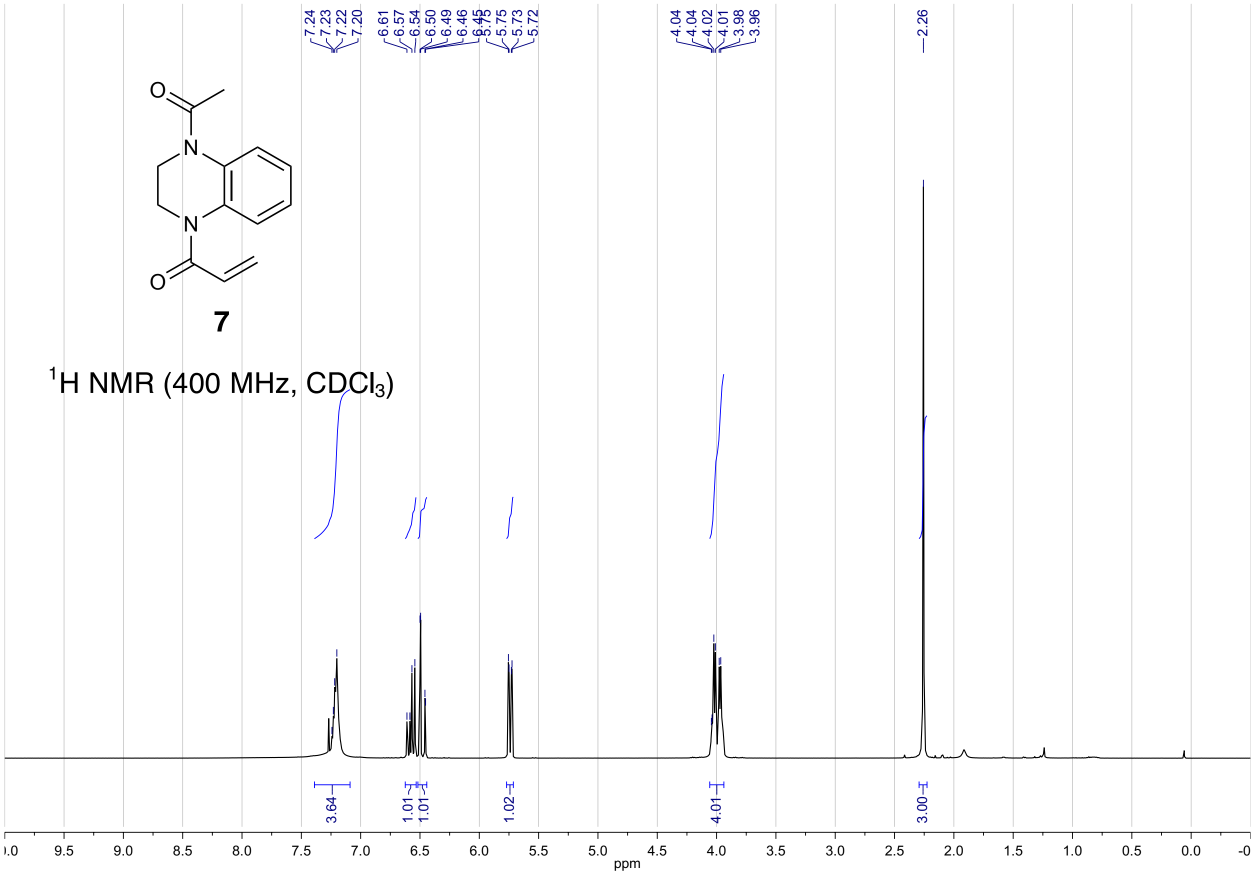

7

^{1}H NMR (400 MHz, $CDCl_3$)

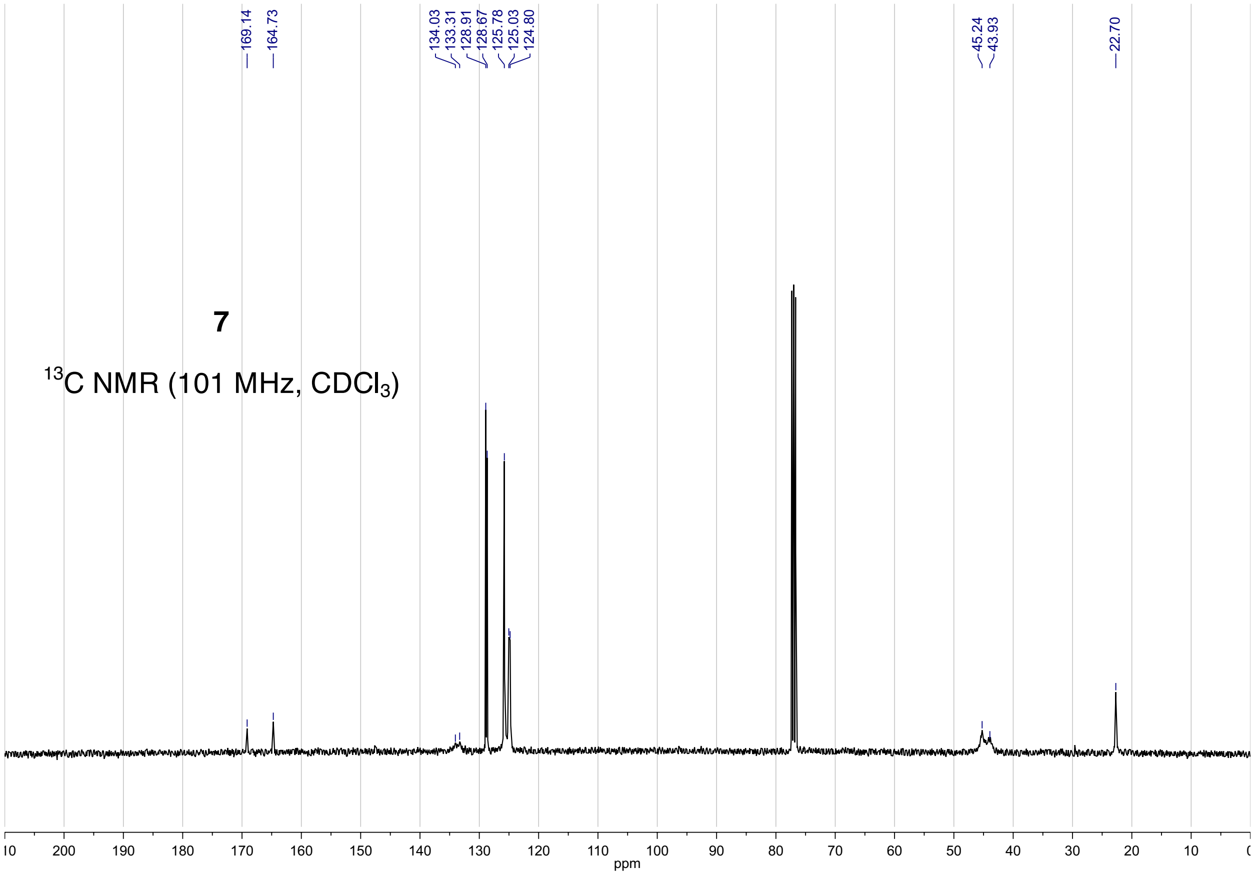

7

^{13}C NMR (101 MHz, $CDCl_3$)

Methyl 1-acryloylindoline-5-carboxylate (8)

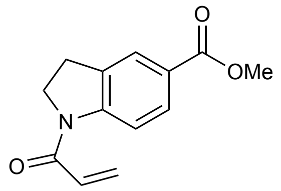

Using our previously reported procedure,[2] a solution of acryloyl chloride in CH_2Cl_2 (160 µL, 3.6 M, 0.56 mmol) was added to a solution of methyl indoline-5-carboxylate (100 mg, 0.56 mmol) in $CHCl_3$ (1.6 mL). After 5 min, Amberlyst A26(OH) resin (148 mg) was added, and the resulting suspension was stirred at rt for a further 5 min. The reaction mixture was then filtered through Celite washing with CH_2Cl_2 (5 mL) and concentrated under reduced pressure. The crude residue was purified by flash column chromatography (10% grading to 40% EtOAc/pentane), which afforded acrylamide **8** (82 mg, 63%) as a white solid.

m.p. = 114–116 ºC (CH_2Cl_2);

R_f 0.20 (20% EtOAc/pentane)

ν_{max} (film)/cm^{-1} 2952, 1709, 1654, 1602, 1487, 1442, 1412, 1269, 1202, 1084, 767;

^{1}H NMR (400 MHz, $CDCl_3$) δ 8.27 (br s, 1 H), 7.91–7.87 (m, 1 H), 7.81 (br s, 1 H), 6.63–6.45 (m, 2 H), 5.84–5.78 (m, 1 H), 4.21–4.14 (m, 2 H), 3.87 (s, 3 H), 3.19 (t, J = 8.5 Hz, 2 H).

^{13}C NMR (101 MHz, $CDCl_3$) δ 166.6, 164.2, 146.7, 131.7, 130.0, 129.7, 128.6, 125.9, 125.4, 116.5, 51.9, 48.4, 27.4.

HRMS (ESI$^+$) *m/z* Calculated for $C_{13}H_{14}NO_3^+$ [M+H]$^+$ 232.0974, Found 232.0978 (Δ +1.7 ppm).

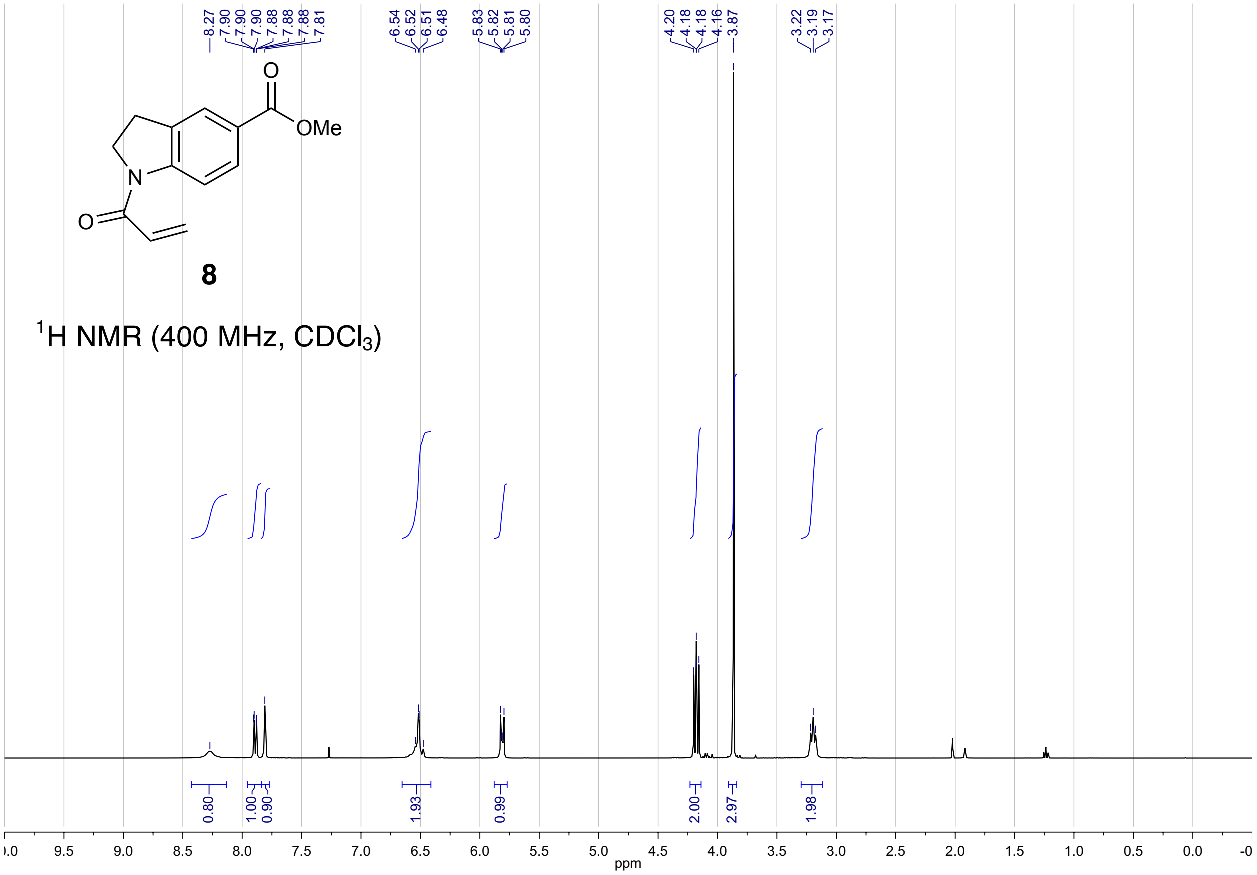

8

^{1}H NMR (400 MHz, $CDCl_3$)

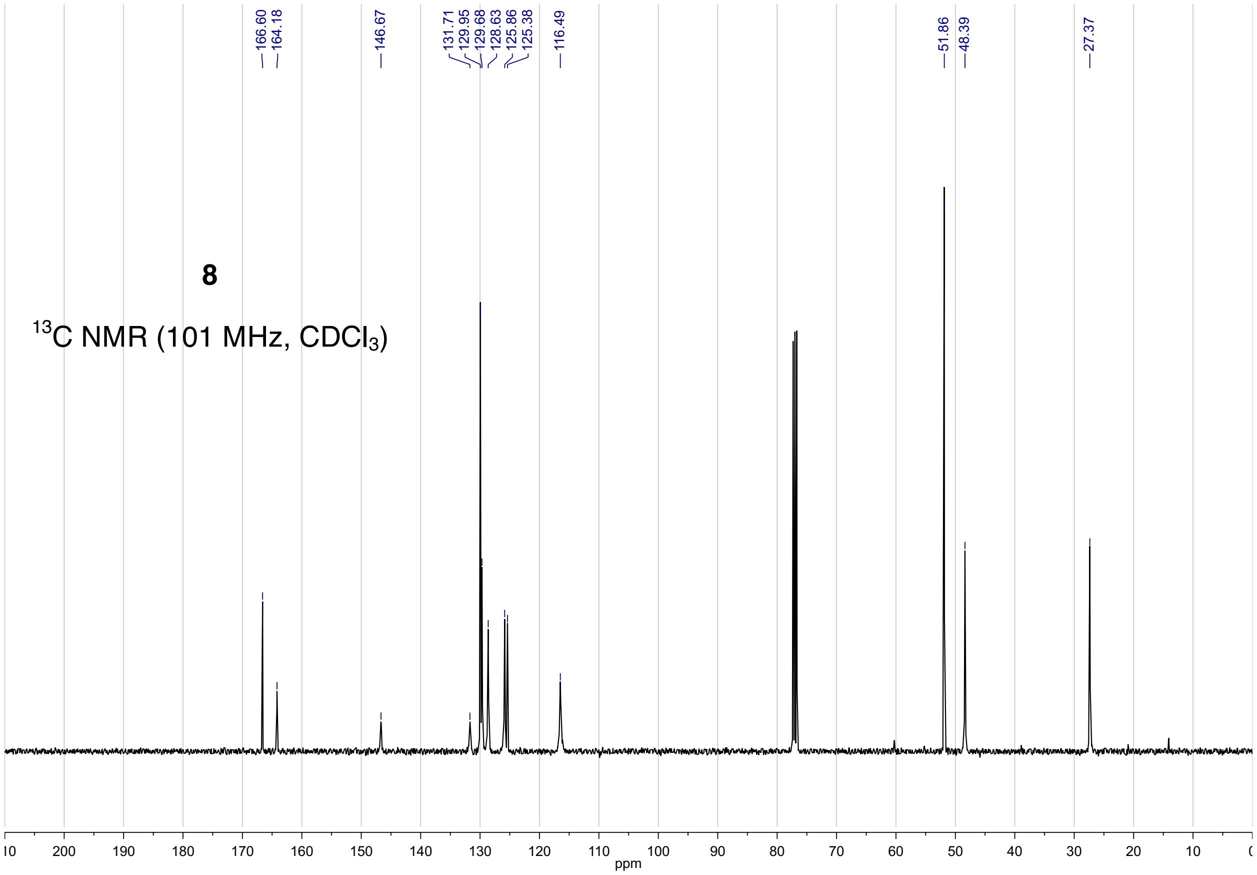

8

^{13}C NMR (101 MHz, $CDCl_3$)

1-(*tert*-Butyl) 6-ethyl 3,4-dihydroquinoxaline-1,6(2*H*)-dicarboxylate (S-G)

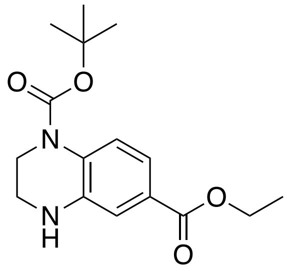

Ethyl 3-amino-4-((*tert*-butoxycarbonyl)amino)benzoate[1] (300 mg, 1.07 mmol) was added to a solution of K_2CO_3 (591 mg, 4.28 mmol) and dibromoethane (461 µL, 5.35 mmol) in DMF (2.10 mL, 0.5 M). The mixture was stirred at 100 °C for 16 h, before filtering the solution through Celite and washing with EtOAc (50 mL). The solvent was removed under reduced pressure. Purification of the crude material by flash column chromatography (15% EtOAc/pentane) afforded amine **S-G** (92 mg, 28%) as a pale yellow solid.

m.p. = 121 ºC (CH_2Cl_2);

R_f 0.18 (15% EtOAc/pentane)

ν_{max} (film)/cm^{-1} 3389, 2979, 1698, 1503, 1367, 1220, 1165, 1149, 1106, 766;

^{1}H NMR (400 MHz, $CDCl_3$) δ 7.58 (d, *J* = 7.4 Hz, 1 H), 7.33 (dd, *J* = 8.6, 1.9 Hz, 1 H), 7.26 (m, 1 H), 4.33 (q, *J* = 7.1 Hz, 2 H, OCH_2CH_3), 3.79 – 3.77 (m, 2 H), 3.43 – 3.40 (m, 2 H), 1.52 (s, 9 H), 1.36 (t, *J* = 7.1 Hz, 3 H)
^{13}C NMR (101 MHz, $CDCl_3$) δ 166.6, 152.9, 136.3, 128.7, 126.2, 123.8, 118.0, 115.6, 81.5, 60.7, 41.7, 41.4, 28.3, 14.3;

HRMS (ESI$^+$) *m/z* Calculated for $C_{16}H_{23}N_2O_4^+$ $[M+H]^+$ 307.1658; Found 307.1652 (Δ = 2.0 ppm).

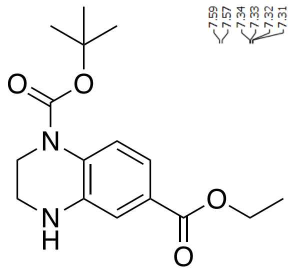

S-G

^{1}H NMR (400 MHz, $CDCl_3$)

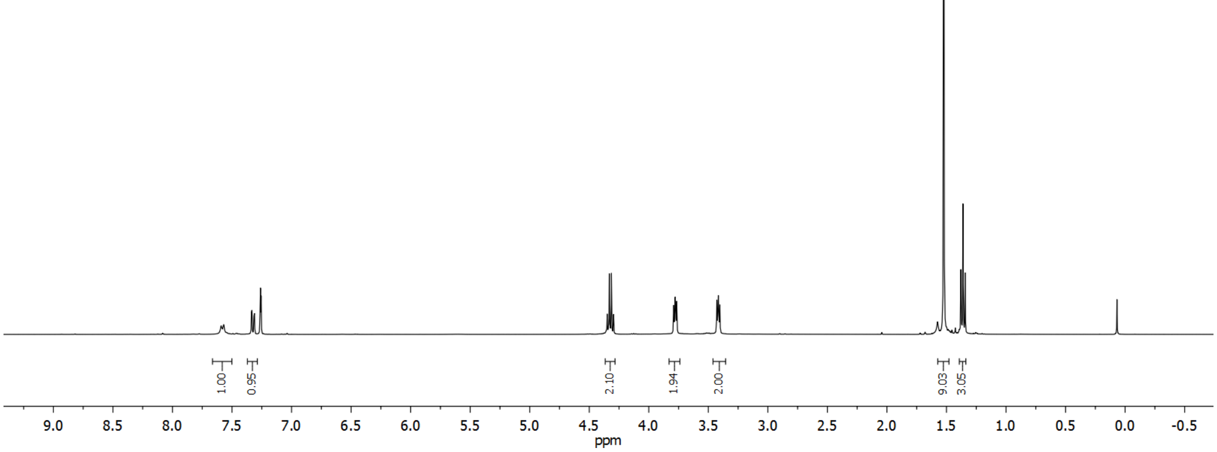

S-G

^{13}C NMR (101 MHz, $CDCl_3$)

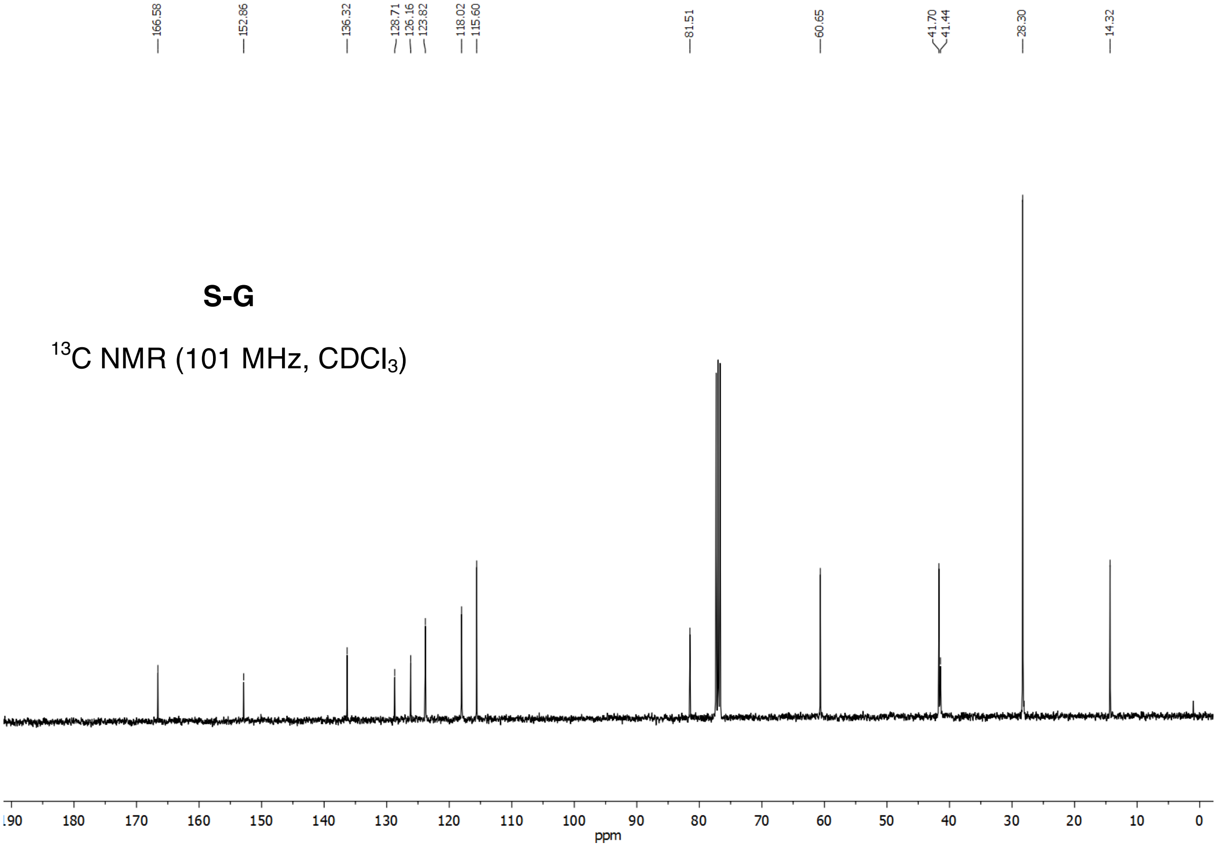

1-(*tert*-Butyl) 6-ethyl 4-ethyl-3,4-dihydroquinoxaline-1,6(2*H*)-dicarboxylate (S-H)

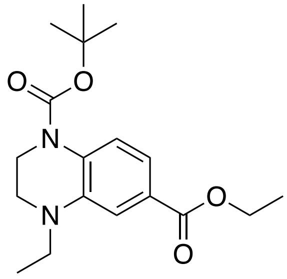

Sodium triacetoxyborohydride (831 mg, 3.92 mmol) was added to a solution of 1-(*tert*-butyl) 6-ethyl 3,4-dihydroquinoxaline-1,6(2*H*)-dicarboxylate **S-G** (200 mg, 0.65 mmol), acetaldehyde (147 μL, 2.61 mmol), and acetic acid (224 μL, 3.92 mmol) in CH_2Cl_2 (4.08 mL, 0.16 M). The reaction mixture was stirred at room temperature for 20 h. After concentration, the product was taken up with EtOAc (10 mL) and washed with saturated $NaHCO_3$ (15 mL). The aqueous layer was extracted with EtOAc (3 x 15 mL), and the combined organic layers were dried over Na_2SO_4. The crude product was purified by flash column chromatography (5% grading to 7% EtOAc/pentane) to give ethyl amine **S-H** (144 mg, 66%) as a brown oil.

R_f 0.44 (10% EtOAc/pentane)

ν_{max} (film)/cm^{-1} 2975, 2933, 1697, 1509, 1365, 1286, 1247, 1162, 1145, 1107, 764;

^{1}H NMR (400 MHz, $CDCl_3$) δ 7.40 (d, *J* = 8.4 Hz, 1 H), 7.26 (d, *J* = 1.8 Hz, 1 H), 7.20 (dd, *J* = 8.4, 1.8 Hz, 1 H), 4.25 (q, *J* = 7.1 Hz, 2 H), 3.70 – 3.67 (m, 2 H), 3.33 (q, *J* = 7.1 Hz, 2 H), 3.27 – 3.24 (m, 2 H), 1.42 (s, 9 H), 1.28 (t, *J* = 7.1 Hz, 3 H), 1.08 (t, *J* = 7.1 Hz, 3 H)

^{13}C NMR (101 MHz, $CDCl_3$) δ 167.1, 153.0, 137.5, 129.1, 126.6, 124.0, 116.9, 112.2, 81.6, 60.8, 47.5, 45.3, 41.4, 28.5, 14.5, 11.1;

HRMS (ESI$^+$) *m/z* Calculated for $C_{18}H_{27}N_2O_4^+$ $[M+H]^+$ 335.1971; Found 335.1983 (Δ = 3.6 ppm).

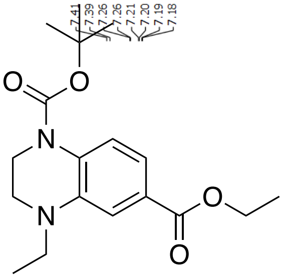

S-H

^{1}H NMR (400 MHz, $CDCl_3$)

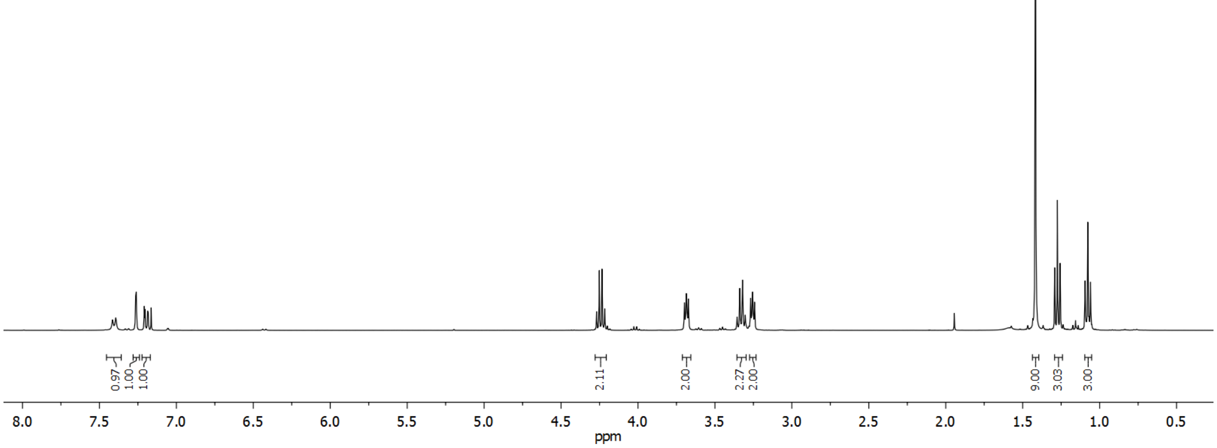

S-H

^{13}C NMR (101 MHz, $CDCl_3$)

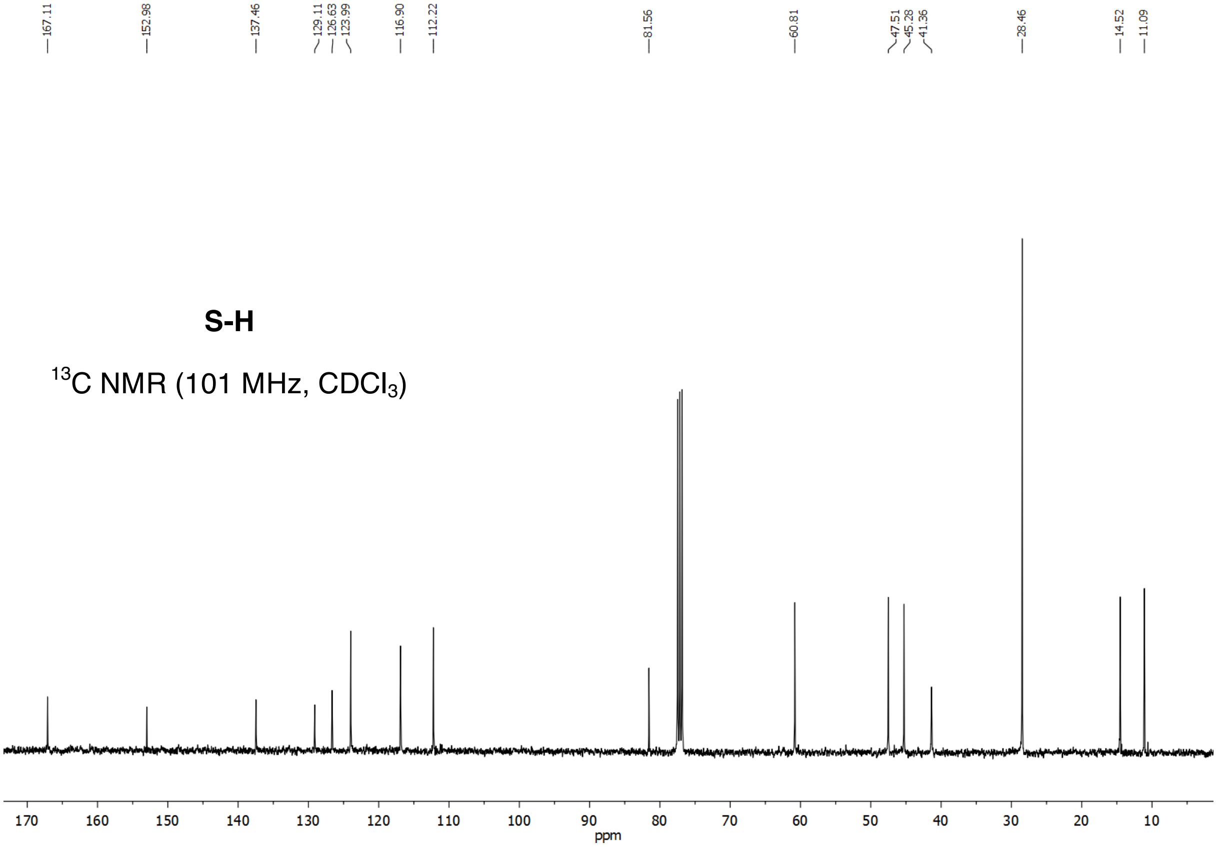

1-(*tert*-Butyl) 6-methyl 4-ethyl-3,4-dihydroquinoxaline-1,6(2*H*)-dicarboxylate (S-I)

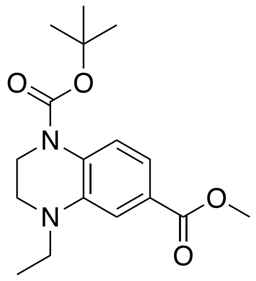

To a solution of 1-(tert-butyl) 6-ethyl 4-ethyl-3,4-dihydroquinoxaline-1,6(2H)-dicarboxylate **S-H** (34 mg, 0.10 mmol) in MeOH (1.5 mL, 0.07 M) at room temperature, K_2CO_3 (21 mg, 0.15 mmol) was added and the reaction mixture was stirred for 23 h at rt. The solution was diluted with EtOAc (2 mL) and the pH was adjusted to 7. The aqueous layer was extracted with EtOAc (4 x 15 mL), and the combined organic layers were dried over Na_2SO_4. Concentration *in vacuo* afforded the methyl ester **S-I** (30 mg, 91%) as a yellow oil.

R_f 0.34 (10% EtOAc/pentane)

ν_{max} (film)/cm^{-1} 2973, 1699, 1604, 1510, 1442, 1368, 1290, 1253, 1164, 1109, 1044, 1009, 860, 764;

^{1}H NMR (400 MHz, $CDCl_3$) δ 7.51 (d, *J* = 8.4 Hz, 1 H), 7.35 (d, *J* = 1.8 Hz, 1 H), 7.29 (dd, *J* = 8.4, 1.8 Hz, 1 H), 3.88 (s, 3 H), 3.80 – 3.77 (m, 2 H), 3.43 (q, *J* = 7.1 Hz, 2 H), 3.37 – 3.34 (m, 2 H), 1.52 (s, 9 H), 1.18 (t, *J* = 7.1 Hz, 3 H);

^{13}C NMR (101 MHz, $CDCl_3$) δ 167.6, 153.0, 137.5, 129.2, 126.3, 124.0, 117.0, 112.2, 81.6, 52.1, 47.5, 45.3, 41.4, 28.5, 11.1;

HRMS (ESI$^+$) *m/z* Calculated for $C_{17}H_{25}N_2O_4^+$ $[M+H]^+$ 321.1814; Found 321.1816 (Δ = 0.6 ppm).

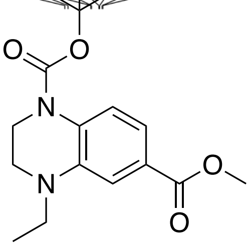

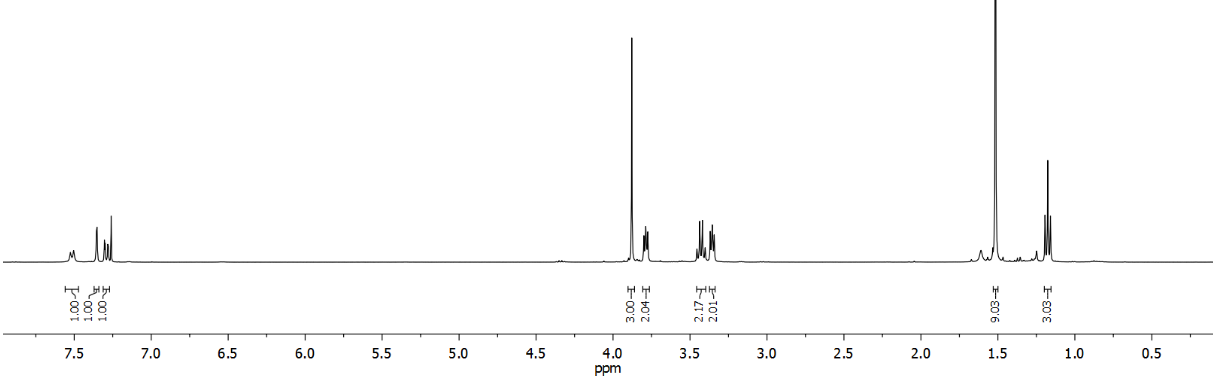

S-I

^{1}H NMR (400 MHz, $CDCl_3$)

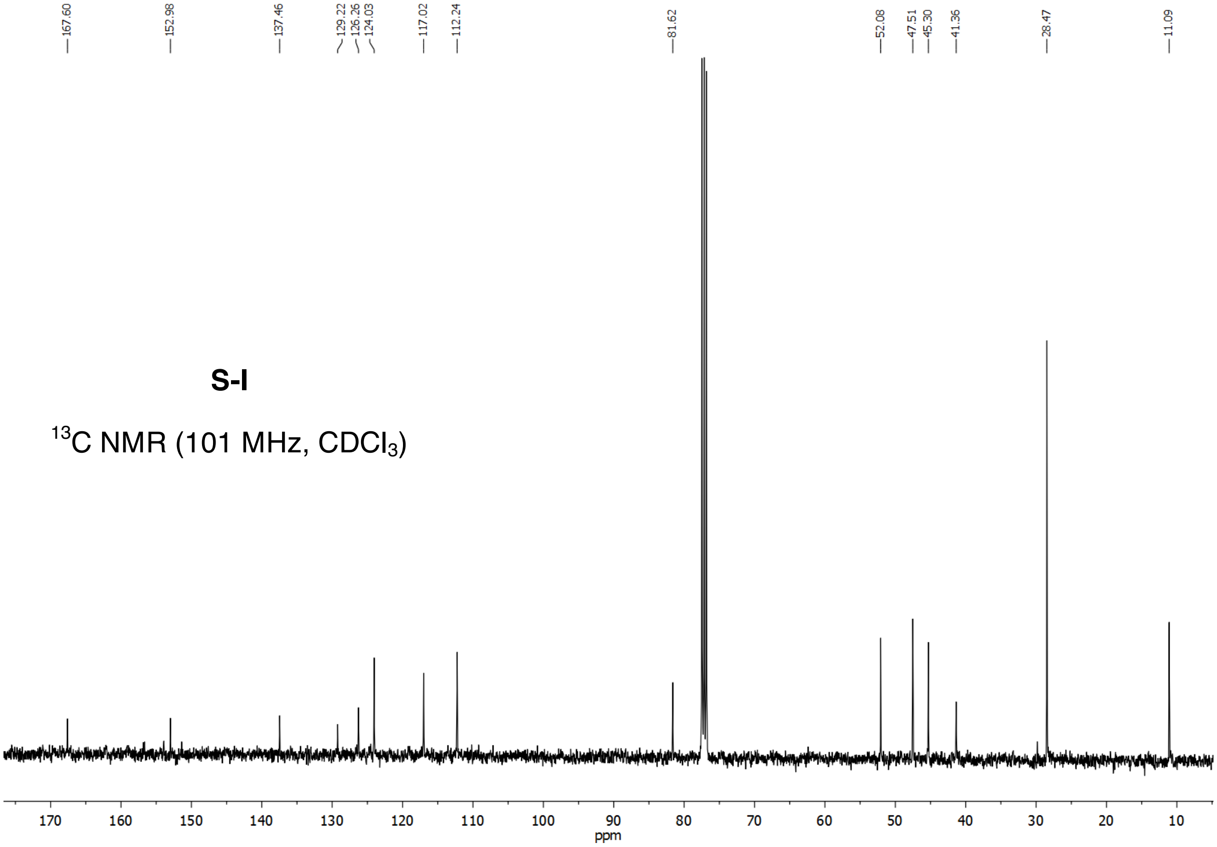

S-I

^{13}C NMR (101 MHz, $CDCl_3$)

Methyl 1-acryloyl-4-ethyl-1,2,3,4-tetrahydroquinoxaline-6-carboxylate (9)

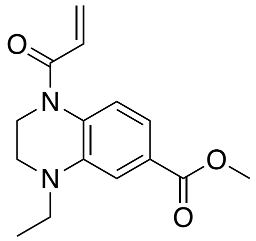

HCl (4 M in dioxane, 0.44 mL, 1.76 mmol) was added to 1-(tert-butyl) 6-methyl 4-ethyl-3,4-dihydroquinoxaline-1,6(2*H*)-dicarboxylate **S-I** (14.0 mg, 0.044 mmol). The solution was stirred for 5 h at room temperature. The solvent was removed under reduced pressure, affording a green solid. NEt_3 (15 µL, 0.11 mmol) was added to a solution of the green solid in chloroform (700 µL). A solution of acryloyl chloride (5.3 µL, 0.07 mmol) in chloroform (176 µL) was then added dropwise to the reaction mixture, which was stirred for 22 h at rt. Then the reaction was quenched with $NaHCO_3$ (10 mL) and the aqueous layer was extracted with CH_2Cl_2 (3 x 10 mL). The combined organic extracts were dried with Na_2SO_4 and filtered. The solvent was removed under reduced pressure. Purification by flash column chromatography (20% grading to 25% Et_2O/pentane) afforded tetrahydroquinoxaline **9** (5 mg, 37%) as a pale yellow solid.

m.p. = 74 ºC (CH_2Cl_2);

R_f 0.29 (30% Et_2O/pentane)

ν_{max} (film)/cm^{-1} 2362, 2353, 1718, 1657, 1609, 1511, 1443, 1411, 1363, 1291, 1254, 1185, 1110, 668;

^{1}H NMR (400 MHz, $CDCl_3$) δ 7.37 (d, *J* = 1.7 Hz, 1 H), 7.29 – 7.27 (m, 1 H), 6.97 (d, *J* = 8.1 Hz, 1 H), 6.62 (dd, *J* = 16.8, 10.2 Hz, 1 H), 6.47 (dd, *J* = 16.8, 2.0 Hz, 1 H), 5.73 (dd, *J* = 10.2, 2.0 Hz, 1 H), 3.99 – 3.96 (m, 2 H), 3.90 (s, 3 H), 3.48 – 3.41 (m, 4 H), 1.20 (t, *J* = 7.1 Hz, 3 H);

^{13}C NMR (101 MHz, $CDCl_3$) δ 167.3, 164.3, 138.3, 129.3, 128.7, 128.2, 128.2, 124.4, 116.6, 112.1, 52.3, 47.9, 44.9, 39.2, 11.1;

HRMS (ESI$^+$) *m/z* Calculated for $C_{15}H_{19}N_2O_3^+$ $[M+H]^+$ 275.1396; Found 275.1390 (Δ = -2.2 ppm).

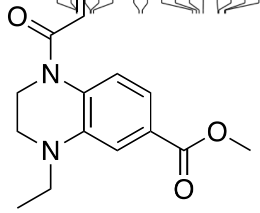

9

^{1}H NMR (400 MHz, $CDCl_3$)

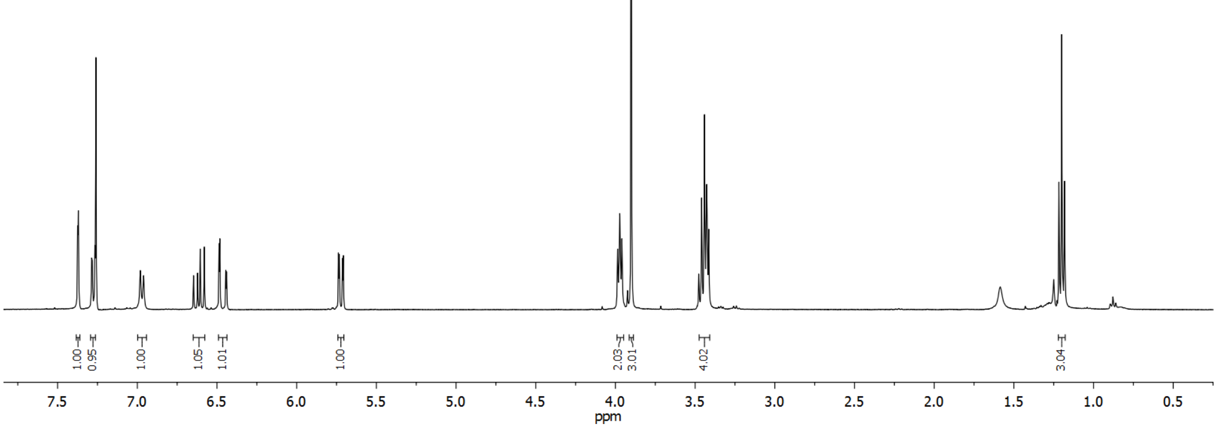

9

^{13}C NMR (101 MHz, $CDCl_3$)

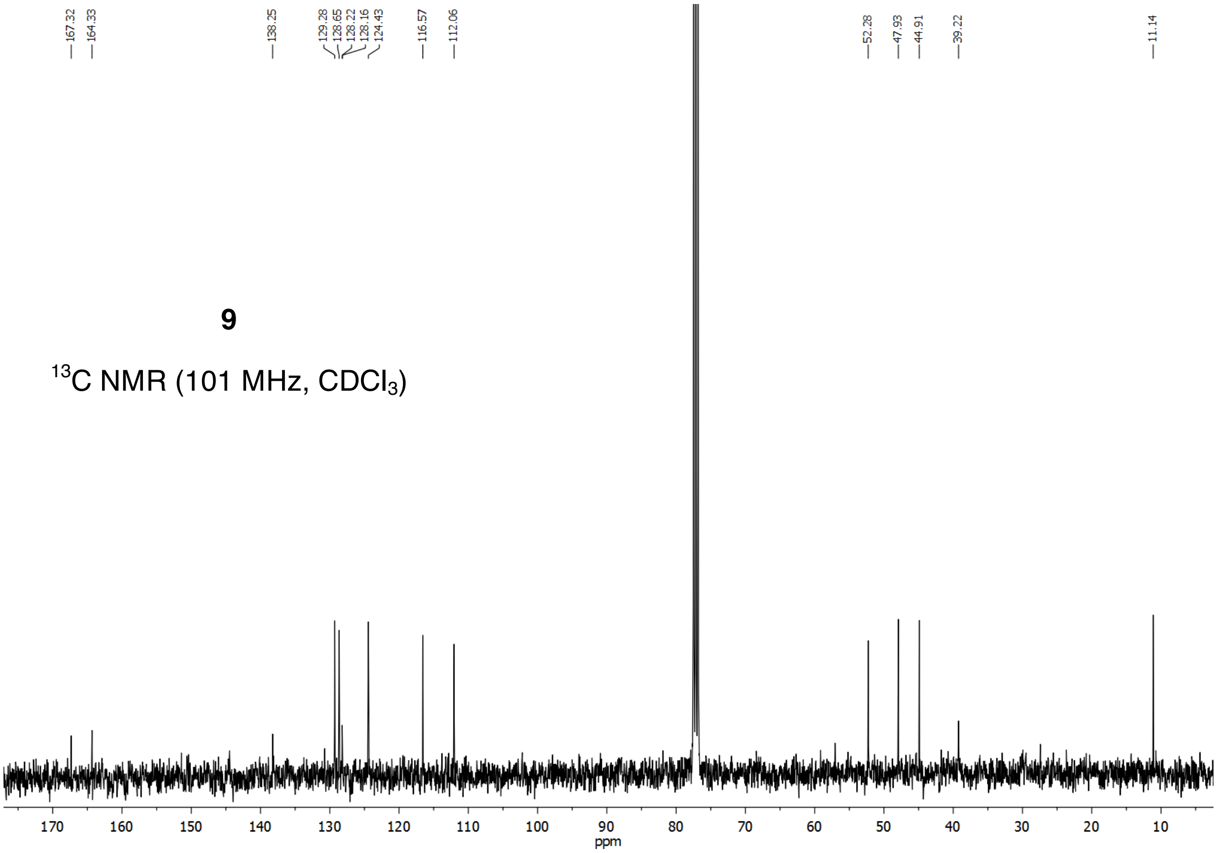

1-(*tert*-Butyl) 6-ethyl 4-methyl-3,4-dihydroquinoxaline-1,6(2*H*)-dicarboxylate (S-J)

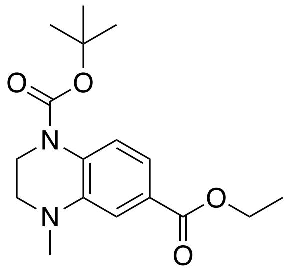

Sodium triacetoxyborohydride (415 mg, 1.96 mmol) was added to a solution of 1-(*tert*-butyl) 6-ethyl 3,4-dihydroquinoxaline-1,6(2*H*)-dicarboxylate **S-G** (100 mg, 0.326 mmol), formaldehyde (aq. 37% w/w, 35.9 µL, 1.30 mmol), and acetic acid (112 µL, 1.96 mmol) in CH_2Cl_2 (2.0 mL, 0.16 M). The reaction mixture was stirred at room temperature for 20 h. After concentration, the product was taken up with EtOAc (5 mL) and washed with saturated $NaHCO_3$ (5 mL). After extraction with EtOAc (3 x 10 mL), it was dried over Na_2SO_4. Concentration *in vacuo* afforded quinoxaline **S-J** (104 mg, 99%) as a yellow solid.

m.p. = 96 ºC (CH_2Cl_2);

R_f 0.54 (10% EtOAc/pentane)

ν_{max} (film)/cm^{-1} 1703, 1605, 1511, 1389, 1366, 1335, 1316, 1265, 1240, 1210, 1165, 1149, 1110, 1038, 764;

^{1}H NMR (400 MHz, $CDCl_3$) δ 7.54 (d, *J* = 8.2 Hz, 1 H), 7.35 – 7.32 (m, 2 H), 4.33 (q, *J* = 7.1 Hz, 2 H), 3.82 – 3.79 (m, 2 H), 3.33 – 3.31 (m, 2 H), 2.97 (s, 3 H), 1.50 (s, 9 H), 1.36 (t, *J* = 7.1 Hz, 3 H);

^{13}C NMR (101 MHz, $CDCl_3$) δ 167.0, 152.9, 138.8, 129.4, 126.4, 123.9, 117.7, 112.3, 81.5, 60.7, 50.2, 41.9, 38.8, 28.4, 14.5;

HRMS (ESI$^+$) *m/z* Calculated for $C_{17}H_{25}N_2O_4^+$ $[M+H]^+$ 321.1814; Found 321.1818 (Δ = 1.2 ppm).

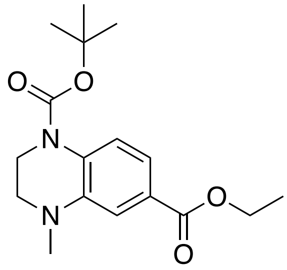

S-J

^{1}H NMR (400 MHz, $CDCl_3$)

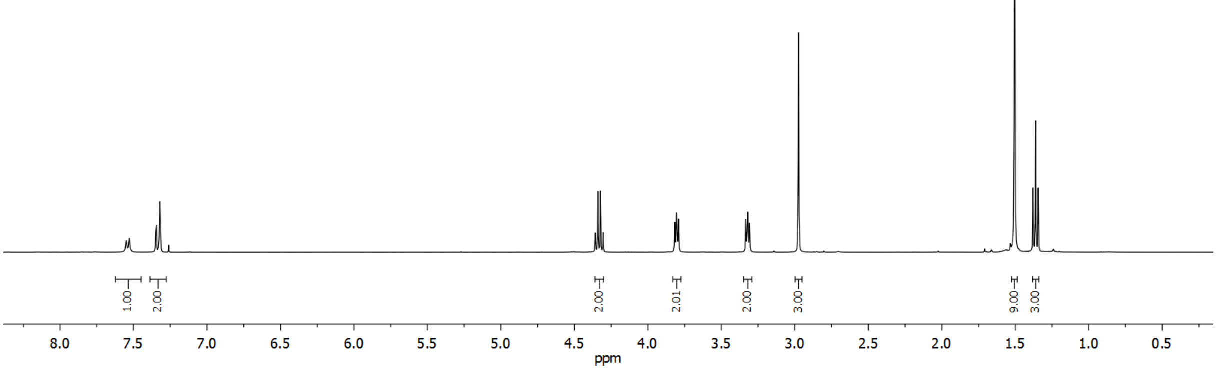

S-J

^{13}C NMR (101 MHz, $CDCl_3$)

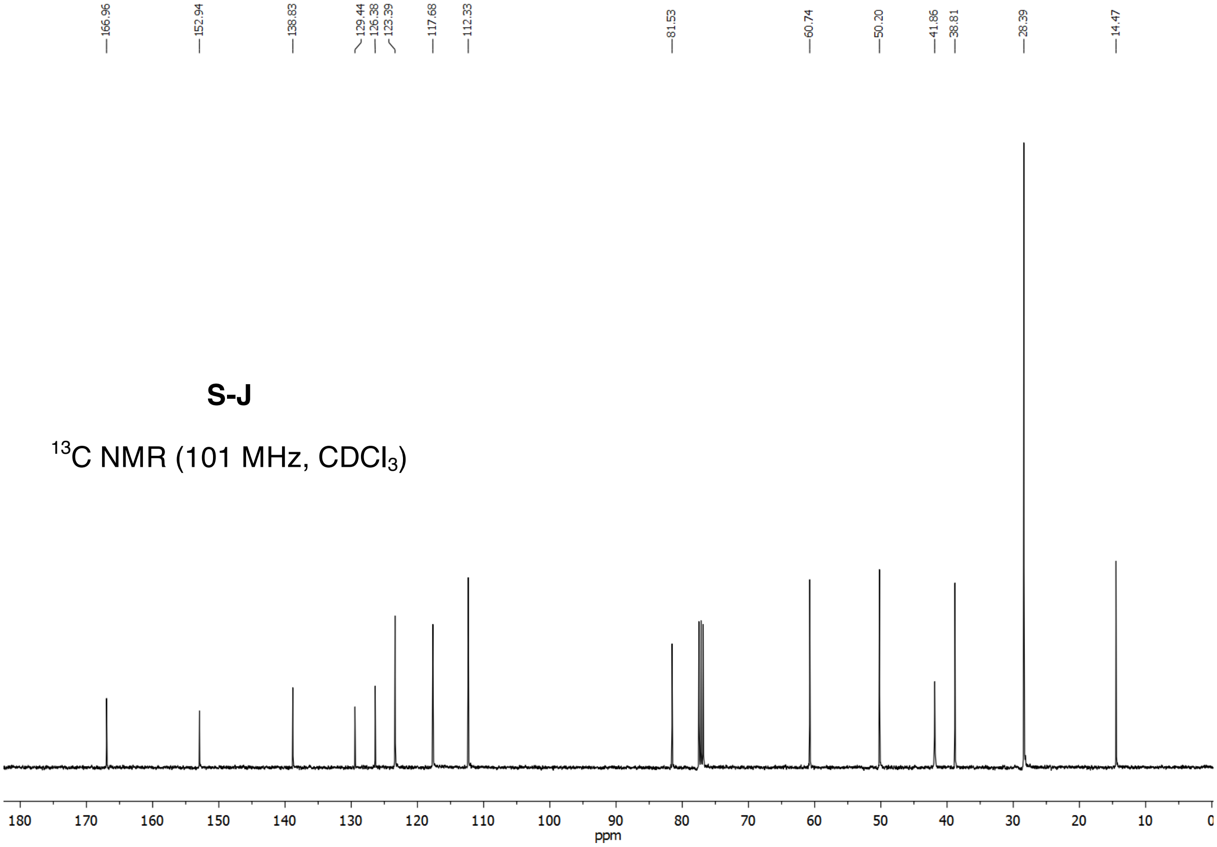

1-(*tert*-Butyl) 6-methyl 4-methyl-3,4-dihydroquinoxaline-1,6(2*H*)-dicarboxylate (S-K)

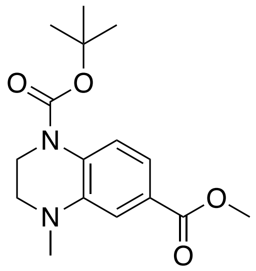

To a solution of 1-(tert-butyl) 6-ethyl 4-methyl-3,4-dihydroquinoxaline-1,6(2H)-dicarboxylate **S-J** (78 mg, 0.24 mmol) in MeOH (3.5 mL, 0.07 M) at room temperature, K_2CO_3 (501 mg, 0.37 mmol) was added and the reaction mixture was stirred for 21 h at rt. The solution was diluted with EtOAc (5 mL) and the pH was adjusted to 7. The aqueous layer was extracted with EtOAc (4 x 15 mL), and the combined organic layers were dried over Na_2SO_4. Concentration *in vacuo* afforded the methyl ester **S-K** (65 mg, 88%) as a yellow solid.

m.p. = 103 ºC ($CHCl_3$);

R_f 0.55 (10% EtOAc/pentane)

ν_{max} (film)/cm^{-1} 1712, 1701, 1605, 1512, 1391, 1367, 1336, 1269, 1240, 1211, 1165, 1149, 1114, 1039, 764;

^{1}H NMR (400 MHz, $CDCl_3$) δ 7.55 (d, *J* = 8.3 Hz, 1 H), 7.34 – 7.31 (m, 2 H), 3.87 (s, 3 H), 3.82 – 3.79 (m, 2 H), 3.34 – 3.31 (m, 2 H), 2.97 (s, 3 H), 1.50 (s, 9 H);

^{13}C NMR (101 MHz, $CDCl_3$) δ 167.5, 152.9, 138.9, 129.5, 126.0, 123.4, 117.7, 112.4, 81.6, 52.0, 50.2, 41.9, 38.8, 28.4;

HRMS (ESI$^+$) *m/z* Calculated for $C_{16}H_{23}N_2O_4^+$ [M+H]$^+$ 307.1658; Found 307.1664 (Δ = 2.0 ppm).

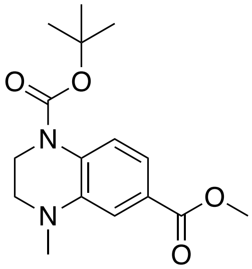

S-K

^{1}H NMR (400 MHz, $CDCl_3$)

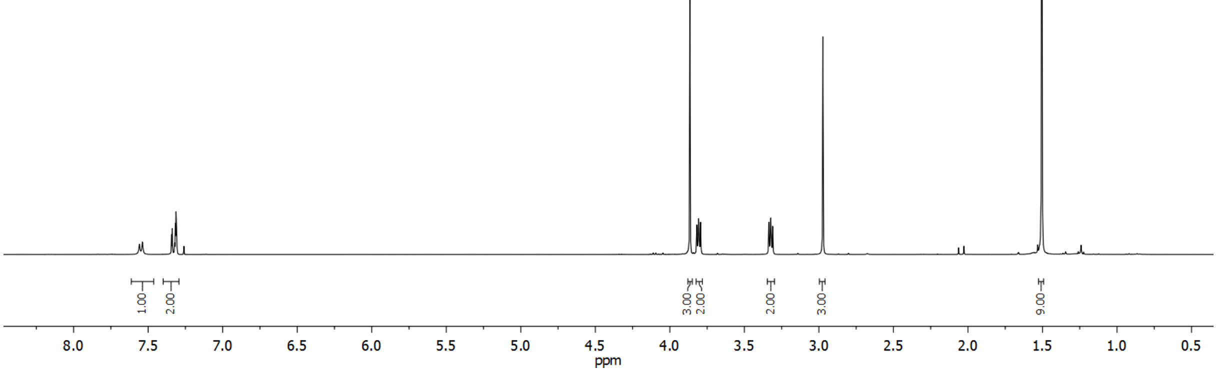

S-K

^{13}C NMR (101 MHz, $CDCl_3$)

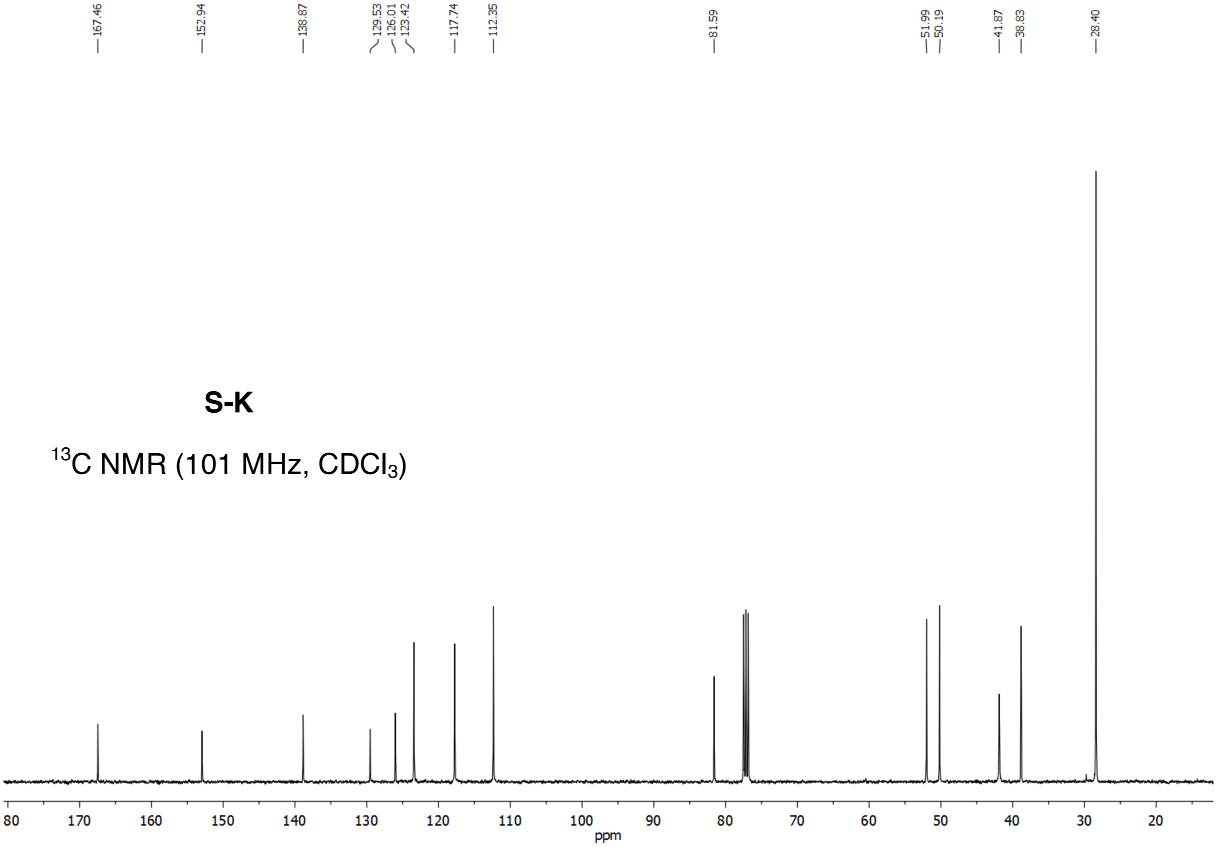

Methyl 1-acryloyl-4-methyl-1,2,3,4-tetrahydroquinoxaline-6-carboxylate (10)

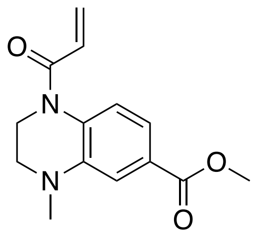

HCl (4 M in dioxane, 2.1 mL, 8.3 mmol) was added to 1-(tert-butyl) 6-methyl 4-methyl-3,4-dihydroquinoxaline-1,6(2*H*)-dicarboxylate **S-K** (63 mg, 0.21 mmol). The solution was stirred for 17 h at rt. The solvent was removed under reduced pressure, affording a green solid. NEt_3 (72 µL, 0.52 mmol) was added to a solution of the green solid in chloroform (3.1 mL). A solution of acryloyl chloride (25 µL, 0.31 mmol) in chloroform (1.0 mL) was then added dropwise to the reaction mixture which was stirred for 18 h at rt. Then the reaction was quenched with $NaHCO_3$ (10 mL) and the aqueous layer was extracted with CH_2Cl_2 (3 x 10 mL). The combined organic extracts were dried with Na_2SO_4 and filtered. The solvent was removed under reduced pressure. Purification by flash column chromatography (10% grading to 15% EtOAc/pentane) afforded tetrahydroquinoxaline **10** (37 mg, 66%) as a yellow solid.

m.p. = 110 ºC ($CHCl_3$);

R_f 0.18 (20% EtOAc/pentane)

ν_{max} (film)/cm^{-1} 1715, 1655, 1607, 1573, 1513, 1422, 1407, 1363, 1335, 1314, 1269, 1234, 1196, 1112, 762;

^{1}H NMR (400 MHz, $CDCl_3$) δ 7.33 – 7.29 (m, 2 H), 6.97 (d, *J* = 8.1 Hz, 1 H), 6.59 (dd, *J* = 16.8, 10.1 Hz, 1 H), 6.45 (dd, *J* = 16.8, 1.9 Hz, 1 H), 5.72 (dd, *J* = 10.1, 1.9 Hz, 1 H), 3.98 (t, *J* = 5.4 Hz, 2 H), 3.89 (s, 3H), 3.42 (t, *J* = 5.5 Hz, 2 H), 3.01 (s, 3 H);

^{13}C NMR (101 MHz, $CDCl_3$) δ 167.2, 164.4, 139.5, 129.2, 128.6, 128.4, 128.1, 123.9, 117.1, 112.1, 52.2, 50.6, 39.6, 38.2;

HRMS (ESI$^+$) *m/z* Calculated for $C_{14}H_{17}N_2O_3^+$ $[M+H]^+$ 261.1239; Found 261.1248 (Δ = 3.4 ppm).

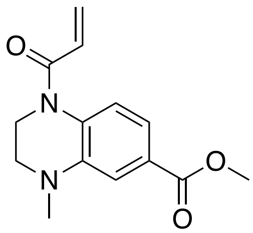

10

^{1}H NMR (400 MHz, $CDCl_3$)

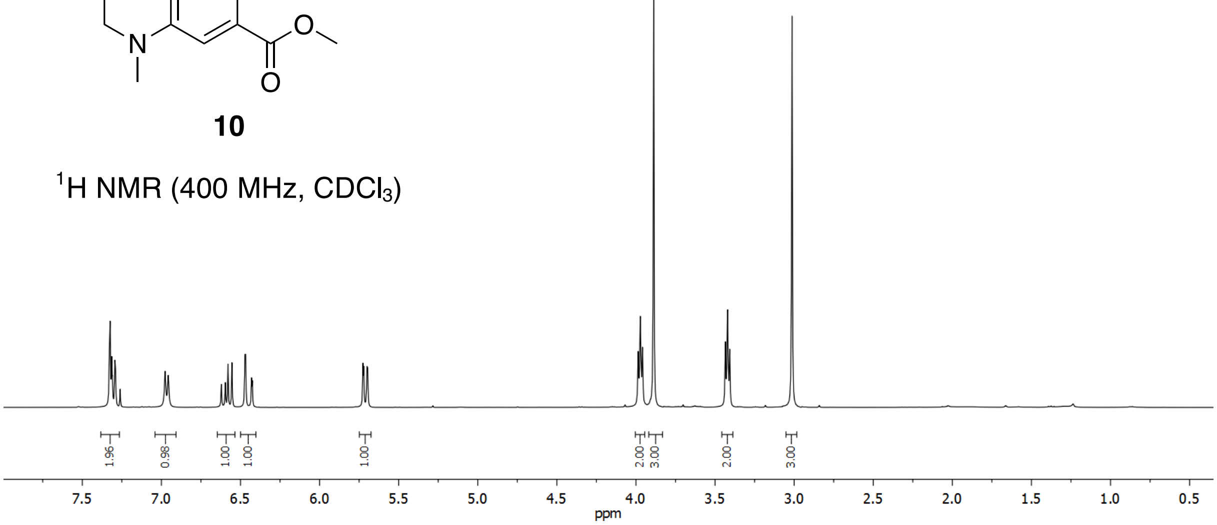

10

^{13}C NMR (101 MHz, $CDCl_3$)

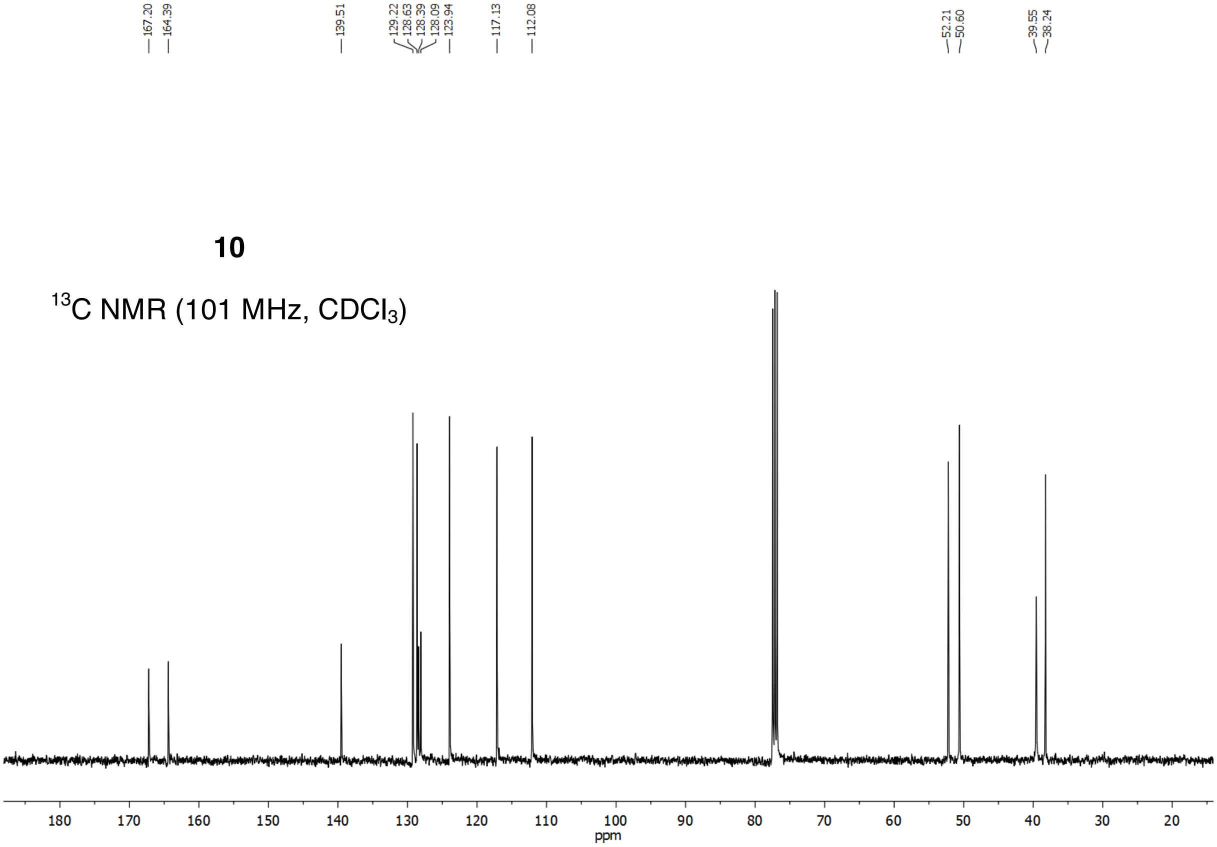

1-(*tert*-Butyl) 6-ethyl 4-(cyclopropylmethyl)-3,4-dihydroquinoxaline-1,6(2*H*)-dicarboxylate (S-L)

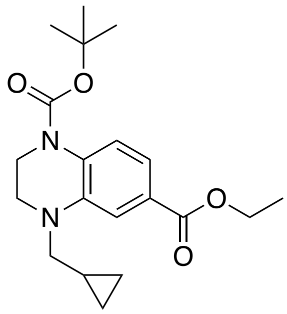

Sodium triacetoxyborohydride (415 mg, 1.96 mmol) was added to a solution of 1-(*tert*-butyl) 6-ethyl 3,4-dihydroquinoxaline-1,6(2*H*)-dicarboxylate **S-G** (100 mg, 0.326 mmol), cyclopropylcarboxylaldehyde (97.4 µL, 1.30 mmol), and acetic acid (112 µL, 1.96 mmol) in CH_2Cl_2 (2.04 mL, 0.16 M). The reaction mixture was stirred at room temperature for 18 h. After concentration, the product was taken up with EtOAc (10 mL) and washed with saturated $NaHCO_3$ (15 mL). After extraction with EtOAc (3 x 15 mL), it was dried over Na_2SO_4. Purification by flash column chromatography (2% grading to 5% EtOAc/pentane) afforded dihydroquinoxaline **S-L** (79 mg, 67%) as a yellow oil.

R_f 0.79 (10% EtOAc/pentane)

ν_{max} (film)/cm^{-1} 2360, 1703, 1604, 1510, 1442, 1367, 1343, 1281, 1242, 1161, 1112, 763, 668;

^{1}H NMR (400 MHz, $CDCl_3$) δ 7.53 (d, *J* = 8.4 Hz, 1 H), 7.45 (d, *J* = 8.1 Hz, 1 H), 7.31 (dd, *J* = 8.5, 1.9 Hz, 1 H), 4.34 (q, *J* = 7.1 Hz, 2 H), 3.81 – 3.78 (m, 2 H), 3.45– 3.43 (m, 2 H), 3.23 (d, *J* = 6.5 Hz, 2 H), 1.51 (s, 9 H), 1.37 (t, *J* = 7.1 Hz, 3 H), 1.10 – 1.02 (m, 1 H), 0.57 – 0.52 (m, 2 H), 0.24 (q, *J* = 4.8 Hz, 2 H);

^{13}C NMR (101 MHz, $CDCl_3$) δ 167.0, 152.9, 138.0, 129.1, 126.5, 123.9, 117.1, 112.7, 81.5, 60.7, 55.5, 48.3, 41.5, 28.4, 14.5, 8.6, 3.7;

HRMS (ESI$^+$) *m/z* Calculated for $C_{20}H_{29}N_2O_4^+$ $[M+H]^+$ 361.2127; Found 361.2128 (Δ = 0.3 ppm).

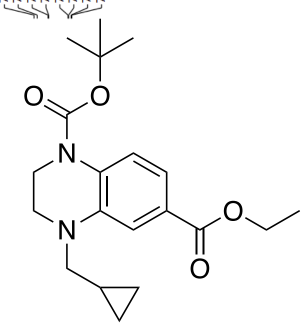

S-L

^{1}H NMR (400 MHz, $CDCl_3$)

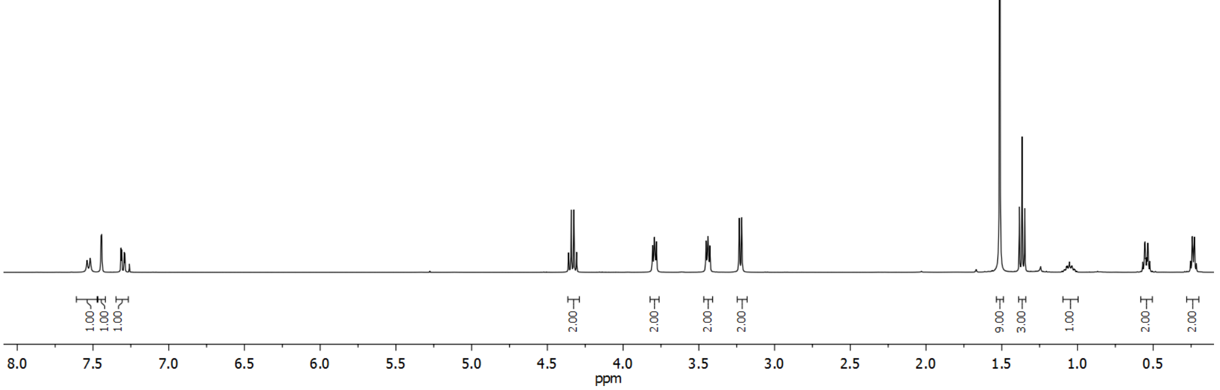

S-L

^{13}C NMR (101 MHz, $CDCl_3$)

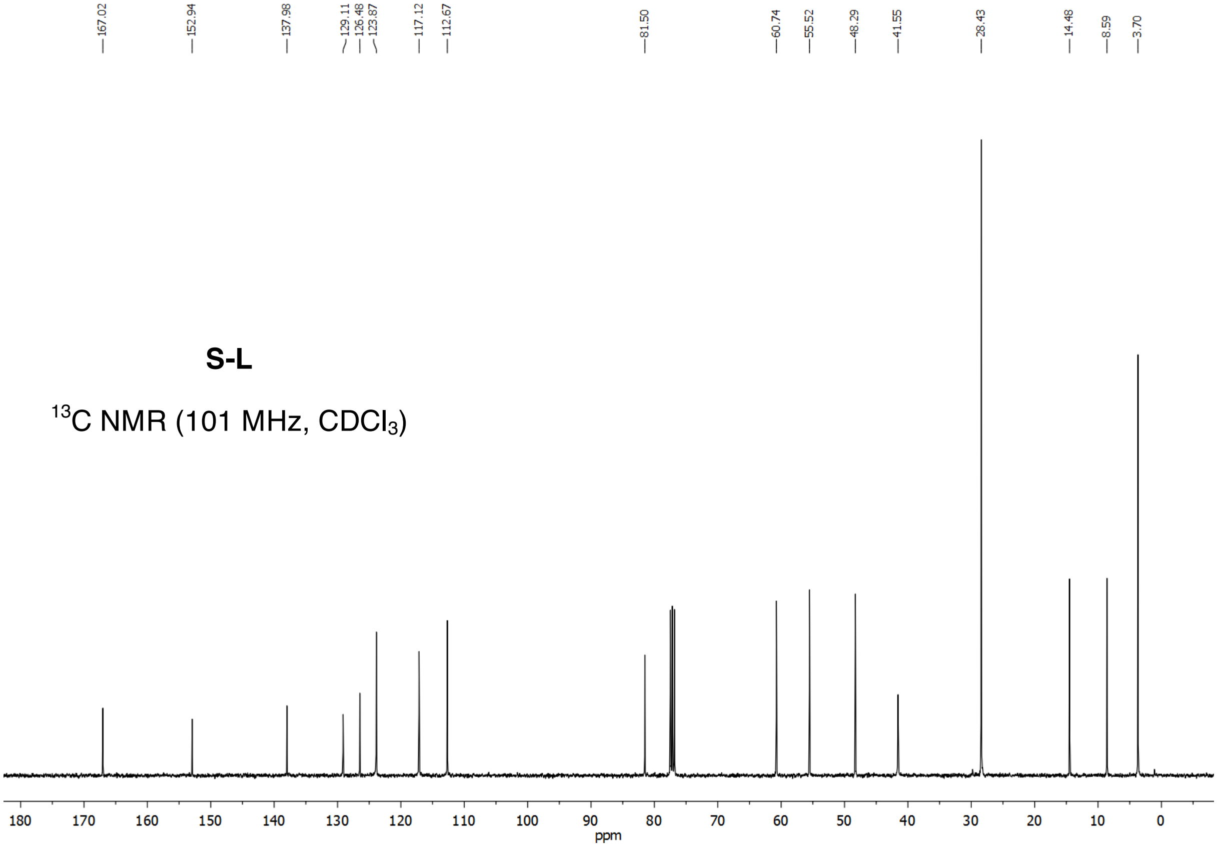

1-(*tert*-Butyl) 6-methyl 4-(cyclopropylmethyl)-3,4-dihydroquinoxaline-1,6(2*H*)-dicarboxylate (S-M)

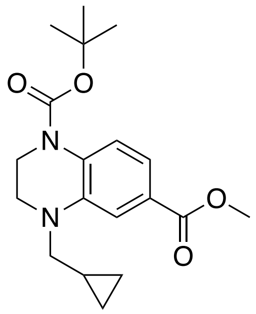

To a solution of 1-(tert-butyl) 6-ethyl 4-(cyclopropylmethyl)-3,4-dihydroquinoxaline-1,6(2H)-dicarboxylate **S-L** (71 mg, 0.20 mmol) in MeOH (2.8 mL, 0.07 M) at room temperature, K_2CO_3 (41 mg, 0.30 mmol) was added and the reaction mixture was stirred for 21 h at rt. The solution was diluted with EtOAc (5.0 mL) and the pH was adjusted to 7. The aqueous layer was extracted with EtOAc (4 x 15 mL), and the combined organic layers were dried over Na_2SO_4.Concentration *in vacuo* afforded the methyl ester **S-M** (66 mg, 96%) as a colourless oil.

R_f 0.62 (5% EtOAc/pentane)

ν_{max} (film)/cm^{-1}1706, 1603, 1573, 1511, 1441, 1368, 1284, 1244, 1161, 1115, 1052, 1010, 764;

^{1}H NMR (400 MHz, $CDCl_3$) δ 7.54 (d, *J* = 8.4 Hz, 1 H), 7.44 (d, *J* = 1.8 Hz, 1 H), 7.30 (dd, *J* = 8.5, 1.9 Hz, 1 H), 3.87 (s, 3 H), 3.81 – 3.78 (m, 2 H), 3.45– 3.43 (m, 2 H), 3.23 (d, *J* = 6.5 Hz, 2 H), 1.51 (s, 9 H), 1.09 – 1.02 (m, 1 H), 0.57 – 0.52 (m, 2 H), 0.24 (q, *J* = 4.8 Hz, 2 H);

^{13}C NMR (101 MHz, $CDCl_3$) δ 167.5, 152.9, 138.0, 129.2, 126.1, 123.9, 117.2, 112.6, 81.6, 55.5, 52.0, 48.3, 41.6, 28.4, 8.6, 3.7;

HRMS (ESI$^+$) *m/z* Calculated for $C_{19}H_{27}N_2O_4^+$ $[M+H]^+$ 347.1971; Found 347.1982 (Δ = 3.2 ppm).

S-M

^{1}H NMR (400 MHz, $CDCl_3$)

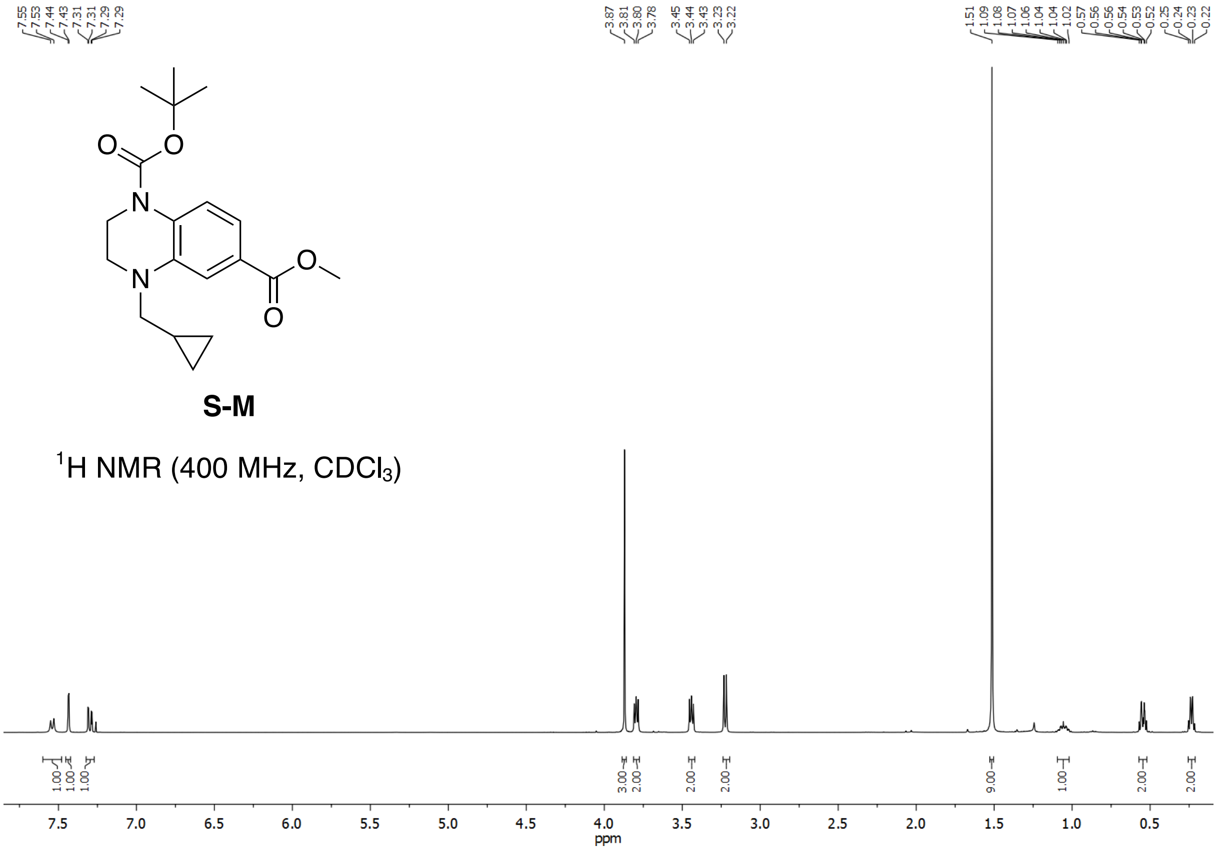

S-M

^{13}C NMR (101 MHz, $CDCl_3$)

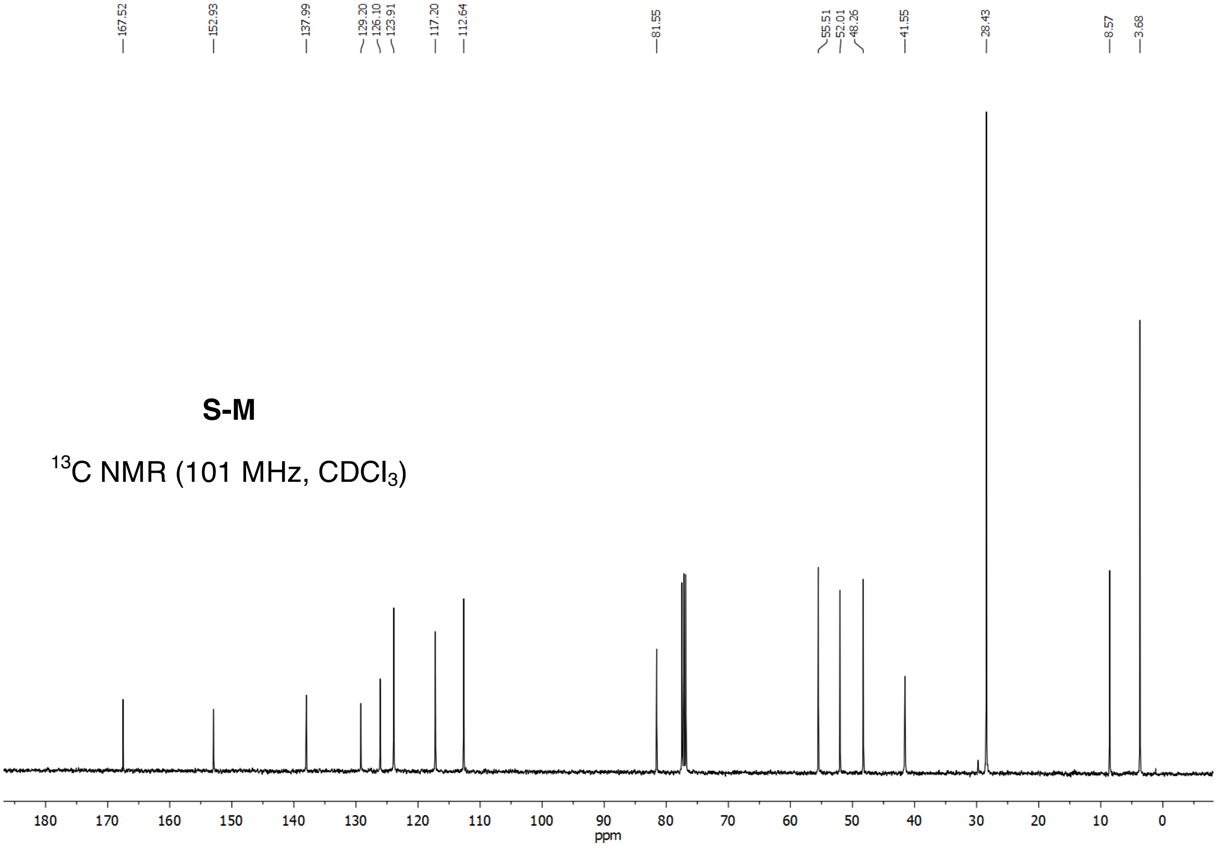

Methyl 1-acryloyl-4-(cyclopropylmethyl)-1,2,3,4-tetrahydroquinoxaline-6-carboxylate (11)

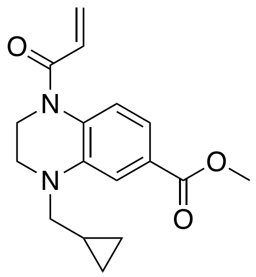

HCl (4 M in dioxane, 1.7 mL, 6.8 mmol) was added to 1-(tert-butyl) 6-methyl 4-(cyclopropylmethyl)-3,4-dihydroquinoxaline-1,6(2*H*)-dicarboxylate **S-M** (59 mg, 0.17 mmol). The solution was stirred for 16 h at rt. The solvent was removed under reduced pressure, affording a green solid. NEt_3 (59 µL, 0.42 mmol) was added to a solution of the green solid in chloroform (2.6 mL). A solution of acryloyl chloride (21 µL, 0.25 mmol) in chloroform (800 µL) was then added dropwise to the reaction mixture which was stirred for 18 h at rt. Then the reaction was quenched with $NaHCO_3$ (10 mL) and the aqueous layer was extracted with CH_2Cl_2 (3 x 10 mL). The combined organic extracts were dried with Na_2SO_4 and filtered. The solvent was removed under reduced pressure. Purification by flash column chromatography (10% EtOAc/pentane) afforded tetrahydroquinoxaline **11** (38 mg , 75% over) as a yellow oil.

R_f 0.39 (20% EtOAc/pentane)

ν_{max} (film)/cm^{-1} 1716, 1656, 1604, 1572, 1510, 1442, 1410, 1363, 1340, 1311, 1282, 1247, 1184, 1113, 763

^{1}H NMR (400 MHz, $CDCl_3$) δ 7.45 – 7.44 (m, 1 H), 6.28 (d, *J* = 8.1 Hz, 1 H), 6.97 (d, *J* = 8.1 Hz, 1 H), 6.62 (dd, *J* = 16.8, 10.2 Hz, 1 H), 6.46 (dd, *J* = 16.8, 1.9 Hz, 1 H), 5.72 (dd, *J* = 10.2, 1.9 Hz, 1 H), 3.98 (t, *J* = 5.3 Hz, 2 H), 3.89 (s, 3 H), 3.52 (t, *J* = 5.4 Hz, 2 H), 3.26 (d, *J* = 6.5 Hz, 2 H), 1.09 – 1.02 (m, 1 H), 0.57 (q, *J* = 5.6 Hz, 2 H), 0.25 (q, *J* = 5.0 Hz, 2 H);

^{13}C NMR (101 MHz, $CDCl_3$) δ 167.3, 164.3, 138.8, 129.3, 128.6, 128.1, 128.1, 124.4, 116.7, 112.5, 55.1, 52.2, 48.7, 39.4, 8.7, 3.7;

HRMS (ESI$^+$) *m/z* Calculated for $C_{17}H_{21}N_2O_3^+$ $[M+H]^+$ 301.1552; Found 301.1552 (Δ = 0.0 ppm).

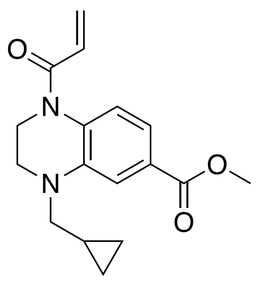

11

^{1}H NMR (400 MHz, $CDCl_3$)

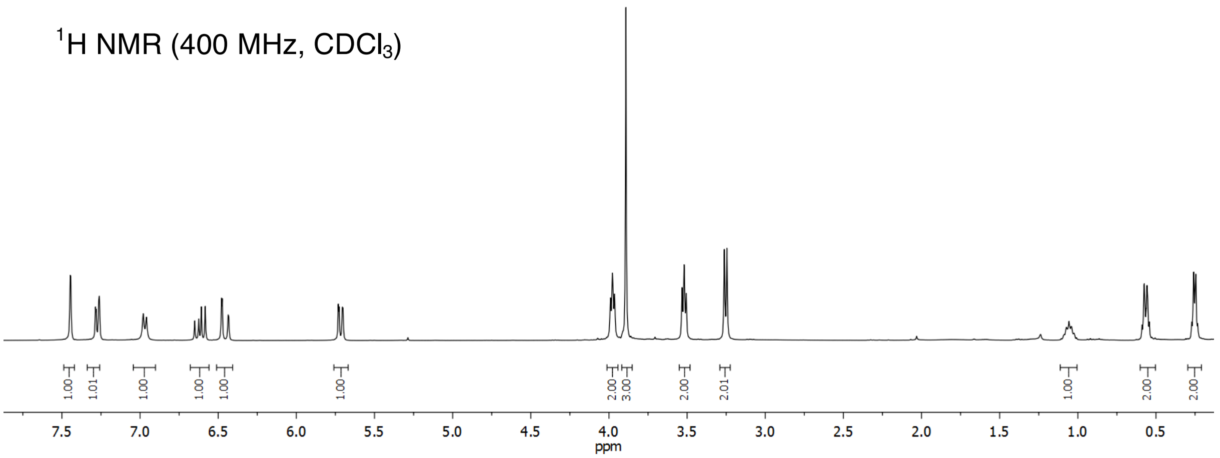

11

^{13}C NMR (101 MHz, $CDCl_3$)

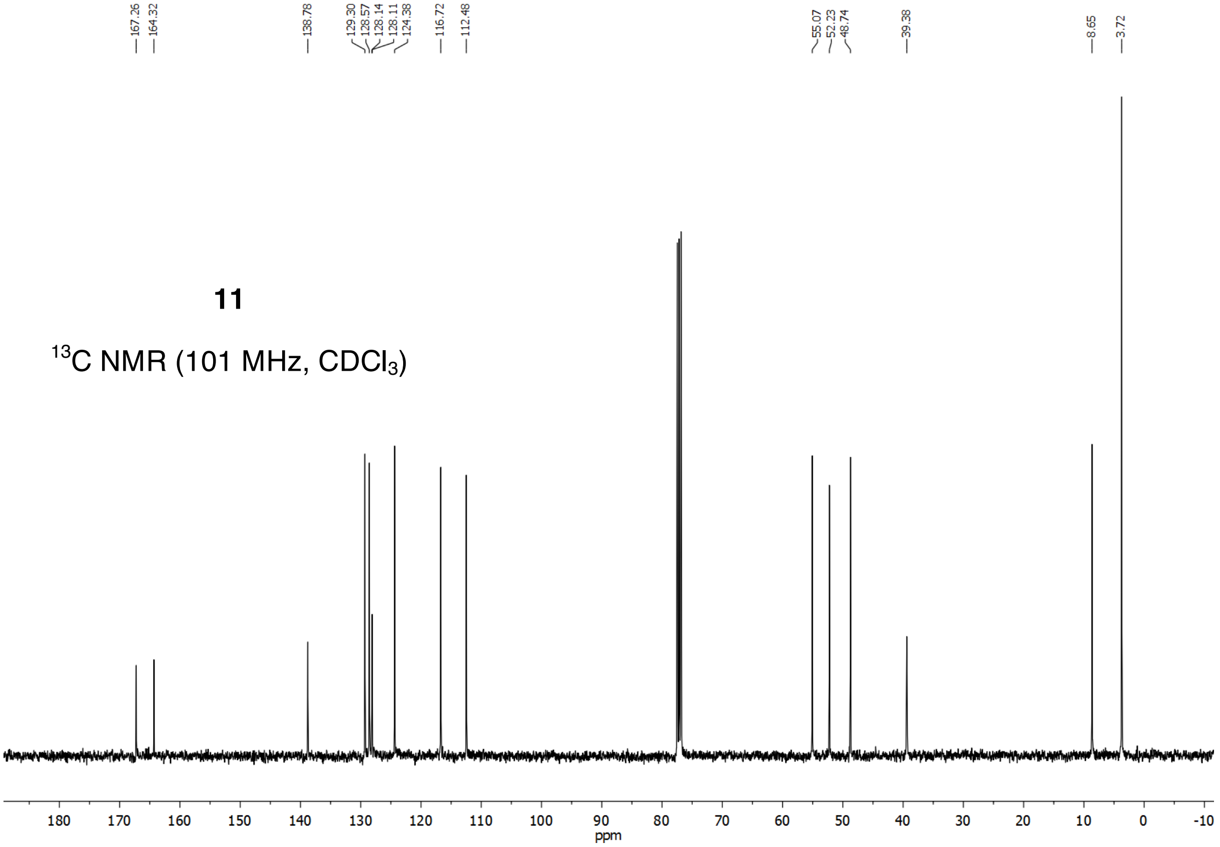

C. Library design

See *High-Throughput Kinetic Analysis for Target-Directed Covalent Ligand Discovery* for full library details.[4] In brief, Acrylamide fragments were synthesized from amine precursors; 14,000 primary and secondary amines were selected from Enamine's commercial collection with molecular weight < 250 Da, from which 235 unique Bemis-Murcko frameworks were generated.[5] For 100 of the frameworks, the lowest-molecular weight example was purchased. The corresponding acrylamides were then synthesized in a one step acrylation reaction (see *Efficient and Facile Synthesis of Acrylamide Libraries for Protein-Guided Tethering* for full synthetic details).[Error! Bookmark not defined.] The rest of the library consisted of commercially available electrophilic fragments, with warheads including epoxide, chloroacetamide, vinylsulfone, chloropyridine and cyanamide. The physiochemical properties of the final library were calculated using DataWarrior[6] and found to broadly comply with the 'Rule of 3' guidelines[7].

D. Rate determination by qIT

Reaction-plate setup: Each well of a 96-well PCR plate was charged with 60–100 µL of target thiol (GSH or protein, 10 µM) and 4% w/v TCEP-agarose beads in 100 mM phosphate buffer (pH 7.5 or 8) and cooled to 4 ˚C. Reactions were started by addition of an equal volume of pre-cooled electrophile solution (1 mM, 2% DMSO) in 0.1 M phosphate buffer (final concentration: thiol = 5 µM, electrophile = 0.5 mM, 2% w/v TCEP-agarose beads), with a minimum of 4 wells per plate used as DMSO/thiol only controls. After mixing, TCEP-agarose was pelleted by centrifugation (1,300 rpm, 1 min) and the plate incubated at 4 ˚C. *CPM Quench*: At a series of time points (typically around 0.25, 0.5, 1, 2, 6, 24, 48, 72 and 120 hours after reaction initiation), 3 µL aliquots of each reaction were quenched into a 384-well fluorescence plate (Corning – black, NBS) where each well was charged with 27 µL of CPM solution (1.39 µM) in 100 mM phosphate buffer (pH 7.5) (final concentration: thiol = 0.5 µM, CPM = 1.25 µM). Quenching was typically performed in duplicate and fluorescence plates were incubated at room temperature for 60 minutes before fluorescence intensity (excitation/emission: 384/470 nm) was measured on an EnVision™ plate reader. *Analysis*: All analysis was performed using Prism 8.0 software (GraphPad). For each time point, the fluorescence of each reaction was normalized relative Cdk2(ES)/GSH only control = maximum signal, DMSO only = minimum signal. The normalized fluorescence for each reaction was plotted against time. A one phase exponential decay was fitted to each plot (Constraints: Y(0) > 0.8; 0 < plateau = 0), yielding a pseudo first-order rate constant.

E. Protein expression

Cdk2 mutations were introduced by PCR and cloned into pGEX6P1 Cdk2 (552), a gift from Jonathon Pines (Addgene plasmid # 61845) used to express GST-Cdk2(WT). GST-Cdk2(ES) = GST-Cdk2(F80C,C177A). All constructs were verified by sequencing. pGEX6P1 cyclin A2 (amino acids 173-432) was generated by PCR and restriction digestion. GST-CAK1 was constructed by PCR amplification of *S.cerevisiae* genomic DNA and the product cloned into pKEG-KG. All protein expression was performed using an adapted autoinduction protocol:[8] Plasmids were transformed into E. coli Tuner(DE3) and cultures grown in autoinduction media at 37˚C until OD_{600} = 0.9. The cultures were then incubated at 18˚C for a further 40 hours before harvesting by centrifugation (10 min, 6000 g).

F. Protein purification

GST-Cdk2(ES) and GST-cyclin A: Harvested cells were resuspended in 50 mM HEPES buffer (pH7.5) containing 150 mM NaCl, 10 mM $MgCl_2$, 2 mM dithiothreitol (DTT), 0.5 mg mL^{-1} lysozyme, and 0.01% Triton X-100 at 4 ˚C and incubated for 30 minutes. After sonication and centrifugation (40 min, 16000 g), the supernatant was incubated for 1 h with glutathione-Sepharose beads, washed with lysis buffer and the desired protein eluted with 10 mM GSH (100 mM Tris, pH 8). After incubation the desired protein was cleaved from the GST tag with PreScission protease (100:1) at 4˚C for 4 hours, the cleaved GST tag was removed with glutathione-Sepharose beads. Cdk2 was concentrated and then loaded onto a Superdex 75 (16/60) column, eluting with 100 mM phosphate buffer containing 50 mM DTT. Purified Cdk2 was concentrated to 10 mg mL^{-1} and stored at –80 ˚C. Cyclin A2 was purified by glutathione-Sepharose affinity as described above with the following amendments: 100 mM $MgCl_2$ was added after GST cleavage to minimise protein aggregation and gel filtration was performed with 50 mM HEPES buffer (pH 7.5) containing 150 mM NaCl, 100 mM $MgCl_2$, 2 mM DTT. GST-CAK1 was also purified using the glutathione-Sepharose affinity workflow, however no protease or gel filtration steps were performed and the enzyme was used crude.

G. Acrylamide labelling of Cdk2 for crystallography, kinase assays and intact-protein mass spectrometry

Cdk2 (10 µM) was reacted with acrylamide ligands at 500 µM in 100 mM phosphate buffer (pH 8) containing 1% DMSO, until labelling reached completion (as monitored by fluorescence quench assay and/or intact protein mass spectrometry). Excess ligand was subsequently removed by serial dilution and concentration (×5) into the appropriate buffer using Amicon Ultra-0.5 centrifugal filter devices (MW cut-off = 10 kDa).

H. Intact-protein mass spectrometry

Protein-mass data were obtained on a Waters micromass LC-TOF mass spectrometer in positive ion mode using electrospray ionization. Samples were chromatographed using an Acquity UPLC protein BEH C4 column (2.1 mm × 50 mm, 2.7 µm) with a flow rate of 30 µL/min. The injection volume was 5 µL of protein solution (5 µM) in 30 mM ammonium bicarbonate buffer containing 0.5% formic acid and 20% MeOH. The gradient used was 100% mobile phase A (0.1% formic acid in water/MeOH (19:1)) ramping linearly to 90% mobile phase B (0.1% formic acid in MeOH) over 5 minutes. The spectra were acquired from 500 to 2500 Da and deconvolution was performed with MassLynx (Waters), using the maximum entropy algorithm, over a mass range of 800 to 1200 Da with a mass step of 0.75.

I. Crystallography

All crystallization was performed using the hanging drop method at 21˚C and crystals were harvested in cryoprotectant: 30% w/v Jeffamine ED-2003, 100 mM HEPES, pH 7.5. *Apo-Cdk2:* Crystallisation was performed by mixing 1 µL of Cdk2(WT) solution (8 mg mL^{-1}, 100 mM phosphate buffer, pH 6.2 containing 1 mM DTT) with 1 µL of reservoir solution (30% w/v Jeffamine ED-2003, 100 mM HEPES, pH range 7.5 – 8.5). Crystals appeared overnight (typical dimensions: 150 $µm^3$) and diffracted to 1.2 Å. *Microseed preparation*: Microseeds were generated using a glass seed bead kit (Hampton Research) according to the manufacturer's description. In brief, three apo-Cdk2(WT) crystals were pipetted into the seed glass tube, containing 50 µL of reservoir solution (30% w/v Jeffamine ED-2003, 100 mM HEPES, pH 7.5), and vortexed twice for ten seconds. An additional 450 µL of reservoir solution was added to form a high-concentration seed stock. Empirically, it was found that an additional 100-fold dilution gave the optimum seed concentration for subsequent crystallization. *Ligand conjugated Cdk2(ES)*: Crystallisation was performed by mixing 1 µL of labelled Cdk2(ES) solution (8 mg mL^{-1}, 100 mM phosphate buffer) with 0.5 µL of reservoir solution (Jeffamine ED-2003 concentration range 18 - 30% w/v, 100 mM HEPES, pH range 7.5 – 8.5) and 0.5 µL diluted apo-Cdk2(WT) microseed stock. Crystals appeared within 24 hours.

J. Data collection and structure determination

X-ray diffraction data were collected on the synchrotron beam lines i03 and i04 at Diamond Light Source, Didcot, and processed using XDS[9] and implemented within Xia2[10]. Data collection and refinement statistics are summarized in Supplementary Tables 2-6. Molecular replacement was conducted on all data sets using Phaser[11]/MOLREP[12] against apo-Cdk2 (PDB: 4EK3) and the model was iteratively refined using Phenix.Refine[13] with manual modelling and adjustments carried out in COOT[14]. Descriptions of the ligands and links were generated with JLigand[15] and refined with Refmac[16]. Electron density maps were calculated using FFT[17] and figures were prepared using PyMol (Schrödinger).

K. Cdk2 phosphorylation

Cdk2(WT) or Cdk2(ES) (1 mg) was sequentially incubated with aliquots of CAK1 (3 × 0.05 mg) at r.t. for 4 hours at a time in 1.5 mL of a 30 mM Tris/HCl buffer (pH 7.5) containing 10 mM $MgCl_2$, 10 mM ATP and 3 mM DTT. As CAK1 appeared to become rapidly inactivated in the presence of pCdk2, three repetitions were required to drive the reaction to completion. The resulting solution was incubated with 50 µL of glutathione-Sepharose beads and filtered to remove remaining CAK1.

L. Kinase assay

Kinase assays were performed by coupling the conversion of ATP into ADP to the oxidation of NADH (Abs = 340 nm) into NAD^+, mediated by lactate dehydrogenase (LDH) and pyruvate kinase (PK). WT, mutant or ligand-conjugated pCdk2 (12.5 or 50 nM) was preincubated with Cyclin A2 (20 nM) in a buffer containing 50 mM Tris/HCl, 10 mM $MgCl_2$, 5 mM DTT, 1 mM phosphoenolpyruvate, 0.24 mM NADH, 10 U mL^{-1} LDH, 14 U mL^{-1} PK and 5% DMSO at 37 ˚C for 30 minutes. Reactions were initiated by addition of 0.5 mM ATP and 0.24 mM substrate peptide (PKTPKKAKKL) and the absorbance at 340 nm was monitored for two hours. Assays were conducted at 37˚C and carried out in clear 384-well plates (Nunc) using a SpectraMax i3x plate reader (Molecular Devices) with a final volume of 20 µL in each well. After plotting Abs_{340} against time, enzyme velocity was measured from the gradient during the linear phase of product accumulation.

Q. Concentration dependent qIT

The rate of reaction (k_{obs}) for the reaction between each acrylamide (25, 50, 125, 250 and 500 µM) with Cdk2(ES) (5 µM) was determined us qIT (see *D. Rate Determination by qIT*) in duplicate. The average k_{obs} was plotted against acrylamide concentration and hyperbolic regression analysis performed using Prism 8.0 software (GraphPad) to derive k_{inact} and K_i.

Supplementary Reference

[1] J. Ackermann, K. Bleicher, S. M. Ceccarelli, O. Chomienne, P. Mattei, S. U. Obst, *Novel Bicyclic Sulfonamide Derivatives Which Are L-CPT1 Inhibitors*, **2010**, US2011046112.

[2] Allen, C. E. *et al.* Efficient and Facile Synthesis of Acrylamide Libraries for Protein-Guided Tethering. *Org. Lett.* **17,** 458–460 (2015).

[3] Zhang, L. *et al.* Cu(ii)-catalyzed C–H (SP3) oxidation and C–N cleavage: base-switched methylenation and formylation using tetramethylethylenediamine as a carbon source. *Chem. Commun.* **48,** 5928 (2012).

[4] G. B. Craven, D. P. Affron, C. E. Allen, S. Matthies, J. G. Greener, R. M. L. Morgan, E. W. Tate, A. Armstrong, D. J. Mann, *Angew. Chemie Int. Ed.* **2018**, *57*, 5257–5261.

[5] G. W. Bemis, M. A. Murcko, *J. Med. Chem.* **1996**, *39*, 2887–2893.

[6] T. Sander, J. Freyss, M. von Korff, C. Rufener, *J. Chem. Inf. Model.* **2015**, *55*, 460–473.

[7] M. Congreve, R. Carr, C. Murray, H. Jhoti, *Drug Discov. Today* **2003**, *8*, 876–877.

[8] F. W. Studier, *Protein Expr. Purif.* **2005**, *41*, 207–234.

[9] W. Kabsch, *Acta Crystallogr. Sect. D Biol. Crystallogr.* **2010**, *66*, 125–132.

[10] G. Winter, *J. Appl. Crystallogr.* **2010**, *43*, 186–190.

[11] A. J. McCoy, R. W. Grosse-Kunstleve, P. D. Adams, M. D. Winn, L. C. Storoni, R. J. Read, *J. Appl. Crystallogr.* **2007**, *40*, 658–674.

[12] A. A. Lebedev, A. A. Vagin, G. N. Murshudov, *Acta Crystallogr. Sect. D Biol. Crystallogr.* **2008**, *64*, 33–39.

[13] P. V. Afonine, R. W. Grosse-Kunstleve, N. Echols, J. J. Headd, N. W. Moriarty, M. Mustyakimov, T. C. Terwilliger, A. Urzhumtsev, P. H. Zwart, P. D. Adams, *Acta Crystallogr. Sect. D Biol. Crystallogr.* **2012**, *68*, 352–367.

[14] P. Emsley, B. Lohkamp, W. G. Scott, K. Cowtan, *Acta Crystallogr. Sect. D Biol. Crystallogr.* **2010**, *66*, 486–501.

[15] A. A. Lebedev, P. Young, M. N. Isupov, O. V Moroz, A. A. Vagin, G. N. Murshudov, *Acta Crystallogr. Sect. D Biol. Crystallogr.* **2012**, *68*, 431–440.

[16] G. N. Murshudov, A. A. Vagin, E. J. Dodson, *Acta Crystallogr. Sect. D Biol. Crystallogr.* **1997**, *53*, 240–255.

[17] G. N. Murshudov, A. A. Vagin, A. Lebedev, K. S. Wilson, E. J. Dodson, *Acta Crystallogr. Sect. D Biol. Crystallogr.* **1999**, *55*, 247–255.
